# Major Outbreaks in the Nineteenth Century Shaped Grape Phylloxera Contemporary Genetic Structure in Europe

**DOI:** 10.1038/s41598-019-54122-0

**Published:** 2019-11-26

**Authors:** Javier Tello, Roswitha Mammerler, Marko Čajić, Astrid Forneck

**Affiliations:** 1 Department of Crop Sciences, Institute of Viticulture and Pomology, University of Natural Resources and Applied Life Sciences Vienna, Konrad Lorenz Str. 24, A-3430 Tulln, Austria; 2 Faculty of Agricultural Sciences, University of Zagreb, Svetošimunska cesta 25, 10000 Zagreb, Croatia

**Keywords:** Population genetics, Entomology, Invasive species

## Abstract

Grape phylloxera is native to North America, where *Vitis* spp. acquired different mechanisms of resistance to leaf and root attack. Its appearance in European vineyards at the beginning of the 1860s, where the phylloxera-susceptible grapevine species *V. vinifera* L. is majorly cultivated, caused the devastation of a great number of vineyards, generating a deep crisis in the European wine production and trade industries. However, the origin and genetic structure of this pest across European vineyards still remain controversial and uncertain. Herein, we analysed the genetic structure of 1173 grape phylloxera individuals collected from 100 locations across eight European countries. Structure and phylogenetic analyses show that contemporary grape phylloxera populations in Europe are the result of at least two independent introductions from the native range that mirrors the historical records that also suggest two major outbreaks in Europe. The comparative analysis with samples from the native range trace back one of these two genetic groups to plants imported from the North East coast of North America, where the American species *V. riparia* and *V. labrusca* dominate. This study clarifies the level of genetic diversity of grape phylloxera in Europe and provides relevant information to resolve previous controversy about its origin.

## Introduction

*Daktulosphaira vitifoliae* Fitch (commonly known as grape phylloxera) is the most geographically spread pest species, virtually occurring in all viticultural regions around the world^1^. Grape phylloxera is an obligate biotroph of *Vitis* spp., capable of feeding on both grapevine roots and leaves^2^. Its native range extends from southern Canada to northern South America, but it is more common in the eastern and central USA^3^. The coevolution of grape phylloxera and American grapevine *Vitis* spp. led to the generation of different grapevine species with different strategies of resistance to grape phylloxera leaf and root attack^4^. Because grape phylloxera was not present in the centre of origin of cultivated grapes and Europe before the nineteenth century, the cultivated grapevine (*Vitis vinifera* L.) did not develop any resistance mechanism^1,4^. In susceptible *V. vinifera* cultivars, grape phylloxera forms abundant root nodosities and tuberosities that occlude the vascular system of the vine, resulting in a dramatic loss of leaf surface area and yield. In addition, feeding wounds allow secondary infections by soil-borne pathogens and cause plants to die from secondary infections^1,2^. The natural capability of grape phylloxera to move among vineyards to infest healthy plants is limited^5^, but human-assisted dispersal can inadvertently transport insects between sites through infested plant material, machinery, clothing and footwear, postharvest grape products and winery wastes^1^. It is generally accepted that the insect was unintentionally introduced into Europe at the end of the nineteenth century through infected plant material from North America to fight oidium (powdery mildew), the fungus that threatened European vineyards in the 1850s^6^. Grape phylloxera appearance almost destroyed European viticulture, and it provoked the most radical switch in viticultural practices of the last two centuries, when grape growing changed from the use of own-rooted *V. vinifera* plants to their grafting onto partially-resistant American non-*vinifera Vitis* spp. or hybrids used as rootstocks^7^.

Historical records show that grape phylloxera appeared in Europe at the beginning of the 1860s in the southern Rhône region of France (Pujaut, La Crau-St-Rémy, Graveson), where vines began to wither and die for no previously-known causes. Nevertheless, it was not until 1868 that the presence of grape phylloxera in grapevine dead roots was certified by a commission of experts, who initially denominated the insect as *Phylloxera vastatrix*^8^. Almost simultaneously, the insect was observed in the Bordeaux region, with an outbreak registered in 1869 in the term of Floirac (Gironde)^9^. Soon after, grape phylloxera become ubiquitous all over southern and central vineyards of France^10,11^, where it destroyed about 40% of vines, inducing an important economic shock in a society largely dependent on agriculture^12^. From this main focus, grape phylloxera advanced southward towards Spain^13^ and Italy^14^, and northward towards the wine regions of Geneva and St. Gallen in Switzerland^10^. Between 1874 and 1900, diverse outbreaks were reported in several locations in the Rhine, Moselle, Neckar and Main river valleys (Germany)^10^. At the same time, two important additional grape phylloxera outbreaks were registered in very distant regions. During the 1860s, wine producers reported a new pest affecting Douro’s vines in Portugal, with withering vines detected in vineyards of the municipalities of Sabrosa, Santa Marta de Penaguião, Peso da Régua and São João da Pesqueira. In spring of 1863, the initial focus of this grape phylloxera outbreak was found in a vineyard of Gouvinhas, probably as a result of the introduction of some American vine cuttings imported by the owner of the vineyard^15^. Simultaneously, another major outbreak was observed in 1868 in the city of Klosterneuburg (near Vienna, Austria), which was officially registered in 1872^16^. From there, grape phylloxera spread following the Danube river route, first to neighbouring Austrian viticultural areas of Lower Austria and Burgenland, and then to modern Czechia, Slovakia and Hungary^10^. After 1880, grape phylloxera was observed in viticultural regions of Serbia, Bulgaria and Romania, reaching Moldavia in 1887 and the Crimean Peninsula soon after^10^.

In spite of being a matter of concern for European grape growers and scientists for more than 150 years, the genetic structure of grape phylloxera in Europe is still unclear. Although several reports dealing with grape phylloxera populations at a national level can be found^17–19^, few studies have dealt with this issue at a multinational scale^20,21^. Nevertheless, the use of low resolution genetic markers^20^ or the screening of a limited number of populations from a reduced number of sampling sites^21^ hindered obtaining sound conclusions on the genetic structure of this pest in Europe. Better understanding of genetic diversity of grape phylloxera in Europe provides critical information to develop monitoring and control strategies for this pest in European vineyards in near future. The goal of this study is to analyse the spatial genetic structure of grape phylloxera infesting commercial and abandoned vineyards in Europe. For this purpose, we investigated the association between the insect and geography in conjunction with historical information to understand contemporary population structure patterns. We carried out extensive sampling of grape phylloxera populations from 100 locations spread over eight European countries (Austria, Croatia, Germany, Hungary, Italy, Romania, Serbia and Switzerland). In addition, we included some grape phylloxera samples from the introduced regions of South Africa and Uruguay. They were jointly analysed with individuals from the native range to evaluate the point of origin of the populations nowadays present in Europe.

## Results

### Grape phylloxera genotyping and identification of multilocus genotypes (MLGs)

A set of 1173 leaf-feeding and root-feeding grape phylloxera individuals collected from Europe (Austria, Croatia, Germany, Hungary, Italy, Romania, Serbia and Switzerland) and two other introduced regions (South Africa and Uruguay) from a diverse variety of hosts (*V. vinifera* L. cultivars, *Vitis* spp. interspecific rootstocks, *Vitis* spp. interspecific hybrid direct-producers and *Vitis* spp. interspecific resistant grape varieties) were genotyped with 7 SSR markers (Table 1). A low number of missing data was obtained, with only 16 individuals presenting one missing locus, three individuals with two missing loci, and four individuals with three missing loci. Thus, the percentage of not-genotyped loci ranged from 0.0% to 1.45% (for *PhyIII_30* and *DVSSR4* loci, respectively). All the SSR markers were polymorphic among grape phylloxera samples, with a mean number of 9.1 alleles per locus. The number of alleles ranged from 5 (for *DV8*) to 12 (for *PhyIII_55* and *DVSSR4*). The combination of this genetic information led to the identification of 774 unique MLGs (Supplementary Table S1). Whilst 610 grape phylloxera samples were identified only once (single MLGs), 164 MLGs were represented by 563 samples (repeated MLGs) (Table 2). The most abundant MLG was MLG16 that consisted of 18 samples collected from the leaves of *Vitis* spp. interspecific rootstocks in Austria, followed by the MLG380, represented by 16 leaf phylloxera samples collected from diverse *Vitis* spp. interspecific rootstocks and *Vitis* spp. interspecific hybrid direct-producers grown in the municipalities of Stankovci (Croatia) and Quinten (in Switzerland), separated by ca. 620 km. MLG380 was not the only case in which the same MLG was collected from sites separated by large distances. MLG113 was found in Nuβdorf ob der Traisen (Austria) and in Rudolfingen (Switzerland), separated by ca. 330 km. Similarly, we found MLG475 in Köveskál (Hungary) and in Rafz (Switzerland), locations separated by ca. 690 km. On the other hand, we found seven MLGs (MLG113, MLG318, MLG329, MLG360, MLG463, MLG469 and MLG521) feeding in both leaves and roots. In addition, several examples of the same MLG in *Vitis* spp. interspecific hybrid direct-producers and *Vitis* spp. interspecific rootstocks (MLG113, MLG380, MLG394, MLF469 and MLF475), in *Vitis* spp. interspecific hybrid direct-producers and *V. vinifera* L. cultivars (MLG247 and MLG381), and in *Vitis* spp. interspecific rootstocks and *V. vinifera* L. cultivars (MLG360, MLG714 and MLG730) were found (Supplementary Table S1).

**Table 1:** Origin of the grape phylloxera samples considered in this study.

	AUT	CHE	DEU	HRV	HUN	ITA	ROU	SRB	URY	ZAF
Hybrid direct-producer (leaf-feeding form)	—	117	72	—	—	—	—	—	—	—
Hybrid direct-producer (root-feeding form)	—	61	1	—	—	—	—	—	—	—
Resistant grape variety (leaf-feeding form)	7	10	—	—	—	—	—	12	—	—
Resistant grape variety (root-feeding form)	—	5	—	—	—	—	—	2	—	—
Rootstock (abandoned vineyard, leaf-feeding form)	343	—	—	63	49	37	3	2	—	—
Rootstock (abandoned vineyard, root-feeding form)	—	—	—	—	53	—	—	4	—	—
Rootstock (commercial vineyard, leaf-feeding form)	3	4	—	—	—	—	—	13	—	8
Rootstock (commercial vineyard, root-feeding form)	—	59	—	—	—	—	—	—	—	—
Rootstock (suckers, leaf-feeding form)	28	—	—	—	—	8	—	—	—	—
*Vitis vinifera* L. variety (leaf-feeding form)	10	20	39	35	—	34	—	5	44	—
*Vitis vinifera* L. variety (root-feeding form)	—	2	5	—	—	—	—	1	—	—
*Unknown*	—	5	—	9	—	—	—	—	—	—
**TOTAL**	**391**	**283**	**117**	**107**	**102**	**79**	**3**	**39**	**44**	**8**

Countries are named according to the ISO 3166-1 encoding list. AUT: Austria; CHE: Switzerland; DEU: Germany; HRV: Croatia; HUN: Hungary; ITA: Italy; ROU: Romania; SRB: Serbia; URY: Uruguay; ZAF: South Africa.

**Table 2:** Genetic diversity parameters obtained for the whole dataset (All) and the two grape phylloxera genetic groups identified in Europe by STRUCTURE (Eu1 and Eu2).

	All	Eu1	Eu2
Number of individuals (*N*)	1173	216	244
Number of MLGs	774	143	159
Genotypic diversity (*R* = (MLGs-1)/(*N*-1))	0.66	0.66	0.65
Clonal diversity index (*P*_*d*_ = MLGs/*N*)	0.66	0.66	0.65
Repeated genotypes	164	32	33
Genotypes with significant *P*_*sex*_ (<0.01)	159	32	33
Mean no. of alleles per locus	9.10	6.14	5.71
Mean *H*_*obs*_ per locus	0.54	0.52	0.51
Mean *H*_*e*_ per locus	0.61	0.57	0.57
Private alleles	—	10	13
***F***_***IS***_ **per locus**			
*PhyIII_55*	0.10	0.02	0.15
*PhyIII_30*	0.08	0.05	−0.18
*PhyIII_36*	0.03	−0.05	0.06
*DV8*	0.10	0.03	0.21
*Dvit6*	0.19	0.13	0.18
*DVSSR4*	0.13	0.13	−0.05
*DV4*	0.17	0.27	0.27

### Population structure analysis of grape phylloxera in Europe

**Figure 1:**
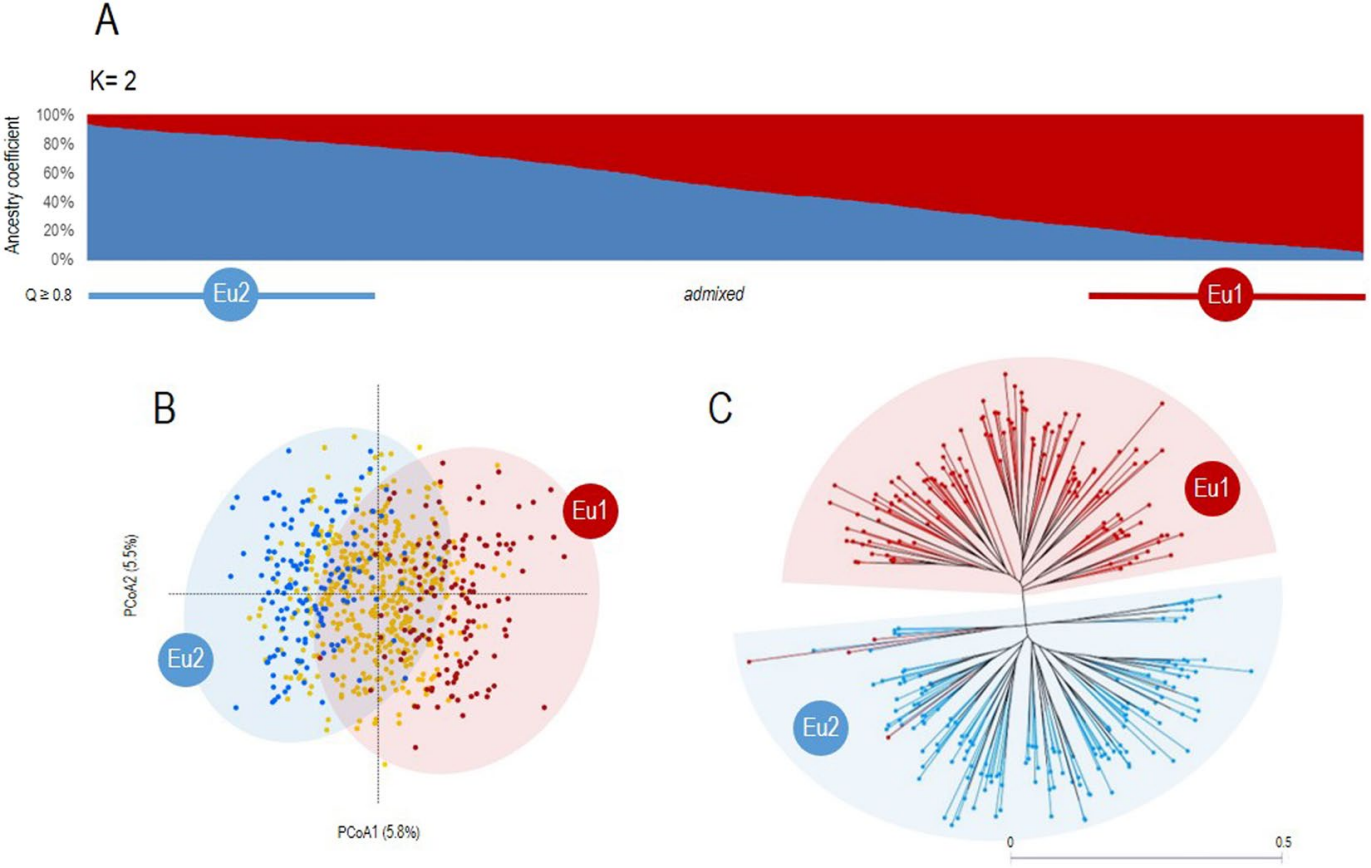
Population structure of grape phylloxera in Europe. In A, population structure results obtained with STRUCTURE^39^ considering 774 grape phylloxera MLGs and 7 SSR loci is shown. The optimal number of genetic groups (*K* = 2, Eu1 and Eu2) was set considering the method of Puechmaille^40^. Every MLG is shown as a vertical line, whose colour(s) indicates their estimated membership to Eu1 (red) or Eu2 (blue). Considering a critical ancestry coefficient of *Q* ≥ 0.80, 143 and 159 MLGs were assigned to Eu1 and Eu2, respectively (472 MLGs were considered as admixed). In B, a principal coordinate analysis (PCoA) obtained from a dissimilarity matrix calculated in DARwin from genetic data (7 SSRs) from 752 MLGs is shown. MLGs assigned to Eu1 and Eu2 are indicated as red and blue dots, respectively. Admixed MLGs are shown as yellow dots. The variance explained by the PCoA1 and PcoA2 is indicated (%). In C, the neighbor-joining unweighted dendrogram obtained in DARwin considering the MLGs grouped in Eu1 (red) and Eu2 (blue) according to STRUCTURE results is shown.

The genetic structure of the whole dataset was explored using STRUCTURE, PCoA and NJUw analyses. This study includes three variables for the samples: sampling site^20^, feeding form^22^ and host plant^3^). To avoid the impact of uneven sampling for these variables, STRUCTURE output was evaluated considering the *MedMeaK*, *MaxMeaK*, *MedMedK* and *MaxMedK* statistics to determine the most probable number of genetic groups. *MedMedK*, *MaxMedK* and *MaxMeaK* suggested *K* = 3 as the optimal level of structuring when using the sampling site (country) as correcting factor, whereas *MedMeaK* pointed out *K* = 2. On the other hand, the four metrics indicated *K* = 2 as the most probable number of genetic groups when using feeding form or host plant as co-factor (Supplementary Fig. S1). At *K* = 2, and considering a coefficient of ancestry (*Q*) over 0.80 for group assignation, 302 MLGs (39.0%) were associated with one of the two genetic groups (denominated Eu1 and Eu2), whereas 472 MLGs (61.0%) were identified as “admixed” (Fig. 1A). PCoA results supported this level of structuring, as the two genetic groups identified by the previous analysis (Eu1 and Eu2) could be differentiated according to PCoA1 loadings (Fig. 1B). The neighbor-joining unweighted dendrogram built for the 295 MLGs associated to either Eu1 or Eu2 (seven were excluded because of the presence of missing data) clearly supported the presence of two clusters (Fig. 1C). One of the clusters included 158 MLGs (2.5% assigned to Eu1 and 97.5% to Eu2), and the other cluster consisted of 137 MLGs (100% assigned to Eu1). Altogether, *K* = 2 was considered as the most likely level of structuring in our dataset; only these results are discussed.

**Figure 2:**
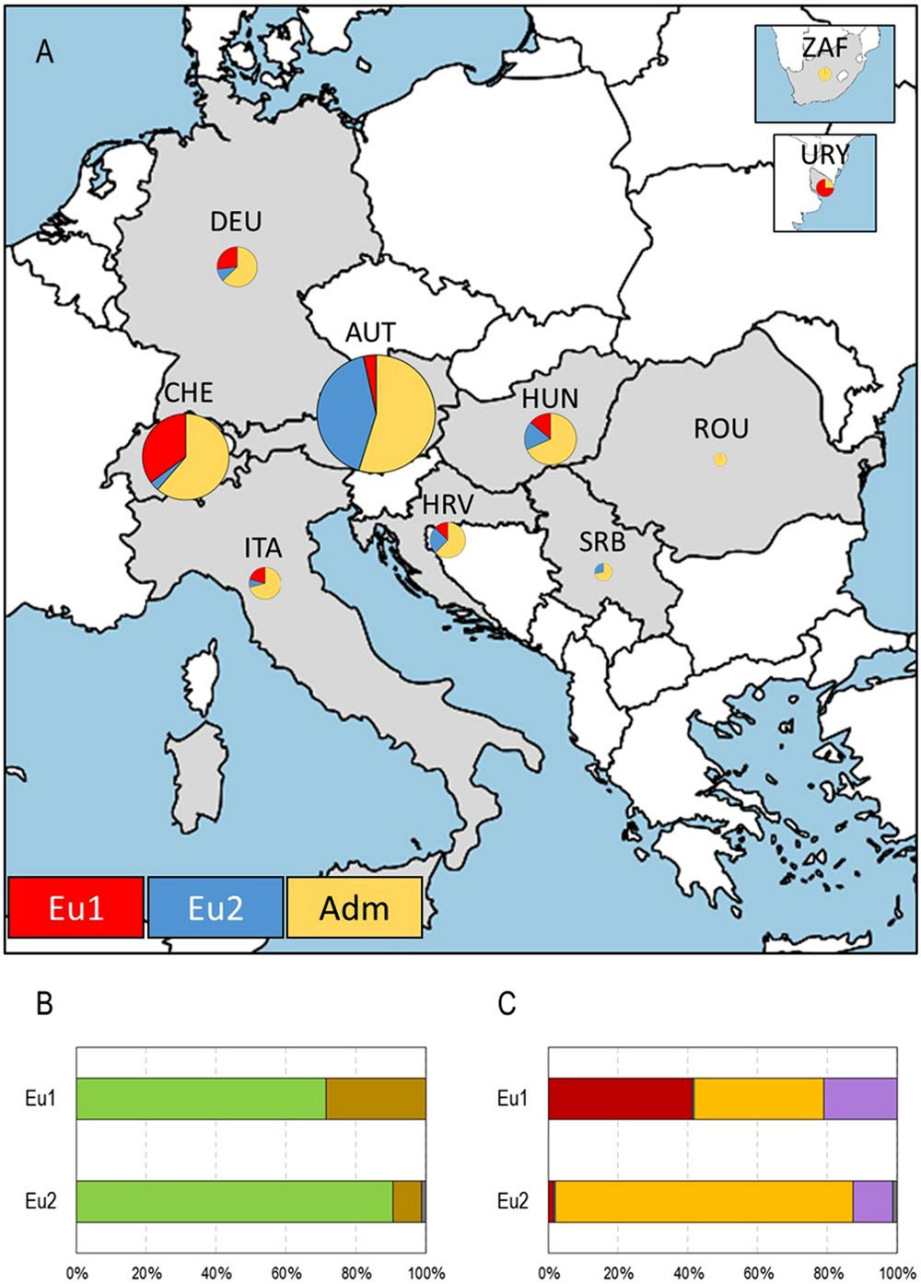
Analysis of the effect of sampling site (**A**), feeding form (**B**) and host plant (**C**) on grape phylloxera structure in Europe. In A, the geographic distribution of the two genetic groups obtained by STRUCTURE (Eu1 and Eu2) in the geographic range analysed is shown (AUT: Austria; CHE: Switzerland; DEU: Germany; HRV: Croatia; HUN: Hungary; ITA: Italy; ROU: Romania; SRB: Serbia; URY: Uruguay; ZAF: South Africa). Every country is represented as a circle, which size is proportional to the number of individuals sampled, and its colour represent the number of MLGs assigned to Eu1 (red), Eu2 (blue) or unassigned (admixed, yellow). In B, the percentage of grape phylloxera samples collected from leaves (green) and roots (brown) are shown for each genetic group (Eu1, Eu2). Samples from unknown origin are shown in grey. In C, the percentage of grape phylloxera samples collected from *Vitis* spp. interspecific hybrid direct-producers (dark red), *Vitis* spp. interspecific resistant grape varieties (light green), *Vitis* spp. interspecific rootstocks (yellow) and *Vitis vinifera* L. cultivars (violet) are shown for each genetic group (Eu1, Eu2). Samples collected of plants whose identity could not be confirmed are shown in grey. Map shown in A was created by using MapChart (https://mapchart.net).

MLGs associated with Eu1 mainly come from samples collected in Switzerland (59 MLGs), Germany (21 MLGs), Uruguay (19 MLGs), Hungary (14 MLGs), Italy (13 MLGs), Croatia (9 MLGs) and Austria (8 MLGs) (Fig. 2A). On the contrary, MLGs clustered in Eu2 were mostly isolated in Austria (96 MLGs), followed by Hungary (18 MLGs), Croatia (17 MLGs), Serbia (9 MLGs), Germany (8 MLGs), Switzerland (6 MLGs) and Italy (5 MLGs). Grape phylloxera samples obtained from Romania (3 samples, corresponding to 3 MLGs) and South Africa (8 samples, corresponding to 2 MLGs) did not cluster to either Eu1 or Eu2. Regarding grape phylloxera feeding forms, MLGs linked to Eu1 were collected from both leaves (105 MLGs) and roots (38 MLGs) (Fig. 2B). MLGs in Eu2 were mainly collected from leaves (142 MLGs), followed by roots (16 MLGs) and one MLG which was found in both leaves and roots (MLG521). Lastly, MLGs associated with Eu1 were obtained from *Vitis* spp. interspecific hybrid direct-producers (57 MLGs), *Vitis* spp. interspecific rootstocks (54 MLGs), *V. vinifera* L. cultivars (31 MLGs) and one *Vitis* spp. interspecific resistant grape variety (1 MLG). On the contrary, MLGs in Eu2 were mostly collected from *Vitis* spp. interspecific rootstocks (125 MLGs) (Fig. 2C). More detailed information can be found in the Supplementary Table S1.

Genetic parameters indicated a similar level of genotypic diversity for Eu1 and Eu2 genetic groups, with values rather similar to those calculated for the whole dataset (Table 2). The clonal origin of all the repeated MLGs found in Eu1 and Eu2 was confirmed through the significant *P*_*sex*_ values (<0.01). The average number of alleles per locus is similar in Eu1 (6.14) and Eu2 (5.71). We detected 10 and 13 private alleles in Eu1 and Eu2, respectively, which were found across all the loci screened. A lower than expected level of heterozygosity was observed in Eu1 and Eu2, suggesting the effect of inbreeding forces on both populations. Lastly, *F*_*IS*_ values were positive for most of the loci analysed in this work, but for *PhyIII_36* in Eu1 and *PhyIII_30* and *DVSSR4* in Eu2.

### Analysis of the factors affecting grape phylloxera population structure in Europe

The three-way ANOVA analysis revealed a major significant effect of sampling site (country) (*F* = 23.50; *p* ≤ 0.05) on grape phylloxera genetic structure, as well as a minor effect of host plant (*F* = 3.49; *p* ≤ 0.05). No significant effect for the feeding behaviour was observed (*F* = 1.09; *p* = 0.29), neither for the two-way or three-way interactions between the factors evaluated. Linear regression modelling results revealed a significant effect (*p* ≤ 0.05) of longitude and latitude of the sampling sites on grape phylloxera population structure. Attending to the standardised beta coefficients given by the model to each variable to compare their relative relevance on the genetic structure obtained, we observed a major effect of longitude (beta = 0.626) over latitude (beta = 0.171).

### Comparative analysis of grape phylloxera MLGs in Europe, its native range and other introduced regions

**Figure 3:**
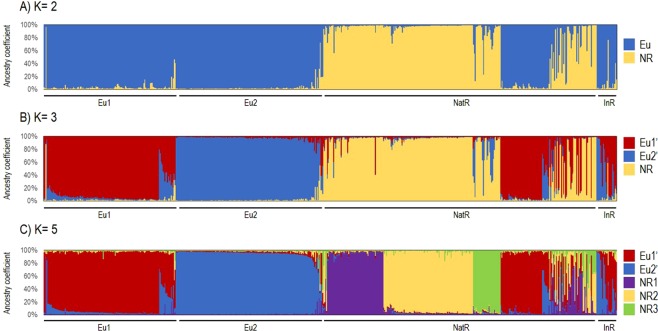
STRUCTURE analysis to elucidate the origin of grape phylloxera in Europe. The population structure of 302 grape phylloxera MLGs from Europe (143 of Eu1 and 159 of Eu2) and 319 MLGs from the native range (NatR) and diverse introduced regions (InR, data from Lund *et al*.^3^) was analysed considered 4 SSR loci. In each plot, every MLG is shown as a vertical line, whose colour(s) indicate ancestry proportions. The three levels of genetic stratification shown at *K* = 2 (**A**), *K* = 3 (**B**) and *K* = 5 (**C**) were determined according to the *ΔK* criteria proposed by Evanno *et al*.^46^. Considering a critical ancestry coefficient of *Q* ≥ 0.80, two genetic groups (Eu and NR, with 376 and 212 MLGs respectively) were obtained at *K* = 2. At *K* = 3, three genetic groups (Eu1’, Eu2’ and NR, with 188, 160 and 195 MLGs, respectively) were observed. At *K* = 5, five genetic groups (Eu1’, Eu2’, NR1, NR2 and NR3, Eu1’ and Eu2’, with 172, 157, 62, 97 and 30MLGs, respectively) were obtained.

To gain insight into the origin of grape phylloxera in Eu1 and Eu2 genetic groups, the genetic information of the 302 MLGs assigned to these groups was combined with that of 319 MLGs from the native range and several introduced regions, obtained from Lund *et al*.^3^. STRUCTURE analysis and the *ΔK* criteria indicated *K* = 2 as the optimal level of structuring, although additional levels of structure were suggested at *K* = 3 and *K* = 5 (Supplementary Fig. S2). At *K* = 2, a clear separation between the MLGs from the native range and Europe was obtained (Fig. 3). Considering a critical *Q*-value of 0.80 for group assignation, group 1 (NR) was formed by 212 MLGs, 211 from the native range of grape phylloxera in the current states of Tennessee (42 MLGs), Missouri (34), Virginia (26), North Carolina (17), New Mexico (17) and Arizona (15) (among others), and one MLG sampled in Germany (assigned to Eu1). Group 2 (Eu) clustered 376 MLGs, mainly from Europe (140 of Eu1 and 156 MLGs of Eu2). The remaining MLGs clustering in this group corresponded to samples isolated from the native range, including, among others, isolates from the states of New York (25 MLGs), Pennsylvania (8 MLGs), Massachusetts (6 MLGs), South Dakota (6 MLGs) and Indiana (5 MLGs), as well as some MLGs from the introduced regions of Austria (7 MLGs), Hungary (5 MLGs), California (4 MLGs), Uruguay (3 MLGs), and Brazil (3 MLGs) retrieved from Lund *et al*.^3^ (Supplementary Table S2). The increase in *K* generated the separation of one of these two main genetic groups in several subgroups (Fig. 3). At *K* = 3, the NR genetic group had no major changes (it clustered 195 MLGs from the native range, and no MLGs from Europe or other introduced regions were found in this genetic group), but the Eu genetic group was divided into two genetic groups (Eu1’ and Eu2’). This division matched with the genetic structuring previously observed when analysing the 774 MLGs from Europe. Eu1’ grouped 188 MLGs, 122 of which were previously assigned to the Eu1 genetic group. It also clustered samples from the states of New York (22 MLGs), South Dakota (10), Pennsylvania (8), Massachusetts (7), Indiana (5), Minnesota (4) and Arkansas (4), as well as from the introduced regions of Uruguay (3 MLGs), Brazil (2) and Hungary (1). Eu2’ grouped 160 MLGs, 151 of which were previously assigned to the Eu2 genetic group. The remaining MLGs corresponded to 1 MLG from Eu1, 2 from the Arizona state, 5 from Austria and 1 from Hungary (the latter corresponding to isolates from Lund *et al*.^3^) (Supplementary Table S2). Eu1’ and Eu2’ genetic groups were well conserved at *K* = 5, but NR split into three genetic subgroups: NR1, NR2 and NR3 (Fig. 3), corresponding largely to major geographic regions of the grape phylloxera native range. NR1 clustered 62 MLGs, mainly from the states of Virginia (18 MLGs), North Carolina (15) and Arkansas (11). NR2 grouped 97 MLGs, mostly from Tennessee (36) and Missouri (26). Lastly, NR3 clustered 30 MLGs, most of them sampled in Arizona (21) and New Mexico (8) (Supplementary Table S2).

**Figure 4:**
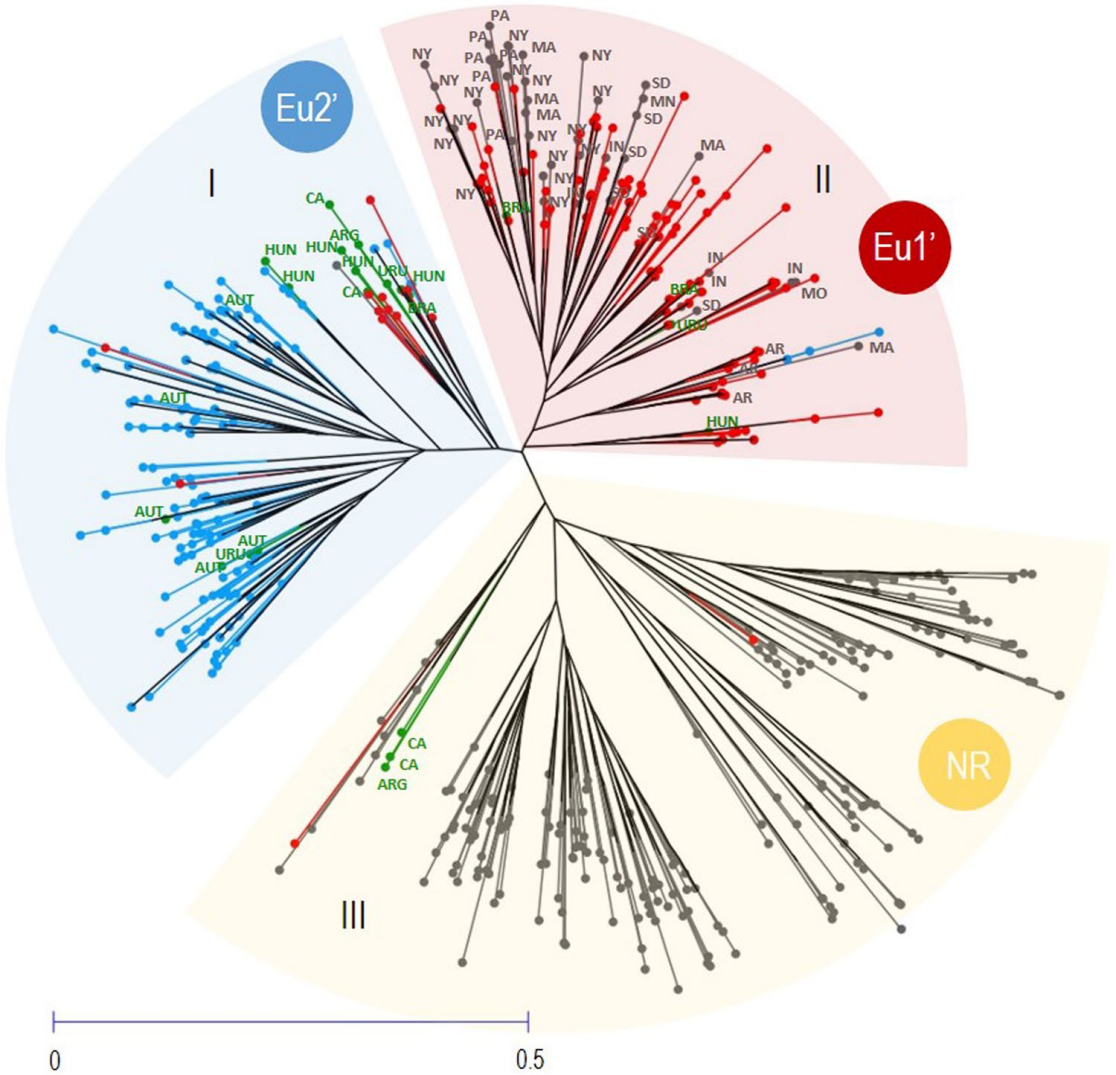
Neighbor-Joining Unweighted (NJUw) dendrogram to elucidate the origin of grape phylloxera in Europe. The NJUw dendrogram was constructed in DARwin based on 4 SSR markers and considering 298 grape phylloxera MLGs (141 of Eu1 and 157 of Eu2) and 277 MLGs from the native and introduced regions (data from Lund *et al*.^3^). Individuals from Eu1, Eu2, the native range and the introduced regions are shown in red, blue, black and green, respectively. The origin of the samples of the introduced regions analysed by Lund *et al*.^3^ are indicated in green (ARG: Argentina; AUT: Austria; BRA: Brazil; CA: California; HUN: Hungary; URU: Uruguay). The dendrogram indicates three main groups (I, II and III) that agree with the three genetic groups observed by STRUCTURE at *K* = 3 (Eu1’, Eu2’ and NR). For simplicity, only the origin of the samples of the native range clustering in the Eu2 or Eu1 groups are indicated in black (AR: Arkansas; IN: Indiana; MA: Massachusetts; MN: Minnesota; MO: Missouri; NY: New York; PA: Pennsylvania; SD: South Dakota).

The neighbor-joining unweighted dendrogram calculated for 575 MLGs (141 previously assigned to Eu1, 157 to Eu2, and 277 from the native range or the introduced regions analysed by Lund *et al*.^3^) showed three distinct groups (Fig. 4), which matched with the distribution of the MLGs obtained by STRUCTURE at *K* = 3 (Fig. 3). Cluster I was mainly composed by MLGs previously assigned to Eu2’ and some of the MLGs retrieved from Lund *et al*.^3^ from the introduced regions of Austria, Hungary, Uruguay, Argentina and California. Cluster II mostly grouped MLGs attributed to Eu1’ and MLGs from several states of the native range, namely New York, Pennsylvania, Massachusetts, Indiana, South Dakota, Minnesota, Missouri and Arkansas, and samples from the introduced regions of Brazil, Hungary and Uruguay. Cluster III included three MLGs from the introduced regions of California and Argentina, and a high number of MLGs from different regions of the US, including states like Arizona, New Mexico, Tennessee and Texas.

## Discussion

Unravelling the origin of grape phylloxera in Europe is critical to resolve previous controversy about its origin. Likewise, it provides relevant information to set efficient viticultural tactics to deal with this pest in the upcoming decades, especially considering issues derived from climate change and new vineyard management practices. In this study, we analysed the genetic diversity and structure in 1173 grape phylloxera individuals through phylogenetic and population structure analyses. Samples were collected considering the latitudinal range comprised between the Rhine district in Germany and the Tuscany in Italy, and the longitudinal range between the municipalities of Nyon (western Switzerland) and Iași (eastern Romania), which represents a quantitative improvement compared to previous works^20,21^.

Our results strongly indicate the presence of at least two independent grape phylloxera introductions from the native range into Europe, which derived into two genetic groups (Eu1 and Eu2). The structuring of grape phylloxera populations into two groups was already suggested by Forneck *et al*.^20^, who found a loose correlation with sampling latitude. Unfortunately, authors did not test the effect of longitude. Here, we also found a significant effect of latitude on the genetic structure, but longitude had a major effect. In this regard, Eu1 is mostly formed by grape phylloxera genotypes that dominate in western regions (Germany, Italy and Switzerland), whereas Eu2 genotypes dominate in eastern areas (Austria, Hungary, Croatia and Serbia). This genetic structure observed from nuclear genomic analyses is in accordance with previous mtDNA phylogenetic analyses that indicate a common origin for grape phylloxera in Germany and France, but independent to those from Hungary^23^. The effect of host plant on the selection of specific grape phylloxera clones has been already suggested^1,24^, especially in the native range^3,25^ as a result of centuries of plant-insect coevolution^4^. Here, we observed a significant effect of host plant on the genetic structure, but it can be the result of the non-random distribution of the different plant hosts in the diverse regions analysed in this work (e.g.: no *Vitis* spp. interspecific rootstocks were sampled in Germany; no *Vitis* spp. interspecific hybrid direct-producers were sampled in Austria). In this line, a fine-scale exploration is needed to separate host plant- and geography-based associations.

Considering the geographic distribution and historical reports, it seems plausible that genotypes of Eu1 derived from the grape phylloxera population introduced in the South of France in the 1860s^8^, which spread northwards and southwards to infest Swiss and Italian vineyards, respectively^10,14^. In addition, our results indicate that this introduction might have been responsible of the outbreaks reported in the west of Germany between 1874 and 1900 too^10^, as no additional stratification between Swiss, German and Italian samples was observed. On the other hand, grape phylloxera genotypes of Eu2 might have directly derived from the grape phylloxera outbreak detected in 1868 in the nurseries of the formerly known as the Imperial and Royal Viticulture Research Station at Klosterneuburg (Austria)^16^, where vine cuttings from different regions (including the USA and France) were received^26^. According to historical records, vine cuttings were distributed as a solution against oidium before grape phylloxera symptoms were evident, easing the dispersal of the insect across the former Austro-Hungarian Empire^10,26^. The independence on the origin of these two outbreaks was already evidenced in the 1870s, as no grape phylloxera injuries were detected when all the plant material received from France was screened at the Klosterneuburg nurseries^27^.

Our analysis confirmed previous findings that indicated a close relationship between European grape phylloxera populations and north-eastern American populations^3,19,23^. Nevertheless, we found this statement to be exclusively true for the Eu1 genetic group, which was likely founded by grape phylloxera populations originating from the North East coast of North America, where *V. riparia* and *V. labrusca* dominate^4^. How grape phylloxera was introduced into France is still controversial, although it is generally accepted it might have been introduced on roots of *V. labrusca* and/or leaf-galled *V. riparia* plants^4^, agreeing with our results. Some historical records suggest that the French outbreak probably arose from the cultivation of infested plants of the hybrid grape cultivar Isabella à grains noirs, introduced from Pennsylvania^28^. Some reports additionally indicate that first grape phylloxera populations could have been introduced to France via United Kingdom, where grape leaf galls were already observed in table grape vines by 1863^11^. The cultivation of Isabella à grains noirs vines (probably corresponding to the cultivar nowadays known as Isabella, *V. labrusca* × *V. vinifera*) was rather common in the South of France due to its pleasant fragrance and raspberry-like flavour^26,28^. Unfortunately, this trend favoured the rapid dissemination of the insect across neighbouring vineyards, and caused the decease of a great number of vines throughout west and central Europe. On the other hand, historical reports indicate that the strains initially introduced into Austria (founder of the Eu2 genetic group) could have been transported within a set of 20 American varieties imported to Klosterneuburg from New Jersey (USA), which were planted in the experimental vineyard to evaluate their fitness against oidium^29^. Nevertheless, we could not place the point of origin of this genetic group, as genotypes in Eu2 clustered only with samples from other introduced regions and not with those of the native range. In this line, it would be of interest to cross our data with a major number of grape phylloxera samples collected in the native range, as a wide insect genetic diversity exists as the result of plant-insect coevolution^4^.

Samples from the introduced regions of Uruguay and South Africa did not form a specific cluster, suggesting that (I) either both regions received infested plant material from Europe, or (II) the introduction came from the same source(s) as those that devastated European vineyards. Accordingly, it has been recently indicated that Uruguayan populations might have resulted from the unintended introduction of infested plant material from Europe^30^.

Our analysis revealed an important role of the first introductions that occurred in the nineteenth century in Europe. Considering historical records, it is likely that the introduced grape phylloxera populations rapidly spread across the continent by the unintended transportation of infested American vine cuttings to fight other disease-causing agents. This human-assisted dispersal counteracted the sessile nature of the insect for most of its life cycle^2^, which presents a low capability of colonizing new vines or vineyards^5^. The lack of natural resistance in *V. vinifera* L. and the absence of natural enemies in European vineyards favoured the fast colonization of the new environment. Genetic diversity parameters indicate that these initial populations were subjected to different selection pressures that lead to the selection of different alleles for local adaptation, generating a series of clones mainly by parthenogenesis, as suggested by significant *P*_*sex*_ values. In fact, parthenogenesis is acknowledged as the preferable mechanism for grape phylloxera reproduction in different introduced regions^21,31–33^. Clonal reproduction is still capable of generating a high level of genetic variation per generation^34^. This reproductive mechanism likely facilitated the long-term persistence of early introduced populations until today, as this strategy favours aphid populations persistence in areas with temperate climate^35^, like European winemaking regions^36^. Unravelling the mechanisms that drove to the generation of site- and host-adapted grape phylloxera populations in Europe will aid to understand why some of the rootstocks generated more than a century ago are still useful to control grape phylloxera effects, as well as to plan effective measures of control (including the breeding of new more resistant rootstocks) to anticipate to new, more aggressive outbreaks.

## Material and Methods

### Grape phylloxera sampling

Leaf and soil-emerged grape phylloxera individuals were collected from *V. vinifera* L. cultivars (such as Cabernet Sauvignon and Chardonnay), *Vitis* spp. interspecific rootstocks (like Kober 5BB and Teleki 5C, from both abandoned and commercial vineyards), *Vitis* spp. interspecific hybrid direct-producers (like Léon Millot and Maréchal Foch), and *Vitis* spp. interspecific resistant grape varieties (also known as PIWIs, like Kosmopolita and Muscaris) in 100 locations from eight European countries (Austria, Croatia, Germany, Hungary, Italy, Romania, Serbia and Switzerland). It corresponded to a latitudinal gradient from 43.0°N to 50.0°N and a longitudinal gradient between 6.2°E and 27.6°E. Samples were collected from 2012 to 2019 (Supplementary Fig. S3). In addition, some leaf-feeding grape phylloxera samples from Uruguay and South Africa were considered to acquire a representation of grape phylloxera from other introduced regions (Table 1). Grape phylloxera feeding form and host plant was annotated for each collected sample, and GPS coordinates (latitude and longitude) were recorded for each sampling site as decimal degrees. Supplementary Table S3 provides more information of the samples collected, and details on most of the samples analysed from Austria, Switzerland and Germany can be found elsewhere^18,19^. Leaf-feeding grape phylloxera individuals were obtained by collecting galled leaves from infested vines, which were stored at −20 °C until further analyses^19^. Root-feeding grape phylloxera individuals were sampled using emergence traps, as detailed in Powell *et al*.^37^. After insect collection, root-feeding grape phylloxera individuals were stored in ethanol at 2 °C for DNA extraction. In total, 1173 individuals (1121 from Europe, 44 from Uruguay and 8 from South Africa) have been analysed in this work.

### DNA isolation and genotyping

Whole genomic DNA was extracted from grape phylloxera individuals as indicated in Forneck *et al*.^19^ and stored at −20 °C for further use. A total of 1173 samples were genotyped with seven fluorescently labelled (6-FAM or HEX) simple sequence repeat (SSR) primers (*Phy_III_55*, *Phy_III_30*, *Phy_III_36*, *Dvit6*, *DV4*, *DV8* and *DVSSR4*). DNA amplification, fragments selection and allele calling were performed as detailed previously^19,38^. In every set of samples, we included six control genotypes with known allele size to keep allele calling consistent between different runs. This analysis revealed the presence of 774 grape phylloxera MLGs.

### Grape phylloxera MLGs from the american native range and introduced regions

In a previous work, Lund *et al*.^3^ explored the population genetic diversity of grape phylloxera in its native range, using samples collected across the USA, as well as some samples obtained from Europe (Austria and Hungary) and South America (Argentina, Brazil, Peru and Uruguay). These samples were genotyped using 32 SSRs, including the SSR markers *Phy_III_55*, *Phy_III_30*, *Phy_III_36* and *Dvit6*. We retrieved the information of these 4 markers to perform a joint analysis between these samples and our genotypes.

### Genetic analyses

#### Population structure analysis of grape phylloxera in Europe

The Bayesian clustering software STRUCTURE v.2.3.4^39^ was used to infer the number of genetic groups and to assign individuals to the inferred populations, using our dataset of 774 MLGs based on 7 SSR markers. The existence of genetic structure was tested in a number of hypothetical genetic groups (*K*) from 1 to 15, using a cycle of 250.000 burn-in steps followed by 500.000 Markov Chain Monte Carlo iterative steps. Ten replications per *K* value were run to assess the consistency of the results, and each one was performed considering an admixture model with uncorrelated allele frequencies. Aware of the uneven sampling for the factors likely affecting grape phylloxera population structure (sampling site, feeding form, host plant), four estimators of the most likely *K* value (median of means (*MedMeaK*), maximum of means (*MaxMeaK*), median of medians (*MedMedK*) and maximum of medians (*MaxMedK*)) were determined using a membership coefficient threshold of 0.5, as proposed by Puechmaille^40^ and implemented in STRUCTURE SELECTOR^41^. Thus, these four statistics were independently calculated using the different group classes obtained for sampling site (Austria, Croatia, Germany, Hungary, Italy, Romania, Serbia, South Africa, Switzerland or Uruguay), feeding form (leaf or root) or host plant (*V. vinifera* L. cultivars, *Vitis* spp. interspecific rootstocks, *Vitis* spp. interspecific hybrid direct-producers or *Vitis* spp. interspecific resistant grape varieties) as co-factors. The statistics of the three scenarios were then compared, and the most likely number of genetic groups was determined as the most repeated *K* value within the 12 calculations. Once the most probable *K* value was set, MLGs were assigned to a genetic group considering an ancestry coefficient *Q* ≥ 0.80; otherwise, a MLG was considered as “admixed”. In parallel, a dissimilarity matrix was calculated between 752 grape phylloxera MLGs (22 were discarded because of the presence of missing data) using the DARwin software package v. 6.0.21^42^ on the basis of 10.000 bootstrap steps, which was further used for a Principal Coordinate Analysis (PCoA). As indicated by Emanuelli *et al*.^43^, a Neighbor-Joining Unweighted (NJUw) dendrogram was constructed for the subset of MLGs clustered in the genetic groups previously determined to support the consistency of STRUCTURE and PCoA clustering methods. The dendrogram was constructed on the basis of a dissimilarity matrix with 10.000 bootstrap steps, and it was drawn considering 1.000 bootstrap replicates. MLGs with missing data were excluded from the analysis. The three outcomes (STRUCTURE, PCoA and NJUw) were used to determine the optimum level of genetic structure of grape phylloxera in Europe.

For each genetic group, we calculated the mean number of alleles per locus, the observed (*H*_*obs*_) and expected (*H*_*e*_) heterozygosities under Hardy-Weinberg expected equilibrium, and *F*_*IS*_ values, which were independently calculated for each locus. The genotypic diversity index (*R*) was estimated as *R* = (MLG-1)/(*N*-1), and the clonal diversity index (*Pd*) as *Pd* = MLGs/*N*, MLG being the number of multilocus genotypes identified, and *N* the number of sampled individuals. All calculations were performed using MLGsim 2.0^44^ with 10.000 simulation steps. In addition, the same software was used to calculate the *P*_*sex*_ statistic to estimate the likelihood that grape phylloxera individuals with the same MLG are products of distinct sexual reproductive events or truly clonal. Thus, a multiple MLG was considered to be a true clone if *P*_*sex*_ < 0.01 (i.e. low probability of being the result of sexual reproduction).

#### Analysis of the factors affecting grape phylloxera population structure in Europe

To evaluate the effect of sampling site (country), feeding form and host plant on the genetic structure of grape phylloxera in Europe, a three-way ANOVA was calculated focusing in the European MLGs with an ancestry coefficient *Q* ≥ 0.80 in any of the identified genetic groups (n = 302). For this analysis, three variables (country, feeding form and host plant) were used as independent variables, defining eight levels for country, two for feeding form and four for host plant, as detailed above. As dependent variable we used the *Q* values given by STRUCTURE to each MLG (*Q*-matrix). Factors were considered to have a significant effect on the *Q*-matrix at *p* ≤ 0.05. To deepen into the effect of geography on the genetic structure, we calculated a linear regression model using the *Q*-matrix as dependent variable and latitude and longitude values of the sampling sites as independent variables. Factors were considered significant if *p* ≤ 0.05. Analyses were performed using SPSS v.24.0 (IBM, Chicago, IL, USA).

#### Population structure analysis of grape phylloxera in Europe and its introduced region

A second population structure analysis was performed with STRUCTURE^39^ to analyse the relationship between the 302 European MLGs clustered in the final genetic groups previously identified and the MLGs reported in Lund *et al*.^3^ sampled in the native range and in diverse introduced regions. In total, 621 MLGs have been included in this analysis. To this aim, we used the procedure described above, but using the information available for four SSR loci (*Phy_III_55*, *Phy_III_30*, *Phy_III_36* and *Dvit6*). The most likely number of genetic groups was assessed by plotting the *ΔK* value of the data over 10 runs, as implemented in STRUCTURE HARVESTER^45^, and defined in Evanno *et al*.^46^. MLGs were assigned to a genetic group if they have a coefficient of ancestry (*Q*) over 0.80; otherwise, they were considered as “admixed”. A dissimilarity matrix was calculated considering genetic data on 4 SSRs for 575 MLGs as previously detailed, discarding 46 MLGs for the presence of missing data. The matrix was then used to construct an NJUw dendrogram with 1.000 bootstrap replicates to get insights into the relationship between the genetic groups identified in our dataset and those previously reported from grape phylloxera native range and different introduced regions^3^.

## Supplementary information

Supplementary information
