# Supplementary material for: Major Outbreaks in the Nineteenth Century Shaped Grape Phylloxera Contemporary Genetic Structure in Europe
Source: Sci Rep. 2019 Nov 26;9:17540. doi: 10.1038/s41598-019-54122-0 (PMC6879566; doi:10.1038/s41598-019-54122-0)
Supplement: Supplementary file 1 — Supplementary information [file 41598_2019_54122_MOESM1_ESM.pdf]

Major Outbreaks in the Nineteenth Century Shaped Grape Phylloxera Contemporary Genetic Structure in Europe

Javier Tello[1,*], Roswitha Mammerler[1], Marko Čajić[1,2], Astrid Forneck[1]

[1] Department of Crop Sciences, Institute of Viticulture and Pomology, University of Natural Resources and Applied Life Sciences Vienna, Konrad Lorenz Str. 24, A-3430 Tulln, Austria

[2] Faculty of Agricultural Sciences, University of Zagreb, Svetošimunska cesta 25, 10000, Zagreb, Croatia

[*] javier.tello@boku.ac.at

List of Supplementary Files

Supplementary Figure S1. Estimates of the number of genetic groups (*K*) in grape phylloxera European populations based on *MedMedK*, *MedMeanK*, *MaxMedK* and *MaxMeanK* statistics obtained from STRUCTURE SELECTOR using (A) sampling site (country), (B) feeding form or (C) host plant as correcting factor. The horizontal red line indicates the most likely number of genetic groups in the population in each case.

Supplementary Figure S2. Delta K plot obtained from STRUCTURE HARVESTER to determine the most likely number of genetic groups present in 302 grape phylloxera MLGs obtained from Europe and 319 MLGs from the native range and diverse introduced regions (data from Lund et al.[3]).

Supplementary Figure S3. Location of the sampling sites of grape phylloxera populations (coloured circles). Note that for the sake of simplicity, sites in South Africa and Uruguay are not shown. Map was generated using Multiplottr 3.0 (http://multiplottr.com).

Supplementary Table S1. SSRs profiles of the 774 multi-locus genotypes (MLGs) identified in this study. For each MLG, we indicate its sampling site (country), grape phylloxera feeding form, host plant and genetic group assigned by STRUCTURE analysis (K=2).

Supplementary Table S2. Genetic group assignation at K=2, 3 and 5 for 302 grape phylloxera MLGs identified in Europe (143 of Eu1 and 159 of Eu2) and 319 MLGs from the native range.

Supplementary Table S3. List of the 1173 grape phylloxera individuals included in this study. For each sample, we indicate the sampling site (country), grape phylloxera feeding form and host plant.

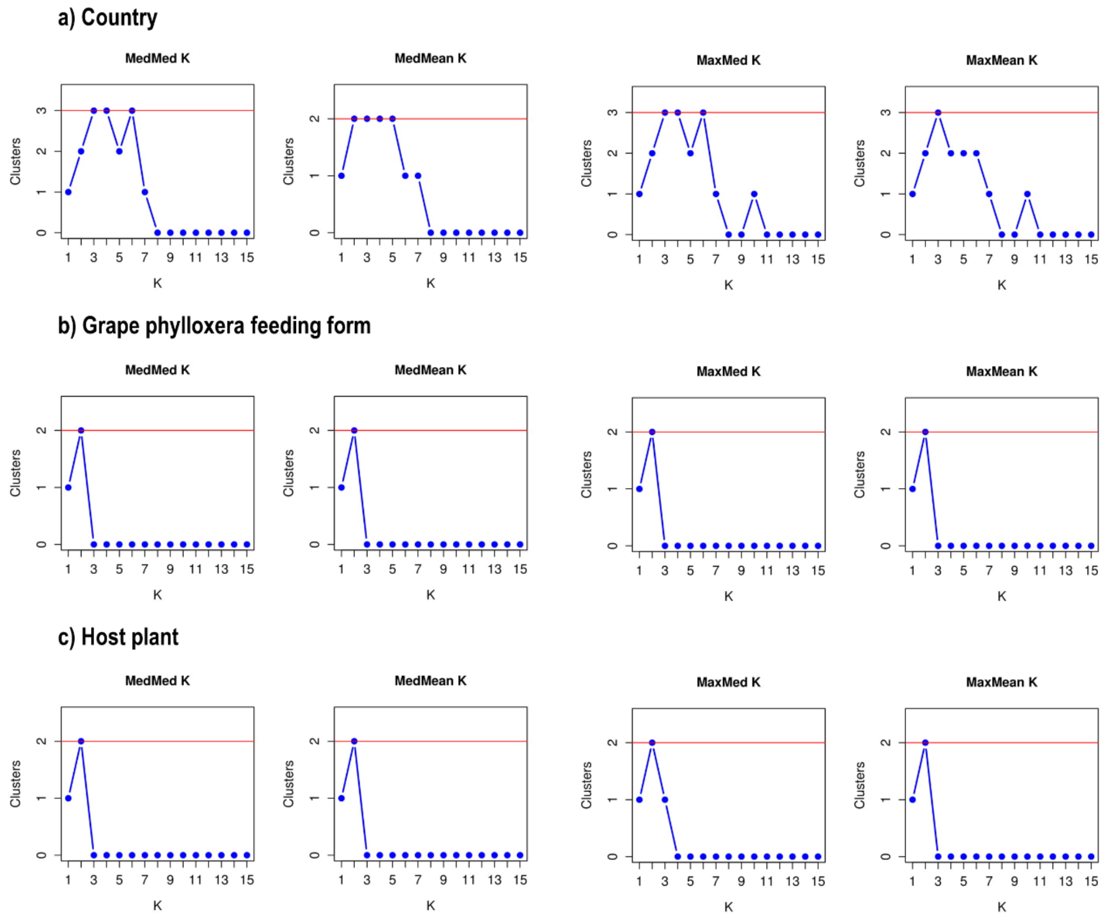

Supplementary Figure S1. Estimates of the number of genetic groups (*K*) in grape phylloxera European populations based on *MedMedK*, *MedMeanK*, *MaxMedK* and *MaxMeanK* statistics obtained from STRUCTURE SELECTOR using (A) sampling site (country), (B) feeding form or (C) host plant as correcting factor. The horizontal red line indicates the most likely number of genetic groups in the population in each case.

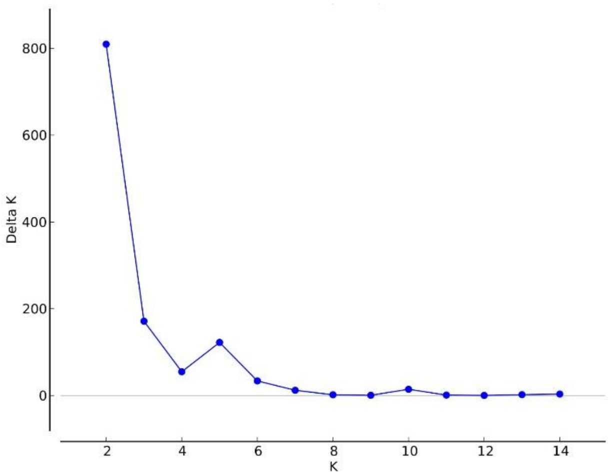

Supplementary Figure S2. Delta K plot obtained from STRUCTURE HARVESTER to determine the most likely number of genetic groups present in 302 grape phylloxera MLGs obtained from Europe and 319 MLGs from the native range and diverse introduced regions (data from Lund et al. [3]).

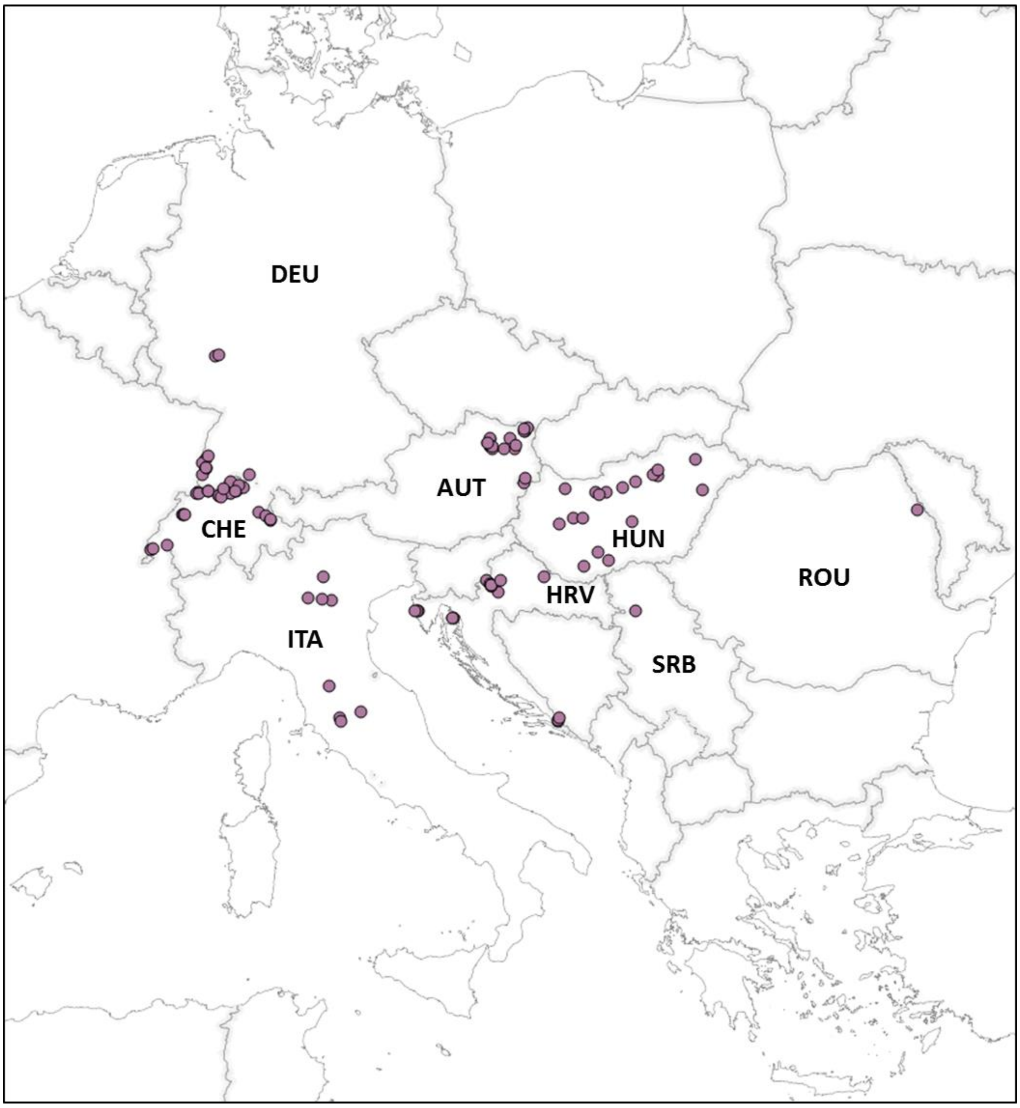

Supplementary Figure S3. Location of the sampling sites of grape phylloxera populations (coloured circles). Note that for the sake of simplicity, sites in South Africa and Uruguay are not shown. Map was generated using Multiplottr 3.0 (http://multiplottr.com).

Supplementary Table S1. SSRs profiles of the 774 multi-locus genotypes (MLGs) identified in this study. For each MLG, we indicate its sampling site (country), grape phylloxera feeding form, host plant and genetic group assigned by STRUCTURE analysis (K=2).

MLG	N	Sample(s) Code[1]	Country[2]	Feeding form	Host plant[3]	PhyIII_55	PhyIII_30	PhyIII_36	DV8	Dvit6	DVSSR4	DV4	Genetic group (K=2)
MLG1	2	AUT001, AUT004	AUT (2)	Leaf (2)	Vvin (2)	130:130	129:132	195:195	145:147	208:208	251:251	210:228	Eu2
MLG2	1	AUT002	AUT (1)	Leaf (1)	Vvin (1)	130:130	129:132	195:195	145:147	208:208	251:251	228:228	Eu2
MLG3	1	AUT003	AUT (1)	Leaf (1)	Vvin (1)	124:124	129:132	195:195	143:145	202:208	245:251	219:222	Admixed
MLG4	1	AUT005	AUT (1)	Leaf (1)	Vvin (1)	124:130	132:132	195:207	147:147	208:208	251:251	210:228	Admixed
MLG5	1	AUT006	AUT (1)	Leaf (1)	RGV (1)	124:124	132:132	195:213	143:147	202:208	251:251	210:222	Admixed
MLG6	1	AUT007	AUT (1)	Leaf (1)	RGV (1)	124:130	129:132	195:207	149:149	196:202	251:251	222:222	Eu1
MLG7	1	AUT008	AUT (1)	Leaf (1)	RGV (1)	121:124	132:132	195:204	143:145	202:202	251:251	222:222	Admixed
MLG8	1	AUT009	AUT (1)	Leaf (1)	RGV (1)	124:127	129:132	195:195	143:145	202:202	241:251	219:219	Admixed
MLG9	2	AUT010, AUT011	AUT (2)	Leaf (2)	Vvin (2)	124:133	129:132	195:213	143:145	205:208	251:251	210:222	Admixed
MLG10	1	AUT012	AUT (1)	Leaf (1)	Vvin (1)	115:124	129:132	195:210	143:147	202:205	251:251	219:222	Admixed
MLG11	2	AUT013, AUT014	AUT (2)	Leaf (2)	Vvin (2)	124:124	129:129	195:195	145:145	202:205	251:251	222:222	Admixed
MLG12	3	AUT015, AUT016, AUT017	AUT (3)	Leaf (3)	RGV (3)	124:130	129:132	195:195	143:145	202:208	251:251	222:222	Admixed
MLG13	1	AUT018	AUT (1)	Leaf (1)	Rts (1)	124:130	132:135	195:195	143:145	208:208	253:253	216:216	Eu2
MLG14	1	AUT019	AUT (1)	Leaf (1)	Rts (1)	124:124	132:132	195:195	143:143	205:208	253:253	216:219	Admixed
MLG15	1	AUT020	AUT (1)	Leaf (1)	Rts (1)	124:130	132:135	195:195	143:143	208:208	251:253	216:216	Eu2
MLG16	18	AUT021, AUT022, AUT023, AUT095, AUT096, AUT100, AUT103,	AUT (18)	Leaf (18)	Rts (18)	130:130	132:135	195:195	145:145	202:208	251:251	222:222	Eu2

MLG	N	Sample(s) Code[1]	Country[2]	Feeding form	Host plant[3]	PhyIII_55	PhyIII_30	PhyIII_36	DV8	Dvit6	DVSSR4	DV4	Genetic group (K=2)
		AUT104, AUT105, AUT106, AUT109, AUT110, AUT111, AUT112, AUT115, AUT119, AUT120, AUT121											
MLG17	1	AUT024	AUT (1)	Leaf (1)	Rts (1)	130:130	132:132	195:198	147:147	199:205	251:251	219:222	Eu2
MLG18	1	AUT025	AUT (1)	Leaf (1)	Rts (1)	124:130	129:129	195:195	145:145	202:208	251:253	219:219	Eu2
MLG19	1	AUT026	AUT (1)	Leaf (1)	Rts (1)	130:130	129:132	195:195	145:145	202:205	251:251	222:228	Eu2
MLG20	1	AUT027	AUT (1)	Leaf (1)	Rts (1)	127:130	129:132	195:195	143:143	202:202	251:251	222:222	Admixed
MLG21	1	AUT028	AUT (1)	Leaf (1)	Rts (1)	130:130	132:132	195:198	145:147	199:205	251:253	219:222	Admixed
MLG22	1	AUT029	AUT (1)	Leaf (1)	Rts (1)	124:127	129:132	195:195	143:143	202:202	251:251	219:222	Admixed
MLG23	1	AUT030	AUT (1)	Leaf (1)	Rts (1)	127:130	129:132	195:198	143:145	202:205	251:251	219:219	Admixed
MLG24	1	AUT031	AUT (1)	Leaf (1)	Rts (1)	130:130	129:129	195:195	143:143	202:202	251:251	222:222	Eu2
MLG25	1	AUT032	AUT (1)	Leaf (1)	Rts (1)	127:130	129:132	195:207	143:145	202:208	251:251	219:219	Admixed
MLG26	2	AUT033, AUT041	AUT (2)	Leaf (2)	Rts (2)	130:130	132:132	195:198	147:147	199:205	251:253	219:222	Admixed
MLG27	1	AUT034	AUT (1)	Leaf (1)	Rts (1)	130:130	129:132	198:198	147:147	199:205	251:253	219:222	Eu2
MLG28	3	AUT035, AUT038, AUT050	AUT (3)	Leaf (3)	Rts (3)	124:124	129:132	195:195	145:145	202:205	245:251	222:225	Admixed
MLG29	1	AUT036	AUT (1)	Leaf (1)	Rts (1)	130:130	132:132	195:195	143:147	202:205	251:251	219:222	Admixed
MLG30	1	AUT037	AUT (1)	Leaf (1)	Rts (1)	124:124	129:132	195:195	145:145	202:205	251:253	222:225	Admixed
MLG31	1	AUT039	AUT (1)	Leaf (1)	Rts (1)	127:130	132:135	195:195	143:143	202:202	251:251	222:228	Eu2
MLG32	1	AUT040	AUT (1)	Leaf (1)	Rts (1)	127:130	129:132	195:195	145:145	202:205	251:251	222:228	Admixed
MLG33	1	AUT042	AUT (1)	Leaf (1)	Rts (1)	130:130	129:129	195:195	145:147	202:205	245:251	222:228	Eu2

MLG	N	Sample(s) Code[1]	Country[2]	Feeding form	Host plant[3]	PhyIII_55	PhyIII_30	PhyIII_36	DV8	Dvit6	DVSSR4	DV4	Genetic group (K=2)
MLG34	1	AUT043	AUT (1)	Leaf (1)	Rts (1)	124:127	132:135	195:195	143:143	202:202	251:251	222:228	Admixed
MLG35	1	AUT044	AUT (1)	Leaf (1)	Rts (1)	130:130	129:129	195:195	145:145	202:208	245:255	216:219	Eu2
MLG36	1	AUT045	AUT (1)	Leaf (1)	Rts (1)	133:133	129:132	195:195	145:147	202:208	251:253	219:222	Eu2
MLG37	1	AUT046	AUT (1)	Leaf (1)	Rts (1)	124:124	129:132	195:195	145:145	202:208	245:251	222:225	Admixed
MLG38	1	AUT047	AUT (1)	Leaf (1)	Rts (1)	130:130	129:132	195:195	145:147	202:205	251:251	219:228	Eu2
MLG39	1	AUT048	AUT (1)	Leaf (1)	Rts (1)	130:130	132:135	195:198	147:147	199:205	251:253	219:222	Eu2
MLG40	1	AUT049	AUT (1)	Leaf (1)	Rts (1)	124:130	129:132	195:195	143:145	202:202	253:253	216:216	Admixed
MLG41	1	AUT051	AUT (1)	Leaf (1)	Rts (1)	130:130	132:132	195:198	147:147	199:205	251:253	216:219	Eu2
MLG42	1	AUT052	AUT (1)	Leaf (1)	Rts (1)	127:130	129:132	195:195	143:145	202:202	251:253	219:222	Admixed
MLG43	1	AUT053	AUT (1)	Leaf (1)	Rts (1)	127:127	129:132	195:207	145:147	202:205	251:251	216:222	Admixed
MLG44	8	AUT054, AUT055, AUT058, AUT062, AUT070, AUT073, AUT074, AUT075	AUT (8)	Leaf (8)	Rts (8)	121:124	132:135	195:195	145:147	202:208	251:253	210:216	Admixed
MLG45	3	AUT056, AUT056, AUT059	AUT (3)	Leaf (3)	Rts (3)	121:124	132:135	195:195	145:147	202:208	251:253	216:216	Eu2
MLG46	4	AUT060, AUT077, AUT078, AUT080	AUT (4)	Leaf (4)	Rts (4)	127:127	132:132	195:195	145:147	202:202	241:253	210:216	Admixed
MLG47	1	AUT061	AUT (1)	Leaf (1)	Rts (1)	127:127	132:132	195:195	145:147	202:202	253:253	210:216	Admixed
MLG48	1	AUT063	AUT (1)	Leaf (1)	Rts (1)	121:124	132:132	195:195	145:147	202:208	251:253	210:216	Admixed
MLG49	2	AUT064, AUT068	AUT (2)	Leaf (2)	Rts (2)	127:130	132:132	195:195	145:147	202:208	251:251	219:222	Admixed

MLG	N	Sample(s) Code[1]	Country[2]	Feeding form	Host plant[3]	PhyIII_55	PhyIII_30	PhyIII_36	DV8	Dvit6	DVSSR4	DV4	Genetic group (K=2)
MLG50	3	AUT065, AUT066, AUT069	AUT (3)	Leaf (3)	Rts (3)	124:127	132:132	195:198	145:147	202:208	251:251	219:222	Admixed
MLG51	1	AUT067	AUT (1)	Leaf (1)	Rts (1)	130:130	132:132	195:195	145:147	202:208	251:251	219:222	Admixed
MLG52	1	AUT071	AUT (1)	Leaf (1)	Rts (1)	121:124	129:132	195:195	145:147	202:205	251:253	210:216	Admixed
MLG53	1	AUT072	AUT (1)	Leaf (1)	Rts (1)	124:127	135:135	195:195	145:147	202:202	241:251	210:216	Admixed
MLG54	2	AUT076, AUT079	AUT (2)	Leaf (2)	Rts (2)	127:127	132:132	195:195	145:147	202:202	241:253	216:216	Admixed
MLG55	1	AUT081	AUT (1)	Leaf (1)	Rts (1)	127:130	132:132	195:195	145:147	202:202	241:253	216:216	Admixed
MLG56	1	AUT082	AUT (1)	Leaf (1)	Rts (1)	124:130	129:132	195:195	145:145	202:202	251:251	222:228	Eu2
MLG57	1	AUT083	AUT (1)	Leaf (1)	Rts (1)	124:130	132:132	198:198	143:143	208:208	251:253	219:219	Admixed
MLG58	5	AUT084, AUT085, AUT086, AUT087, AUT090	AUT (5)	Leaf (5)	Rts (5)	124:130	132:135	198:198	143:143	208:208	251:253	219:219	Eu2
MLG59	1	AUT088	AUT (1)	Leaf (1)	Rts (1)	124:130	129:129	195:195	145:145	205:208	251:251	219:219	Eu2
MLG60	1	AUT089	AUT (1)	Leaf (1)	Rts (1)	124:124	129:129	195:195	143:145	205:205	251:251	219:222	Admixed
MLG61	1	AUT091	AUT (1)	Leaf (1)	Rts (1)	124:130	132:135	198:198	143:143	208:208	251:251	219:219	Eu2
MLG62	1	AUT092	AUT (1)	Leaf (1)	Rts (1)	124:127	132:135	195:195	143:143	202:205	251:251	210:219	Admixed
MLG63	1	AUT093	AUT (1)	Leaf (1)	Rts (1)	130:133	129:132	195:195	143:145	202:205	251:253	219:222	Admixed
MLG64	1	AUT094	AUT (1)	Leaf (1)	Rts (1)	124:127	129:135	195:195	143:145	202:208	251:251	219:219	Eu2
MLG65	1	AUT097	AUT (1)	Leaf (1)	Rts (1)	130:130	132:135	195:195	145:145	205:205	251:251	222:222	Admixed
MLG66	6	AUT098, AUT102, AUT107, AUT113, AUT116, AUT117	AUT (6)	Leaf (6)	Rts (6)	124:133	132:141	195:195	143:143	205:205	245:251	222:222	Admixed
MLG67	1	AUT099	AUT (1)	Leaf (1)	Rts (1)	124:124	132:132	195:195	145:145	196:196	245:253	222:222	Eu1

MLG	N	Sample(s) Code[1]	Country[2]	Feeding form	Host plant[3]	PhyIII_55	PhyIII_30	PhyIII_36	DV8	Dvit6	DVSSR4	DV4	Genetic group (K=2)
MLG68	1	AUT101	AUT (1)	Leaf (1)	Rts (1)	130:130	132:135	195:195	145:145	202:208	249:249	222:222	Admixed
MLG69	1	AUT108	AUT (1)	Leaf (1)	Rts (1)	127:130	129:141	195:195	145:147	196:202	245:245	210:222	Admixed
MLG70	1	AUT114	AUT (1)	Leaf (1)	Rts (1)	127:130	132:135	195:195	145:145	202:208	253:253	219:222	Admixed
MLG71	1	AUT118	AUT (1)	Leaf (1)	Rts (1)	124:124	132:135	195:195	145:145	202:208	251:251	222:222	Admixed
MLG72	1	AUT122	AUT (1)	Leaf (1)	Rts (1)	130:130	129:132	195:195	145:145	208:208	251:253	219:222	Eu2
MLG73	3	AUT123, AUT125, AUT128	AUT (3)	Leaf (3)	Rts (3)	130:130	129:132	183:195	145:145	208:208	251:253	219:222	Eu2
MLG74	2	AUT124, AUT127	AUT (2)	Leaf (2)	Rts (2)	121:130	129:132	183:195	145:145	208:208	251:253	219:222	Eu2
MLG75	12	AUT126, AUT130, AUT131, AUT132, AUT133, AUT134, AUT135, AUT136, AUT137, AUT138, AUT139, AUT140	AUT (12)	Leaf (12)	Rts (12)	130:130	129:132	183:195	145:145	208:208	251:253	204:219	Eu2
MLG76	1	AUT129	AUT (1)	Leaf (1)	Rts (1)	130:130	129:132	195:195	145:145	208:208	251:253	204:219	Eu2
MLG77	3	AUT141, AUT143, AUT144	AUT (3)	Leaf (3)	Rts (3)	127:130	132:132	195:195	145:145	208:208	251:251	222:222	Admixed
MLG78	1	AUT142	AUT (1)	Leaf (1)	Rts (1)	127:130	132:132	195:195	145:145	208:208	251:251	207:222	Admixed
MLG79	2	AUT145, AUT146	AUT (2)	Leaf (2)	Rts (2)	124:133	132:141	195:198	145:147	202:205	251:253	216:216	Admixed
MLG80	1	AUT147	AUT (1)	Leaf (1)	Rts (1)	124:130	132:135	195:207	143:145	202:208	241:251	222:222	Admixed
MLG81	1	AUT148	AUT (1)	Leaf (1)	Rts (1)	127:133	129:141	183:195	143:145	202:205	251:253	216:222	Eu2
MLG82	1	AUT149	AUT (1)	Leaf (1)	Rts (1)	124:133	129:132	195:198	145:147	202:205	251:253	216:216	Eu2

MLG	N	Sample(s) Code[1]	Country[2]	Feeding form	Host plant[3]	PhyIII_55	PhyIII_30	PhyIII_36	DV8	Dvit6	DVSSR4	DV4	Genetic group (K=2)
MLG83	1	AUT150	AUT (1)	Leaf (1)	Rts (1)	127:133	129:129	195:195	143:145	202:202	251:251	216:222	Eu2
MLG84	1	AUT151	AUT (1)	Leaf (1)	Rts (1)	121:127	129:135	195:213	143:145	205:208	253:253	216:216	Admixed
MLG85	1	AUT152	AUT (1)	Leaf (1)	Rts (1)	130:130	132:141	195:195	147:147	202:202	251:253	216:216	Eu2
MLG86	1	AUT153	AUT (1)	Leaf (1)	Rts (1)	121:133	129:141	195:195	143:145	202:205	251:253	216:216	Eu2
MLG87	5	AUT154, AUT155, AUT156, AUT157, AUT158	AUT (5)	Leaf (5)	Rts (5)	130:130	132:141	195:195	143:143	202:202	251:251	222:222	Admixed
MLG88	2	AUT159, AUT165	AUT (2)	Leaf (2)	Rts (2)	124:130	132:132	195:195	143:143	205:205	241:241	216:216	Eu1
MLG89	4	AUT160, AUT161, AUT163, AUT164	AUT (4)	Leaf (4)	Rts (4)	127:130	129:132	195:204	143:143	202:202	241:253	216:219	Admixed
MLG90	1	AUT162	AUT (1)	Leaf (1)	Rts (1)	127:130	129:132	195:204	143:147	202:205	251:253	216:219	Admixed
MLG91	1	AUT166	AUT (1)	Leaf (1)	Rts (1)	121:124	135:135	195:213	145:145	208:208	253:253	216:216	Eu2
MLG92	1	AUT167	AUT (1)	Leaf (1)	Rts (1)	115:130	132:135	195:213	143:145	205:208	251:253	210:216	Admixed
MLG93	1	AUT168	AUT (1)	Leaf (1)	Rts (1)	127:130	129:135	195:198	145:147	202:202	251:253	210:216	Eu2
MLG94	1	AUT169	AUT (1)	Leaf (1)	Rts (1)	130:133	129:132	195:204	145:145	202:202	251:251	219:219	Eu2
MLG95	1	AUT170	AUT (1)	Leaf (1)	Rts (1)	121:127	135:141	195:213	143:145	208:208	241:251	222:222	Admixed
MLG96	1	AUT171	AUT (1)	Leaf (1)	Rts (1)	127:127	129:135	195:198	145:147	202:202	251:253	216:216	Eu2
MLG97	5	AUT172, AUT173, AUT174, AUT175, AUT176	AUT (5)	Leaf (5)	Rts (5)	121:127	132:132	195:195	143:147	208:208	251:251	216:219	Admixed
MLG98	2	AUT177, AUT179	AUT (2)	Leaf (2)	Rts (2)	121:121	132:135	195:195	143:145	208:208	251:253	216:216	Eu2
MLG99	1	AUT178	AUT (1)	Leaf (1)	Rts (1)	136:136	132:135	195:195	143:145	208:208	251:253	216:216	Eu2
MLG100	1	AUT180	AUT (1)	Leaf (1)	Rts (1)	121:121	132:135	195:195	143:145	208:208	251:253	210:216	Admixed

MLG	N	Sample(s) Code[1]	Country[2]	Feeding form	Host plant[3]	PhyIII_55	PhyIII_30	PhyIII_36	DV8	Dvit6	DVSSR4	DV4	Genetic group (K=2)
MLG101	1	AUT181	AUT (1)	Leaf (1)	Rts (1)	121:121	132:135	195:195	143:143	208:208	251:253	216:216	Eu2
MLG102	1	AUT182	AUT (1)	Leaf (1)	Rts (1)	127:130	120:132	195:195	143:145	202:202	241:251	219:222	Admixed
MLG103	1	AUT183	AUT (1)	Leaf (1)	Rts (1)	127:130	120:132	183:201	143:145	202:202	241:251	219:222	Admixed
MLG104	3	AUT184, AUT186, AUT188	AUT (3)	Leaf (3)	Rts (3)	115:130	132:135	183:195	145:145	202:205	251:251	210:222	Admixed
MLG105	2	AUT185, AUT189	AUT (2)	Leaf (2)	Rts (2)	124:127	129:135	195:213	143:145	205:208	251:253	210:228	Admixed
MLG106	1	AUT187	AUT (1)	Leaf (1)	Rts (1)	130:130	120:132	183:195	145:147	205:208	245:251	222:222	Admixed
MLG107	1	AUT190	AUT (1)	Leaf (1)	Rts (1)	136:136	132:135	195:195	145:145	202:205	251:251	210:222	Admixed
MLG108	6	AUT191, AUT192, AUT193, AUT194, AUT195, AUT196	AUT (6)	Leaf (6)	Rts (6)	130:130	129:135	195:195	143:147	202:202	251:251	216:219	Eu2
MLG109	1	AUT197	AUT (1)	Leaf (1)	Rts (1)	130:130	123:132	195:204	143:145	205:205	251:251	219:222	Admixed
MLG110	1	AUT198	AUT (1)	Leaf (1)	Rts (1)	121:124	132:135	195:204	143:145	205:205	251:251	216:219	Admixed
MLG111	5	AUT199, AUT200, AUT201, AUT202, AUT203	AUT (5)	Leaf (5)	Rts (5)	121:127	132:135	195:204	143:145	205:205	251:251	216:219	Admixed
MLG112	2	AUT204, AUT205	AUT (2)	Leaf (2)	Rts (2)	127:130	129:129	195:195	143:145	205:208	245:251	219:222	Admixed
MLG113	5	AUT206, AUT208, AUT209, AUT210, CHE206	AUT (4), CHE (1)	Leaf (4), Root (1)	HDP (1), Rts (4)	127:130	129:132	195:195	145:145	202:202	241:251	222:222	Admixed
MLG114	1	AUT207	AUT (1)	Leaf (1)	Rts (1)	124:133	129:132	195:198	143:145	208:208	251:253	216:219	Eu2
MLG115	1	AUT211	AUT (1)	Leaf (1)	Rts (1)	130:130	129:132	195:207	145:145	205:208	241:251	222:222	Admixed

MLG	N	Sample(s) Code[1]	Country[2]	Feeding form	Host plant[3]	PhyIII_55	PhyIII_30	PhyIII_36	DV8	Dvit6	DVSSR4	DV4	Genetic group (K=2)
MLG116	4	AUT212, AUT213, AUT215, AUT2016	AUT (4)	Leaf (4)	Rts (4)	127:130	129:132	198:207	145:145	202:208	241:241	222:222	Admixed
MLG117	1	AUT214	AUT (1)	Leaf (1)	Rts (1)	127:130	129:132	195:195	145:145	205:208	251:253	219:222	Admixed
MLG118	1	AUT217	AUT (1)	Leaf (1)	Rts (1)	127:130	129:132	198:207	145:145	202:202	241:241	222:222	Admixed
MLG119	1	AUT218	AUT (1)	Leaf (1)	Rts (1)	130:130	129:129	195:195	145:145	205:208	251:251	219:222	Eu2
MLG120	1	AUT219	AUT (1)	Leaf (1)	Rts (1)	127:130	132:132	183:195	147:147	202:208	241:251	207:222	Admixed
MLG121	3	AUT220, AUT221, AUT222	AUT (3)	Leaf (3)	Rts (3)	127:130	132:132	195:195	147:147	202:208	241:251	222:222	Admixed
MLG122	3	AUT223, AUT225, AUT226	AUT (3)	Leaf (3)	Rts (3)	130:130	129:132	195:195	143:143	202:208	251:251	222:222	Eu2
MLG123	1	AUT224	AUT (1)	Leaf (1)	Rts (1)	130:130	132:132	195:207	145:145	202:208	241:251	222:222	Admixed
MLG124	2	AUT227, AUT229	AUT (2)	Leaf (2)	Rts (2)	130:130	129:132	183:195	143:143	202:208	251:251	222:222	Eu2
MLG125	1	AUT228	AUT (1)	Leaf (1)	Rts (1)	121:130	129:132	183:195	143:143	202:208	251:251	222:222	Eu2
MLG126	4	AUT230, AUT231, AUT232, AUT236	AUT (4)	Leaf (4)	Rts (4)	130:130	129:132	183:195	145:145	202:208	251:251	222:222	Eu2
MLG127	1	AUT233	AUT (1)	Leaf (1)	Rts (1)	127:130	129:141	183:195	143:145	202:202	251:253	216:219	Eu2
MLG128	1	AUT234	AUT (1)	Leaf (1)	Rts (1)	127:130	129:132	195:195	145:145	205:208	245:251	219:222	Admixed
MLG129	1	AUT235	AUT (1)	Leaf (1)	Rts (1)	130:130	129:132	195:207	145:147	202:208	251:251	222:222	Admixed
MLG130	1	AUT237	AUT (1)	Leaf (1)	Rts (1)	130:130	129:129	183:195	145:145	202:208	251:251	222:222	Eu2
MLG131	14	AUT238, AUT239, AUT242, AUT243, AUT244, AUT245,	AUT (14)	Leaf (14)	Rts (14)	127:130	129:132	195:195	143:147	202:202	251:251	222:222	Admixed

MLG	N	Sample(s) Code[1]	Country[2]	Feeding form	Host plant[3]	PhyIII_55	PhyIII_30	PhyIII_36	DV8	Dvit6	DVSSR4	DV4	Genetic group (K=2)
		AUT246, AUT247, AUT248, AUT249, AUT250, AUT251, AUT252, AUT253											
MLG132	1	AUT240	AUT (1)	Leaf (1)	Rts (1)	127:130	129:129	195:210	143:147	202:202	251:251	222:222	Admixed
MLG133	1	AUT241	AUT (1)	Leaf (1)	Rts (1)	127:130	129:132	183:195	143:147	202:202	251:251	222:222	Eu2
MLG134	1	AUT254	AUT (1)	Leaf (1)	Rts (1)	121:124	129:141	195:213	143:145	208:208	251:251	216:228	Eu2
MLG135	3	AUT255, AUT256, AUT257	AUT (3)	Leaf (3)	Rts (3)	121:124	129:132	195:195	145:145	208:208	251:253	216:222	Admixed
MLG136	2	AUT258, AUT259	AUT (2)	Leaf (2)	Rts (2)	121:124	129:141	195:213	143:145	208:208	251:253	216:228	Eu2
MLG137	1	AUT260	AUT (1)	Leaf (1)	Rts (1)	115:127	129:132	195:195	143:145	202:205	251:251	216:222	Admixed
MLG138	1	AUT261	AUT (1)	Leaf (1)	Rts (1)	124:130	129:132	195:195	143:145	202:208	251:251	204:207	Eu2
MLG139	2	AUT262, AUT263	AUT (2)	Leaf (2)	Rts (2)	124:130	129:141	195:195	143:145	202:208	251:251	222:222	Eu2
MLG140	3	AUT264, AUT265, AUT268	AUT (3)	Leaf (3)	Rts (3)	121:130	132:132	195:195	145:145	202:205	241:253	222:222	Admixed
MLG141	1	AUT266	AUT (1)	Leaf (1)	Rts (1)	121:130	123:132	183:195	147:147	202:208	251:253	222:222	Eu2
MLG142	1	AUT267	AUT (1)	Leaf (1)	Rts (1)	130:130	123:132	183:195	147:147	202:208	251:253	222:222	Eu2
MLG143	1	AUT269	AUT (1)	Leaf (1)	Rts (1)	121:130	123:132	183:195	145:145	202:205	241:253	207:222	Admixed
MLG144	6	AUT270, AUT271, AUT272, AUT273, AUT276, AUT277	AUT (6)	Leaf (6)	Rts (6)	124:130	132:135	195:195	143:147	202:202	251:253	216:222	Eu2

MLG	N	Sample(s) Code[1]	Country[2]	Feeding form	Host plant[3]	PhyIII_55	PhyIII_30	PhyIII_36	DV8	Dvit6	DVSSR4	DV4	Genetic group (K=2)
MLG145	1	AUT274	AUT (1)	Leaf (1)	Rts (1)	124:127	129:135	195:195	145:145	202:208	251:253	216:222	Eu2
MLG146	1	AUT275	AUT (1)	Leaf (1)	Rts (1)	124:130	135:135	195:195	143:147	202:202	251:253	216:222	Eu2
MLG147	1	AUT278	AUT (1)	Leaf (1)	Rts (1)	124:127	129:135	195:195	143:143	202:208	251:253	219:222	Eu2
MLG148	2	AUT279, AUT282	AUT (2)	Leaf (2)	Rts (2)	127:130	132:132	195:195	145:145	205:208	251:253	213:219	Admixed
MLG149	1	AUT280	AUT (1)	Leaf (1)	Rts (1)	127:130	135:135	195:195	145:145	202:202	251:251	222:228	Eu2
MLG150	1	AUT281	AUT (1)	Leaf (1)	Rts (1)	127:130	135:135	183:195	145:145	202:202	251:251	222:228	Eu2
MLG151	1	AUT283	AUT (1)	Leaf (1)	Rts (1)	124:127	123:135	195:195	145:145	202:202	251:251	222:222	Admixed
MLG152	3	AUT284, AUT285, AUT286	AUT (3)	Leaf (3)	Rts (3)	127:130	129:132	195:195	143:143	205:205	251:251	210:222	Admixed
MLG153	2	AUT287, AUT288	AUT (2)	Leaf (2)	Rts (2)	124:127	129:132	195:204	145:147	202:205	251:253	222:222	Admixed
MLG154	1	AUT289	AUT (1)	Leaf (1)	Rts (1)	127:130	129:132	195:207	143:143	205:205	251:251	210:222	Eu1
MLG155	2	AUT290, AUT293	AUT (2)	Leaf (2)	Rts (2)	124:130	129:132	195:195	145:145	202:202	251:251	222:222	Admixed
MLG156	1	AUT291	AUT (1)	Leaf (1)	Rts (1)	127:127	132:132	195:195	143:147	202:205	251:251	219:225	Admixed
MLG157	1	AUT292	AUT (1)	Leaf (1)	Rts (1)	124:130	129:132	195:195	145:145	202:202	251:251	207:222	Admixed
MLG158	1	AUT294	AUT (1)	Leaf (1)	Rts (1)	124:130	129:132	183:195	145:145	202:202	251:251	207:222	Eu2
MLG159	1	AUT295	AUT (1)	Leaf (1)	Rts (1)	127:130	129:132	195:195	143:145	205:208	251:253	222:222	Admixed
MLG160	1	AUT296	AUT (1)	Leaf (1)	Rts (1)	124:130	129:132	195:204	145:147	208:208	251:253	213:216	Eu2
MLG161	2	AUT297, AUT298	AUT (2)	Leaf (2)	Rts (2)	127:130	129:135	195:198	145:147	202:208	253:253	222:222	Eu2
MLG162	1	AUT299	AUT (1)	Leaf (1)	Rts (1)	124:127	129:132	195:195	145:145	202:208	253:253	216:222	Admixed
MLG163	1	AUT300	AUT (1)	Leaf (1)	Rts (1)	127:130	129:132	195:198	145:147	202:208	253:253	222:222	Eu2
MLG164	1	AUT301	AUT (1)	Leaf (1)	Rts (1)	121:127	126:132	195:195	145:145	202:205	251:251	216:222	Admixed
MLG165	3	AUT302, AUT304, AUT307	AUT (3)	Leaf (3)	Rts (3)	127:130	129:141	195:195	143:147	202:205	251:253	216:222	Admixed
MLG166	1	AUT303	AUT (1)	Leaf (1)	Rts (1)	124:124	132:135	195:195	143:147	202:208	251:251	222:228	Eu2

MLG	N	Sample(s) Code[1]	Country[2]	Feeding form	Host plant[3]	PhyIII_55	PhyIII_30	PhyIII_36	DV8	Dvit6	DVSSR4	DV4	Genetic group (K=2)
MLG167	1	AUT305	AUT (1)	Leaf (1)	Rts (1)	127:127	132:132	195:195	145:145	202:202	251:253	216:222	Admixed
MLG168	1	AUT306	AUT (1)	Leaf (1)	Rts (1)	127:130	129:141	183:195	143:147	202:205	251:253	216:222	Admixed
MLG169	1	AUT308	AUT (1)	Leaf (1)	Rts (1)	130:130	129:132	195:195	143:143	202:202	251:253	213:222	Admixed
MLG170	4	AUT309, AUT310, AUT311, AUT312	AUT (4)	Leaf (4)	Rts (4)	127:130	129:135	195:204	143:145	202:205	251:251	219:222	Eu2
MLG171	2	AUT313, AUT314	AUT (2)	Leaf (2)	Rts (2)	130:130	129:135	195:195	143:145	202:208	251:251	210:228	Eu2
MLG172	1	AUT315	AUT (1)	Leaf (1)	Rts (1)	127:127	129:135	195:195	147:147	202:208	241:251	210:216	Eu2
MLG173	1	AUT316	AUT (1)	Leaf (1)	Rts (1)	127:133	129:132	195:198	143:147	202:208	251:253	216:228	Eu2
MLG174	1	AUT317	AUT (1)	Leaf (1)	Rts (1)	127:130	129:135	195:195	143:143	208:208	251:251	210:222	Eu2
MLG175	1	AUT318	AUT (1)	Leaf (1)	Rts (1)	121:127	129:135	195:195	145:147	208:208	251:251	216:216	Eu2
MLG176	2	AUT319, AUT321	AUT (2)	Leaf (2)	Rts (2)	121:127	132:135	195:195	145:147	208:208	251:251	216:216	Eu2
MLG177	2	AUT320, AUT327	AUT (2)	Leaf (2)	Rts (2)	130:130	132:135	195:198	143:147	208:208	241:251	216:228	Eu2
MLG178	1	AUT322	AUT (1)	Leaf (1)	Rts (1)	124:133	129:132	195:195	145:147	202:208	251:251	216:222	Eu2
MLG179	2	AUT323, AUT326	AUT (2)	Leaf (2)	Rts (2)	124:133	129:132	195:198	145:147	202:208	251:251	216:222	Eu2
MLG180	1	AUT324	AUT (1)	Leaf (1)	Rts (1)	130:130	129:135	195:198	143:147	208:208	241:251	216:228	Eu2
MLG181	1	AUT325	AUT (1)	Leaf (1)	Rts (1)	127:130	129:132	195:198	143:147	205:205	251:251	210:222	Admixed
MLG182	1	AUT328	AUT (1)	Leaf (1)	Rts (1)	130:130	129:132	195:195	143:147	205:208	251:253	222:222	Admixed
MLG183	5	AUT329, AUT330, AUT331, AUT333, AUT334	AUT (5)	Leaf (5)	Rts (5)	124:130	129:132	195:204	143:145	202:205	251:253	210:210	Eu1
MLG184	1	AUT332	AUT (1)	Leaf (1)	Rts (1)	124:130	129:132	195:204	143:145	202:205	251:253	207:207	Admixed
MLG185	1	AUT335	AUT (1)	Leaf (1)	Rts (1)	124:130	129:132	195:204	143:145	205:205	251:253	210:210	Eu1
MLG186	1	AUT336	AUT (1)	Leaf (1)	Rts (1)	130:130	132:132	195:198	143:145	202:205	237:253	216:222	Admixed

MLG	N	Sample(s) Code[1]	Country[2]	Feeding form	Host plant[3]	PhyIII_55	PhyIII_30	PhyIII_36	DV8	Dvit6	DVSSR4	DV4	Genetic group (K=2)
MLG187	1	AUT337	AUT (1)	Leaf (1)	Rts (1)	115:133	135:135	195:195	145:145	208:208	251:251	222:222	Eu2
MLG188	1	AUT338	AUT (1)	Leaf (1)	Rts (1)	115:133	135:135	195:195	143:145	202:208	251:251	222:222	Eu2
MLG189	2	AUT339, AUT340	AUT (2)	Leaf (2)	Rts (2)	130:130	135:135	195:195	145:147	205:208	251:251	216:216	Eu2
MLG190	2	AUT341, AUT342	AUT (2)	Leaf (2)	Rts (2)	130:130	135:135	195:195	143:145	205:208	245:251	219:222	Eu2
MLG191	1	AUT343	AUT (1)	Leaf (1)	Rts (1)	130:130	135:135	195:195	145:145	202:202	241:251	222:222	Eu2
MLG192	3	AUT344, AUT345, AUT346	AUT (3)	Leaf (3)	Rts (3)	130:130	129:135	195:195	145:147	205:208	251:251	216:216	Eu2
MLG193	1	AUT347	AUT (1)	Leaf (1)	Rts (1)	127:127	129:132	195:198	145:147	202:205	251:253	216:219	Admixed
MLG194	1	AUT348	AUT (1)	Leaf (1)	Rts (1)	124:127	129:132	195:195	145:145	202:205	241:251	219:222	Admixed
MLG195	1	AUT349	AUT (1)	Leaf (1)	Rts (1)	121:130	129:132	195:195	145:145	205:208	241:251	219:228	Admixed
MLG196	1	AUT350	AUT (1)	Leaf (1)	Rts (1)	124:130	132:132	195:204	145:147	205:205	251:251	219:222	Admixed
MLG197	1	AUT351	AUT (1)	Leaf (1)	Rts (1)	130:130	129:129	195:195	145:147	205:205	251:251	216:216	Eu2
MLG198	1	AUT352	AUT (1)	Leaf (1)	Rts (1)	121:130	129:132	195:195	145:147	205:208	241:251	219:228	Admixed
MLG199	1	AUT353	AUT (1)	Leaf (1)	Rts (1)	124:130	129:129	195:198	145:145	202:202	251:251	216:222	Eu2
MLG200	2	AUT354, AUT361	AUT (2)	Leaf (2)	Rts (2)	121:121	129:132	195:195	143:145	202:205	251:251	219:222	Admixed
MLG201	1	AUT355	AUT (1)	Leaf (1)	Rts (1)	121:124	129:132	195:198	145:147	202:205	251:253	222:222	Admixed
MLG202	1	AUT356	AUT (1)	Leaf (1)	Rts (1)	121:121	132:135	195:195	147:147	202:202	251:251	216:219	Eu2
MLG203	1	AUT357	AUT (1)	Leaf (1)	Rts (1)	124:127	129:132	195:195	147:147	202:205	251:253	216:216	Admixed
MLG204	2	AUT358, AUT359	AUT (2)	Leaf (2)	Rts (2)	124:127	129:132	195:195	145:147	202:205	251:251	222:222	Admixed
MLG205	1	AUT360	AUT (1)	Leaf (1)	Rts (1)	124:127	129:132	195:195	143:145	202:205	251:251	216:222	Admixed
MLG206	1	AUT362	AUT (1)	Leaf (1)	Rts (1)	121:130	129:132	195:195	145:147	202:205	241:251	219:222	Admixed
MLG207	1	AUT363	AUT (1)	Leaf (1)	Rts (1)	121:130	132:132	195:204	145:145	202:205	253:253	216:222	Admixed
MLG208	2	AUT364, AUT368	AUT (2)	Leaf (2)	Rts (2)	115:124	129:135	198:204	143:145	202:202	251:251	216:222	Eu2
MLG209	1	AUT365	AUT (1)	Leaf (1)	Rts (1)	127:127	129:135	198:204	143:145	202:202	237:253	216:222	Eu2

MLG	N	Sample(s) Code[1]	Country[2]	Feeding form	Host plant[3]	PhyIII_55	PhyIII_30	PhyIII_36	DV8	Dvit6	DVSSR4	DV4	Genetic group (K=2)
MLG210	1	AUT366	AUT (1)	Leaf (1)	Rts (1)	115:124	129:135	198:204	143:143	202:202	251:251	216:222	Eu2
MLG211	1	AUT367	AUT (1)	Leaf (1)	Rts (1)	124:127	132:135	195:195	143:147	205:208	245:251	216:219	Admixed
MLG212	1	AUT369	AUT (1)	Leaf (1)	Rts (1)	127:127	129:132	195:195	143:143	202:205	251:253	210:222	Admixed
MLG213	1	AUT370	AUT (1)	Leaf (1)	Rts (1)	127:130	129:132	195:198	143:145	202:205	251:253	216:222	Admixed
MLG214	1	AUT371	AUT (1)	Leaf (1)	Rts (1)	115:127	129:132	195:198	143:145	202:202	251:253	216:219	Eu2
MLG215	1	AUT372	AUT (1)	Leaf (1)	Rts (1)	127:127	129:132	195:195	143:145	202:205	251:253	210:222	Admixed
MLG216	1	AUT373	AUT (1)	Leaf (1)	Rts (1)	127:130	126:129	195:198	143:145	202:205	251:253	216:222	Admixed
MLG217	2	AUT374, AUT375	AUT (2)	Leaf (2)	Rts (2)	115:127	126:126	195:198	143:145	202:202	251:253	216:219	Eu2
MLG218	1	AUT376	AUT (1)	Leaf (1)	Rts (1)	130:130	129:129	195:210	143:143	202:205	251:253	219:222	Admixed
MLG219	1	AUT377	AUT (1)	Leaf (1)	Rts (1)	130:130	129:129	195:210	143:143	202:205	251:253	222:222	Admixed
MLG220	1	AUT378	AUT (1)	Leaf (1)	Rts (1)	127:127	126:126	195:195	145:147	202:205	245:245	216:222	Admixed
MLG221	1	AUT379	AUT (1)	Leaf (1)	Rts (1)	130:130	132:132	195:210	143:143	202:205	253:253	207:222	Admixed
MLG222	1	AUT380	AUT (1)	Leaf (1)	Rts (1)	124:130	132:135	195:198	145:145	202:202	251:251	222:222	Eu2
MLG223	1	AUT381	AUT (1)	Leaf (1)	Rts (1)	130:130	129:132	195:198	143:145	202:205	245:251	216:222	Admixed
MLG224	1	AUT382	AUT (1)	Leaf (1)	Rts (1)	115:127	129:132	195:195	145:147	202:202	251:251	216:228	Eu2
MLG225	1	AUT383	AUT (1)	Leaf (1)	Rts (1)	130:130	129:129	195:210	143:143	202:205	251:251	222:222	Admixed
MLG226	3	AUT384, AUT387, AUT390	AUT (3)	Leaf (3)	Rts (3)	130:130	129:132	195:198	143:145	202:202	251:251	216:216	Eu2
MLG227	1	AUT385	AUT (1)	Leaf (1)	Rts (1)	124:127	129:132	195:195	143:145	205:205	249:249	216:219	Eu1
MLG228	1	AUT386	AUT (1)	Leaf (1)	Rts (1)	124:127	132:132	195:195	143:145	205:205	251:251	216:219	Eu1
MLG229	1	AUT388	AUT (1)	Leaf (1)	Rts (1)	127:130	129:132	195:195	143:145	208:208	245:251	216:228	Eu2
MLG230	1	AUT389	AUT (1)	Leaf (1)	Rts (1)	124:127	129:129	195:195	143:145	205:205	251:251	216:219	Admixed
MLG231	1	AUT391	AUT (1)	Leaf (1)	Rts (1)	124:127	129:129	183:195	143:145	205:205	251:251	216:219	Admixed
MLG232	1	DEU001	DEU (1)	Leaf (1)	Vvin (1)	130:130	132:132	198:207	143:145	202:208	253:253	210:219	Admixed
MLG233	1	DEU002	DEU (1)	Leaf (1)	Vvin (1)	121:130	132:132	195:195	145:147	205:208	241:253	210:216	Admixed
MLG234	1	DEU003	DEU (1)	Leaf (1)	Vvin (1)	121:130	132:132	195:195	143:145	202:205	251:253	210:225	Eu1

MLG	N	Sample(s) Code[1]	Country[2]	Feeding form	Host plant[3]	PhyIII_55	PhyIII_30	PhyIII_36	DV8	Dvit6	DVSSR4	DV4	Genetic group (K=2)
MLG235	1	DEU004	DEU (1)	Leaf (1)	Vvin (1)	130:130	132:132	195:195	143:143	205:208	251:253	210:222	Admixed
MLG236	1	DEU005	DEU (1)	Leaf (1)	Vvin (1)	121:130	129:129	195:195	143:145	202:208	245:253	210:219	Admixed
MLG237	3	DEU006, DEU007, DEU009	DEU (3)	Leaf (3)	Vvin (3)	124:127	129:132	195:195	143:145	208:208	241:245	216:219	Admixed
MLG238	1	DEU008	DEU (1)	Leaf (1)	Vvin (1)	130:130	132:132	198:204	145:147	202:205	245:251	210:216	Admixed
MLG239	1	DEU010	DEU (1)	Leaf (1)	Vvin (1)	130:130	129:132	195:195	143:147	208:208	245:251	216:219	Eu2
MLG240	1	DEU011	DEU (1)	Leaf (1)	Vvin (1)	124:130	132:132	195:195	143:143	208:208	245:245	216:216	Admixed
MLG241	2	DEU012, DEU013	DEU (2)	Leaf (2)	Vvin (2)	124:130	132:132	195:195	143:147	202:205	251:253	216:216	Admixed
MLG242	1	DEU014	DEU (1)	Leaf (1)	Vvin (1)	130:130	132:132	195:204	143:145	208:208	245:251	219:222	Admixed
MLG243	1	DEU015	DEU (1)	Leaf (1)	Vvin (1)	130:130	132:132	195:195	143:143	196:205	241:253	219:222	Eu1
MLG244	1	DEU016	DEU (1)	Leaf (1)	Vvin (1)	-:-	132:132	195:195	143:143	196:205	241:253	219:222	Eu1
MLG245	1	DEU017	DEU (1)	Leaf (1)	Vvin (1)	124:130	132:132	195:195	145:147	196:208	-:-	216:225	Admixed
MLG246	1	DEU018	DEU (1)	Leaf (1)	Vvin (1)	124:130	132:132	195:195	145:147	196:208	249:253	216:225	Eu1
MLG247	5	DEU019, DEU030, DEU031, DEU032, DEU056	DEU (5)	Leaf (5)	HDP (4), Vvin (1)	121:124	132:132	195:195	143:143	208:208	245:251	210:216	Admixed
MLG248	1	DEU020	DEU (1)	Leaf (1)	Vvin (1)	130:130	129:132	195:195	143:145	208:208	245:251	222:228	Eu2
MLG249	1	DEU021	DEU (1)	Leaf (1)	Vvin (1)	127:130	129:132	195:195	143:145	196:208	251:251	216:222	Admixed
MLG250	1	DEU022	DEU (1)	Leaf (1)	Vvin (1)	130:130	132:132	195:195	143:145	202:202	251:251	210:222	Admixed
MLG251	1	DEU023	DEU (1)	Leaf (1)	Vvin (1)	127:133	129:132	195:195	143:145	208:208	245:251	219:222	Eu2
MLG252	5	DEU024, DEU025, DEU026, DEU027, DEU028	DEU (5)	Leaf (5)	Vvin (5)	127:127	129:132	195:195	145:145	205:208	251:251	219:219	Admixed
MLG253	1	DEU029	DEU (1)	Leaf (1)	Vvin (1)	130:130	129:135	195:195	143:143	208:208	251:253	216:222	Eu2

MLG	N	Sample(s) Code[1]	Country[2]	Feeding form	Host plant[3]	PhyIII_55	PhyIII_30	PhyIII_36	DV8	Dvit6	DVSSR4	DV4	Genetic group (K=2)
MLG254	1	DEU033	DEU (1)	Leaf (1)	HDP (1)	124:130	132:132	195:207	143:143	208:208	251:251	219:222	Admixed
MLG255	1	DEU034	DEU (1)	Leaf (1)	HDP (1)	124:124	132:132	195:207	143:143	208:208	251:251	219:222	Admixed
MLG256	1	DEU035	DEU (1)	Leaf (1)	HDP (1)	130:130	132:132	195:195	143:145	208:208	251:253	213:222	Admixed
MLG257	1	DEU036	DEU (1)	Leaf (1)	HDP (1)	124:127	132:141	195:207	143:143	205:208	251:253	222:222	Eu1
MLG258	1	DEU037	DEU (1)	Leaf (1)	HDP (1)	124:130	120:132	195:204	143:145	205:205	251:251	216:228	Admixed
MLG259	2	DEU038, DEU039	DEU (2)	Leaf (2)	HDP (2)	130:130	132:132	195:195	143:145	205:205	241:251	216:222	Admixed
MLG260	1	DEU040	DEU (1)	Leaf (1)	HDP (1)	124:130	132:141	195:207	143:143	202:202	251:251	216:222	Admixed
MLG261	1	DEU041	DEU (1)	Leaf (1)	HDP (1)	124:127	132:141	195:207	143:143	202:202	251:251	216:222	Admixed
MLG262	4	DEU042, DEU043, DEU044, DEU045	DEU (4)	Leaf (4)	HDP (4)	124:127	132:132	195:195	143:145	208:208	251:251	222:222	Admixed
MLG263	1	DEU046	DEU (1)	Leaf (1)	HDP (1)	124:127	132:132	195:204	143:143	202:202	245:251	216:222	Admixed
MLG264	3	DEU047, DEU050, DEU052	DEU (3)	Leaf (3)	HDP (3)	124:127	132:135	195:207	143:147	202:202	251:253	222:222	Admixed
MLG265	1	DEU048	DEU (1)	Leaf (1)	HDP (1)	124:127	132:135	195:207	143:143	202:208	251:251	219:222	Admixed
MLG266	1	DEU049	DEU (1)	Leaf (1)	HDP (1)	124:127	132:135	195:207	143:145	202:202	251:253	216:222	Admixed
MLG267	1	DEU051	DEU (1)	Leaf (1)	HDP (1)	124:127	129:132	195:195	143:145	202:208	251:253	210:219	Admixed
MLG268	3	DEU053, DEU054, DEU055	DEU (3)	Leaf (3)	HDP (3)	127:130	132:132	195:204	143:145	202:202	245:251	216:222	Admixed
MLG269	1	DEU057	DEU (1)	Leaf (1)	HDP (1)	124:127	120:120	195:195	145:147	205:205	241:251	216:219	Eu1
MLG270	1	DEU058	DEU (1)	Leaf (1)	HDP (1)	124:127	132:132	195:198	145:147	205:205	241:251	216:222	Admixed
MLG271	7	DEU059, DEU061, DEU064, DEU066, DEU067,	DEU (7)	Leaf (7)	HDP (7)	124:127	132:132	195:207	143:145	202:205	251:253	222:222	Eu1

MLG	N	Sample(s) Code[1]	Country[2]	Feeding form	Host plant[3]	PhyIII_55	PhyIII_30	PhyIII_36	DV8	Dvit6	DVSSR4	DV4	Genetic group (K=2)
		DEU068, DEU069											
MLG272	3	DEU060, DEU062, DEU063	DEU (3)	Leaf (3)	HDP (3)	127:130	132:132	195:204	143:145	205:205	251:253	216:222	Eu1
MLG273	4	DEU065, DEU088, DEU090, DEU092	DEU (4)	Leaf (4)	HDP (4)	124:127	132:132	198:198	145:145	202:202	245:251	216:222	Admixed
MLG274	8	DEU069, DEU070, DEU071, DEU072, DEU073, DEU074, DEU075, DEU076	DEU (8)	Leaf (8)	HDP (8)	124:124	132:132	195:207	145:147	205:205	241:251	216:216	Eu1
MLG275	1	DEU077	DEU (1)	Leaf (1)	HDP (1)	124:130	132:132	195:195	143:145	205:205	245:245	213:222	Eu1
MLG276	2	DEU078, DEU081	DEU (2)	Leaf (2)	HDP (2)	124:127	132:132	195:201	145:145	205:205	245:251	210:219	Eu1
MLG277	1	DEU079	DEU (1)	Leaf (1)	HDP (1)	127:127	132:132	195:207	145:145	205:205	251:253	222:222	Eu1
MLG278	1	DEU080	DEU (1)	Leaf (1)	HDP (1)	127:127	132:132	207:207	145:145	202:208	241:251	216:222	Eu1
MLG279	1	DEU082	DEU (1)	Leaf (1)	HDP (1)	124:130	132:132	204:204	145:147	202:205	251:251	210:222	Admixed
MLG280	1	DEU083	DEU (1)	Leaf (1)	HDP (1)	124:124	120:132	195:204	145:147	205:205	251:251	216:216	Admixed
MLG281	1	DEU084	DEU (1)	Leaf (1)	HDP (1)	124:130	132:132	195:207	143:145	205:208	245:245	213:222	Eu1
MLG282	1	DEU085	DEU (1)	Leaf (1)	HDP (1)	124:127	132:132	195:195	143:145	205:208	251:253	219:222	Admixed
MLG283	1	DEU086	DEU (1)	Leaf (1)	HDP (1)	124:130	132:132	195:207	143:145	202:205	251:251	216:222	Eu1
MLG284	1	DEU087	DEU (1)	Leaf (1)	HDP (1)	124:124	132:132	207:207	145:145	205:205	245:245	219:222	Eu1
MLG285	1	DEU091	DEU (1)	Leaf (1)	HDP (1)	124:124	132:132	195:207	143:145	202:205	251:251	219:222	Eu1
MLG286	1	DEU093	DEU (1)	Leaf (1)	HDP (1)	124:127	132:132	195:198	145:145	202:208	251:253	216:222	Admixed
MLG287	1	DEU094	DEU (1)	Leaf (1)	Vvin (1)	124:124	129:132	195:195	143:145	205:205	251:251	219:222	Admixed

MLG	N	Sample(s) Code[1]	Country[2]	Feeding form	Host plant[3]	PhyIII_55	PhyIII_30	PhyIII_36	DV8	Dvit6	DVSSR4	DV4	Genetic group (K=2)
MLG288	1	DEU095	DEU (1)	Leaf (1)	Vvin (1)	130:130	129:132	195:198	145:145	196:205	241:251	222:222	Admixed
MLG289	1	DEU096	DEU (1)	Leaf (1)	Vvin (1)	127:130	132:132	195:201	143:145	196:202	251:253	216:222	Eu1
MLG290	1	DEU097	DEU (1)	Root (1)	Vvin (1)	124:130	129:129	195:195	145:145	196:208	241:251	222:222	Admixed
MLG291	1	DEU098	DEU (1)	Root (1)	Vvin (1)	124:130	129:129	195:195	145:145	208:208	241:251	222:222	Eu2
MLG292	1	DEU099	DEU (1)	Leaf (1)	Vvin (1)	127:130	132:132	195:195	147:147	208:208	251:253	216:228	Eu2
MLG293	1	DEU100	DEU (1)	Leaf (1)	Vvin (1)	130:130	129:132	195:207	143:145	202:208	251:253	216:228	Admixed
MLG294	1	DEU101	DEU (1)	Leaf (1)	Vvin (1)	124:127	132:132	195:207	143:145	202:208	251:251	222:228	Admixed
MLG295	1	DEU102	DEU (1)	Root (1)	Vvin (1)	127:130	132:132	204:207	143:145	202:208	245:251	216:222	Admixed
MLG296	1	DEU103	DEU (1)	Root (1)	Vvin (1)	127:130	129:132	195:195	145:147	205:208	251:251	219:228	Eu2
MLG297	1	DEU104	DEU (1)	Leaf (1)	HDP (1)	121:124	132:132	195:207	145:145	205:205	251:251	222:222	Eu1
MLG298	1	DEU105	DEU (1)	Leaf (1)	HDP (1)	124:130	132:132	195:195	143:143	205:208	241:251	210:216	Eu1
MLG299	1	DEU106	DEU (1)	Leaf (1)	HDP (1)	127:127	132:132	195:207	143:149	202:205	245:251	216:228	Eu1
MLG300	1	DEU107	DEU (1)	Leaf (1)	HDP (1)	130:130	132:132	195:204	143:145	205:208	251:251	216:222	Admixed
MLG301	1	DEU108	DEU (1)	Root (1)	Vvin (1)	124:130	132:132	195:210	143:145	208:208	251:251	219:222	Admixed
MLG302	1	DEU109	DEU (1)	Leaf (1)	HDP (1)	124:124	129:132	195:195	143:145	202:205	245:251	210:213	Admixed
MLG303	1	DEU110	DEU (1)	Leaf (1)	HDP (1)	127:127	129:132	195:195	147:147	202:202	245:251	213:216	Admixed
MLG304	1	DEU111	DEU (1)	Leaf (1)	HDP (1)	121:121	120:129	195:198	143:147	196:202	251:253	210:222	Admixed
MLG305	1	DEU112	DEU (1)	Leaf (1)	HDP (1)	127:127	120:132	195:198	143:147	202:205	251:251	210:222	Admixed
MLG306	1	DEU113	DEU (1)	Root (1)	HDP (1)	121:127	132:132	195:195	143:145	202:208	245:251	216:222	Admixed
MLG307	1	DEU114	DEU (1)	Leaf (1)	Vvin (1)	124:130	123:129	195:198	143:145	202:208	251:253	216:222	Eu2
MLG308	2	DEU115, DEU116	DEU (2)	Leaf (2)	Vvin (2)	124:130	132:132	195:198	143:145	202:208	251:253	216:222	Admixed
MLG309	1	DEU117	DEU (1)	Leaf (1)	Vvin (1)	124:130	132:132	195:198	145:145	202:208	251:253	216:222	Admixed
MLG310	2	CHE001, CHE002	CHE (2)	Leaf (2)	HDP (2)	127:130	132:132	195:201	143:143	205:205	251:253	222:222	Eu1
MLG311	1	CHE003	CHE (1)	Leaf (1)	HDP (1)	130:130	132:132	195:195	143:147	205:208	-:-	222:222	Admixed
MLG312	1	CHE004	CHE (1)	Leaf (1)	Vvin (1)	123:138	132:141	195:195	143:147	202:205	251:253	216:222	Admixed

MLG	N	Sample(s) Code[1]	Country[2]	Feeding form	Host plant[3]	PhyIII_55	PhyIII_30	PhyIII_36	DV8	Dvit6	DVSSR4	DV4	Genetic group (K=2)
MLG313	1	CHE005	CHE (1)	Leaf (1)	Vvin (1)	115:124	129:132	195:195	-:-	202:202	-:-	222:222	Admixed
MLG314	2	CHE006, CHE009	CHE (2)	Leaf (2)	Rts (2)	124:124	132:132	195:195	145:145	202:208	245:251	219:222	Admixed
MLG315	1	CHE007	CHE (1)	Leaf (1)	Rts (1)	124:124	129:132	195:195	145:145	202:202	251:251	219:219	Admixed
MLG316	1	CHE008	CHE (1)	Leaf (1)	Rts (1)	124:124	132:132	195:210	143:145	196:202	241:251	219:219	Eu1
MLG317	4	CHE010, CHE012, CHE013, CHE014	CHE (4)	Leaf (4)	HDP (4)	124:130	129:132	195:195	143:143	202:208	251:251	216:219	Admixed
MLG318	2	CHE011, CHE015	CHE (2)	Leaf (1), Root (1)	HDP (2)	121:127	132:132	195:195	143:143	205:205	251:251	216:222	Admixed
MLG319	1	CHE016	CHE (1)	Root (1)	HDP (1)	127:127	132:132	195:195	143:145	202:202	251:251	210:216	Admixed
MLG320	1	CHE017	CHE (1)	Root (1)	HDP (1)	124:127	129:129	195:195	143:145	205:205	245:253	219:222	Admixed
MLG321	1	CHE018	CHE (1)	Root (1)	HDP (1)	124:130	129:132	195:195	147:147	202:205	251:251	219:222	Admixed
MLG322	4	CHE019, CHE021, CHE022, CHE023	CHE (4)	Leaf (4)	HDP (4)	121:124	129:132	195:207	143:145	202:208	251:251	222:222	Admixed
MLG323	1	CHE020	CHE (1)	Leaf (1)	HDP (1)	124:127	132:132	195:207	145:145	208:208	251:253	210:222	Eu1
MLG324	2	CHE024, CHE029	CHE (2)	Root (2)	HDP (2)	124:127	132:132	195:195	143:147	205:205	241:251	210:222	Eu1
MLG325	2	CHE025, CHE044	CHE (2)	Root (2)	HDP (2)	124:130	132:132	195:195	143:145	202:205	251:251	216:219	Admixed
MLG326	1	CHE026	CHE (1)	Root (1)	HDP (1)	124:130	129:132	195:195	143:145	205:208	251:251	219:219	Admixed
MLG327	1	CHE027	CHE (1)	Root (1)	HDP (1)	121:121	132:132	195:195	143:145	196:208	245:253	210:222	Eu1
MLG328	1	CHE028	CHE (1)	Root (1)	HDP (1)	121:121	132:132	195:207	145:145	202:205	245:251	210:210	Eu1
MLG329	9	CHE030, CHE031, CHE032, CHE033, CHE034, CHE047,	CHE (9)	Leaf (5), Root (4)	HDP (9)	121:124	129:132	195:195	143:143	202:205	251:251	210:222	Admixed

MLG	N	Sample(s) Code[1]	Country[2]	Feeding form	Host plant[3]	PhyIII_55	PhyIII_30	PhyIII_36	DV8	Dvit6	DVSSR4	DV4	Genetic group (K=2)
		CHE048, CHE049, CHE051											
MLG330	1	CHE035	CHE (1)	Root (1)	HDP (1)	121:124	129:132	195:195	143:143	202:205	251:251	222:222	Admixed
MLG331	1	CHE036	CHE (1)	Root (1)	HDP (1)	124:127	132:132	195:195	143:143	202:208	251:251	219:222	Admixed
MLG332	1	CHE037	CHE (1)	Root (1)	HDP (1)	124:127	132:132	195:195	143:143	202:208	251:251	219:219	Admixed
MLG333	1	CHE038	CHE (1)	Root (1)	HDP (1)	127:130	129:132	195:207	143:143	205:205	245:245	222:222	Eu1
MLG334	2	CHE039, CHE040	CHE (2)	Root (2)	HDP (2)	121:124	132:132	195:195	143:145	202:205	251:253	210:210	Eu1
MLG335	1	CHE041	CHE (1)	Root (1)	HDP (1)	124:127	132:132	195:207	143:143	208:208	245:253	210:222	Eu1
MLG336	1	CHE042	CHE (1)	Root (1)	HDP (1)	124:127	132:132	183:195	145:147	205:205	251:253	216:216	Admixed
MLG337	1	CHE043	CHE (1)	Root (1)	HDP (1)	121:124	132:132	195:195	143:143	196:196	251:253	210:222	Eu1
MLG338	1	CHE045	CHE (1)	Root (1)	HDP (1)	121:124	129:132	183:195	143:143	202:205	251:251	210:222	Admixed
MLG339	1	CHE046	CHE (1)	Root (1)	HDP (1)	127:127	129:132	195:195	143:143	205:208	251:253	210:216	Admixed
MLG340	1	CHE050	CHE (1)	Root (1)	HDP (1)	127:127	132:132	195:204	143:147	196:205	245:253	216:222	Eu1
MLG341	1	CHE052	CHE (1)	Root (1)	HDP (1)	124:130	132:132	195:195	143:145	196:205	245:245	210:222	Eu1
MLG342	5	CHE053, CHE054, CHE055, CHE056, CHE057	CHE (5)	Leaf (5)	RGV (5)	124:124	132:135	195:195	143:145	202:205	241:245	210:222	Admixed
MLG343	1	CHE058	CHE (1)	Root (1)	RGV (1)	127:130	132:132	195:195	145:145	202:205	245:251	216:222	Admixed
MLG344	1	CHE059	CHE (1)	Root (1)	RGV (1)	130:130	132:132	195:195	143:147	202:202	251:251	222:222	Admixed
MLG345	3	CHE060, CHE061, CHE062	CHE (3)	Root (3)	RGV (3)	127:127	132:135	195:195	143:145	202:208	241:251	210:222	Admixed
MLG346	1	CHE063	CHE (1)	Leaf (1)	RGV (1)	124:130	129:132	195:195	145:147	205:208	245:251	222:228	Admixed
MLG347	2	CHE064, CHE065	CHE (2)	Leaf (2)	RGV (2)	127:130	129:135	195:195	145:145	205:208	241:251	219:222	Admixed
MLG348	1	CHE066	CHE (1)	Leaf (1)	RGV (1)	124:127	132:132	195:204	145:145	208:208	251:251	222:222	Admixed

MLG	N	Sample(s) Code[1]	Country[2]	Feeding form	Host plant[3]	PhyIII_55	PhyIII_30	PhyIII_36	DV8	Dvit6	DVSSR4	DV4	Genetic group (K=2)
MLG349	1	CHE067	CHE (1)	Leaf (1)	RGV (1)	124:127	132:132	195:204	145:145	208:208	251:251	219:222	Admixed
MLG350	2	CHE068, CHE069	CHE (2)	Root (2)	Vvin (2)	124:127	132:132	195:195	147:147	202:208	251:253	219:222	Admixed
MLG351	4	CHE070, CHE071, CHE073, CHE074	CHE (4)	Leaf (4)	Vvin (4)	127:130	132:132	195:195	145:147	205:208	251:251	216:222	Admixed
MLG352	1	CHE072	CHE (1)	Leaf (1)	Vvin (1)	124:127	132:132	195:195	143:143	202:208	241:251	222:222	Admixed
MLG353	1	CHE075	CHE (1)	Leaf (1)	HDP (1)	115:127	132:132	195:195	143:143	205:208	241:253	222:222	Admixed
MLG354	1	CHE076	CHE (1)	Leaf (1)	HDP (1)	127:130	132:132	195:201	145:147	205:205	241:251	222:222	Eu1
MLG355	1	CHE077	CHE (1)	Leaf (1)	HDP (1)	124:130	132:132	201:204	143:145	202:205	251:251	219:222	Eu1
MLG356	1	CHE078	CHE (1)	Leaf (1)	HDP (1)	130:130	129:132	195:195	145:147	205:208	251:253	216:222	Admixed
MLG357	1	CHE079	CHE (1)	Leaf (1)	HDP (1)	130:130	129:132	195:195	145:147	205:208	253:253	216:222	Admixed
MLG358	1	CHE080	CHE (1)	Root (1)	HDP (1)	127:130	132:132	195:201	143:147	205:208	241:251	219:222	Eu1
MLG359	1	CHE081	CHE (1)	Leaf (1)	Vvin (1)	127:130	129:132	195:204	143:145	202:208	251:251	219:222	Admixed
MLG360	5	CHE082, CHE083, CHE084, CHE085, CHE087	CHE (5)	Leaf (4), Root (1)	Rts (1), Vvin (4)	127:130	132:132	195:204	143:145	208:208	241:251	219:222	Admixed
MLG361	1	CHE086	CHE (1)	Root (1)	Rts (1)	127:127	132:132	195:195	143:145	205:205	241:251	219:222	Eu1
MLG362	1	CHE088	CHE (1)	Leaf (1)	HDP (1)	124:127	132:132	207:213	143:145	205:208	251:251	213:219	Eu1
MLG363	1	CHE089	CHE (1)	Leaf (1)	HDP (1)	121:127	129:132	195:195	143:145	202:202	251:251	213:222	Admixed
MLG364	1	CHE090	CHE (1)	Leaf (1)	HDP (1)	127:127	129:132	195:204	143:145	196:202	245:253	222:222	Eu1
MLG365	1	CHE091	CHE (1)	Leaf (1)	HDP (1)	121:124	132:132	195:195	143:145	208:208	251:253	219:222	Admixed
MLG366	1	CHE092	CHE (1)	Leaf (1)	HDP (1)	124:127	132:132	195:213	143:145	202:208	251:251	219:222	Admixed
MLG367	2	CHE093, CHE094	CHE (2)	Leaf (2)	HDP (2)	124:127	132:132	195:213	143:145	202:208	251:251	222:222	Admixed
MLG368	1	CHE095	CHE (1)	Leaf (1)	HDP (1)	121:124	132:132	195:195	147:147	202:202	255:255	222:222	Admixed
MLG369	1	CHE096	CHE (1)	Leaf (1)	HDP (1)	121:124	132:132	195:195	145:145	202:202	251:251	222:222	Admixed

MLG	N	Sample(s) Code[1]	Country[2]	Feeding form	Host plant[3]	PhyIII_55	PhyIII_30	PhyIII_36	DV8	Dvit6	DVSSR4	DV4	Genetic group (K=2)
MLG370	1	CHE097	CHE (1)	Root (1)	n.a. (1)	124:127	132:132	195:195	143:145	202:202	251:251	222:222	Admixed
MLG371	1	CHE098	CHE (1)	Leaf (1)	HDP (1)	124:127	132:132	201:213	145:145	202:205	251:251	210:210	Eu1
MLG372	4	CHE099, CHE100, CHE101, CHE102	CHE (4)	Leaf (4)	HDP (4)	124:127	132:132	195:201	143:147	205:205	251:251	210:210	Eu1
MLG373	1	CHE103	CHE (1)	Root (1)	HDP (1)	124:127	132:132	201:201	145:145	202:205	251:251	210:222	Eu1
MLG374	7	CHE104, CHE105, CHE106, CHE107, CHE109, CHE110, CHE111	CHE (7)	Root (7)	HDP (7)	124:127	132:132	195:201	145:145	205:205	251:253	222:222	Eu1
MLG375	1	CHE108	CHE (1)	Root (1)	HDP (1)	124:127	132:132	201:213	145:145	202:205	251:251	210:222	Eu1
MLG376	5	CHE112, CHE113 CHE114, CHE115, CHE116	CHE (5)	Leaf (5)	Rts (5)	124:130	132:132	201:201	143:145	205:205	241:251	222:222	Eu1
MLG377	5	CHE117, CHE118, CHE119, CHE120, CHE121	CHE (5)	Leaf (5)	HDP (5)	127:133	132:132	195:207	145:145	202:205	251:253	222:222	Admixed
MLG378	1	CHE122	CHE (1)	Root (1)	Rts (1)	127:133	129:132	195:207	145:145	202:205	251:253	222:222	Admixed
MLG379	4	CHE123, CHE124, CHE125, CHE126	CHE (4)	Root (4)	Rts (4)	127:133	129:132	195:207	145:147	202:205	251:253	222:222	Admixed
MLG380	16	CHE127, CHE128, CHE129, HRV024,	CHE (4), HRV (12)	Leaf (16)	HDP (4), Rts (12)	124:130	132:132	195:204	145:145	202:202	251:251	222:222	Admixed

MLG	N	Sample(s) Code[1]	Country[2]	Feeding form	Host plant[3]	PhyIII_55	PhyIII_30	PhyIII_36	DV8	Dvit6	DVSSR4	DV4	Genetic group (K=2)
		HRV025, HRV027, HRV028, HRV029, HRV030, HRV031, HRV032, HRV033, HRV035, HRV036, HRV044											
MLG381	6	CHE131, CHE132, CHE133, CHE134, CHE135, CHE141	CHE (6)	Leaf (6)	HDP (5), Vvin (1)	121:130	129:132	195:204	145:145	202:208	251:251	222:222	Admixed
MLG382	2	CHE136, CHE137	CHE (2)	Leaf (2)	Vvin (2)	115:124	129:132	195:207	143:143	202:202	251:251	222:222	Admixed
MLG383	1	CHE138	CHE (1)	Leaf (1)	Vvin (1)	124:130	129:132	195:204	143:145	202:202	251:251	222:222	Admixed
MLG384	1	CHE139	CHE (1)	Leaf (1)	Vvin (1)	130:130	129:132	204:204	143:145	202:208	251:251	222:222	Admixed
MLG385	2	CHE140, CHE142	CHE (2)	Leaf (2)	Vvin (2)	124:130	132:132	195:195	143:145	202:208	251:251	213:222	Admixed
MLG386	1	CHE143	CHE (1)	Leaf (1)	Vvin (1)	124:130	132:132	195:195	143:145	202:208	251:253	213:222	Admixed
MLG387	2	CHE144, CHE145	CHE (2)	Leaf (2)	n.a. (2)	124:124	129:132	195:195	145:147	202:202	251:251	210:210	Admixed
MLG388	1	CHE146	CHE (1)	Leaf (1)	n.a. (1)	124:130	132:132	195:204	143:145	202:202	251:251	210:222	Admixed
MLG389	1	CHE147	CHE (1)	Leaf (1)	n.a. (1)	130:130	129:132	195:195	143:145	202:205	241:253	210:210	Admixed
MLG390	1	CHE148	CHE (1)	Leaf (1)	HDP (1)	124:130	129:132	183:195	143:147	202:205	251:251	219:219	Admixed
MLG391	1	CHE149	CHE (1)	Leaf (1)	HDP (1)	115:124	132:132	195:195	143:143	202:202	251:251	222:222	Admixed
MLG392	1	CHE150	CHE (1)	Leaf (1)	HDP (1)	124:130	129:132	183:195	143:147	202:205	251:251	219:222	Admixed
MLG393	1	CHE151	CHE (1)	Leaf (1)	HDP (1)	127:127	132:135	195:195	143:145	202:208	251:253	216:222	Admixed

MLG	N	Sample(s) Code[1]	Country[2]	Feeding form	Host plant[3]	PhyIII_55	PhyIII_30	PhyIII_36	DV8	Dvit6	DVSSR4	DV4	Genetic group (K=2)
MLG394	7	CHE152, CHE154, CHE155, CHE156, CHE157, CHE158, CHE159	CHE (7)	Leaf (7)	HDP (5), Rts (2)	124:130	132:132	183:195	143:147	205:205	251:253	219:222	Admixed
MLG395	1	CHE153	CHE (1)	Leaf (1)	Rts (1)	124:130	132:132	195:204	143:147	202:202	245:251	222:222	Admixed
MLG396	4	CHE160, CHE161, CHE162, CHE163	CHE (4)	Leaf (4)	HDP (4)	124:127	132:135	195:195	143:145	202:208	241:253	210:222	Admixed
MLG397	1	CHE164	CHE (1)	Leaf (1)	HDP (1)	124:127	132:135	201:201	143:145	202:208	241:253	210:222	Eu1
MLG398	1	CHE165	CHE (1)	Leaf (1)	HDP (1)	124:130	132:132	195:195	143:145	205:208	249:249	216:219	Eu1
MLG399	1	CHE166	CHE (1)	Leaf (1)	HDP (1)	124:130	132:132	195:195	143:145	205:208	251:251	219:222	Admixed
MLG400	1	CHE167	CHE (1)	Leaf (1)	Rts (1)	124:130	132:132	195:207	145:147	202:202	251:251	210:219	Admixed
MLG401	1	CHE168	CHE (1)	Leaf (1)	Rts (1)	124:124	132:132	183:195	143:147	202:205	253:253	219:222	Admixed
MLG402	1	CHE169	CHE (1)	Leaf (1)	Rts (1)	124:130	132:132	195:207	143:143	202:202	251:253	219:219	Admixed
MLG403	1	CHE170	CHE (1)	Leaf (1)	Rts (1)	124:127	132:132	195:207	143:143	202:202	251:251	210:219	Eu1
MLG404	1	CHE171	CHE (1)	Leaf (1)	Rts (1)	124:124	132:132	195:204	145:145	202:202	251:251	222:222	Admixed
MLG405	1	CHE172	CHE (1)	Leaf (1)	Rts (1)	124:127	132:132	195:213	143:147	205:205	251:251	210:210	Eu1
MLG406	1	CHE173	CHE (1)	Leaf (1)	Rts (1)	127:130	132:132	195:195	147:147	202:205	251:251	210:222	Admixed
MLG407	4	CHE174, CHE176, CHE177, CHE178	CHE (4)	Leaf (4)	HDP (4)	130:130	129:132	195:201	143:145	202:202	245:251	219:222	Admixed
MLG408	1	CHE175	CHE (1)	Leaf (1)	HDP (1)	130:130	129:132	195:201	143:145	202:202	251:253	219:222	Admixed
MLG409	4	CHE179, CHE180, CHE183, CHE184	CHE (4)	Root (4)	HDP (4)	127:130	120:132	195:195	143:143	202:205	251:251	222:222	Admixed

MLG	N	Sample(s) Code[1]	Country[2]	Feeding form	Host plant[3]	PhyIII_55	PhyIII_30	PhyIII_36	DV8	Dvit6	DVSSR4	DV4	Genetic group (K=2)
MLG410	2	CHE181, CHE182	CHE (2)	Root (2)	HDP (2)	127:130	120:132	195:195	143:143	205:208	257:257	225:225	Eu1
MLG411	1	CHE185	CHE (1)	Root (1)	HDP (1)	127:130	120:132	195:195	143:143	202:205	249:249	219:219	Eu1
MLG412	1	CHE186	CHE (1)	Leaf (1)	HDP (1)	127:127	129:132	195:207	145:145	205:205	253:253	219:222	Eu1
MLG413	1	CHE187	CHE (1)	Leaf (1)	HDP (1)	127:127	129:132	195:207	145:145	205:205	241:253	219:222	Eu1
MLG414	1	CHE188	CHE (1)	Leaf (1)	HDP (1)	127:127	132:132	195:207	143:145	202:205	241:253	219:222	Eu1
MLG415	1	CHE189	CHE (1)	Leaf (1)	HDP (1)	127:130	132:132	195:207	145:145	202:205	241:253	219:222	Eu1
MLG416	1	CHE190	CHE (1)	Leaf (1)	HDP (1)	127:130	129:132	195:207	145:145	202:205	241:253	219:222	Eu1
MLG417	3	CHE191, CHE197, CHE198	CHE (3)	Root (3)	Rts (3)	127:130	132:132	195:207	145:145	202:205	253:253	219:222	Eu1
MLG418	1	CHE192	CHE (1)	Root (1)	Rts (1)	130:130	129:132	195:207	143:145	202:202	251:253	219:222	Admixed
MLG419	1	CHE193	CHE (1)	Root (1)	Rts (1)	127:130	132:132	207:207	143:145	202:205	241:253	219:222	Eu1
MLG420	2	CHE194, CHE195	CHE (2)	Root (2)	Rts (2)	124:127	132:132	195:207	145:145	202:205	241:245	219:219	Eu1
MLG421	1	CHE196	CHE (1)	Root (1)	Rts (1)	127:130	132:132	195:195	145:145	202:205	241:251	219:222	Admixed
MLG422	1	CHE199	CHE (1)	Root (1)	Rts (1)	127:130	132:132	195:207	145:145	202:205	245:253	219:222	Eu1
MLG423	1	CHE200	CHE (1)	Root (1)	Rts (1)	127:130	132:132	195:207	145:145	202:205	243:251	216:219	Eu1
MLG424	1	CHE201	CHE (1)	Leaf (1)	HDP (1)	124:130	132:132	195:195	145:145	202:202	251:253	222:222	Admixed
MLG425	1	CHE202	CHE (1)	Leaf (1)	HDP (1)	124:127	132:132	195:204	145:145	202:208	251:253	222:222	Admixed
MLG426	1	CHE203	CHE (1)	Leaf (1)	HDP (1)	124:127	132:132	195:207	145:145	202:202	241:251	219:222	Eu1
MLG427	1	CHE204	CHE (1)	Leaf (1)	HDP (1)	130:130	132:132	195:207	145:145	202:205	251:253	222:222	Eu1
MLG428	1	CHE205	CHE (1)	Leaf (1)	HDP (1)	130:130	132:132	195:195	143:145	202:202	251:251	219:219	Admixed
MLG429	1	CHE207	CHE (1)	Root (1)	HDP (1)	130:130	132:132	195:204	143:145	205:208	241:253	222:222	Admixed
MLG430	4	CHE208, CHE209, CHE210, CHE211	CHE (4)	Root (4)	HDP (4)	130:130	132:132	195:207	143:145	202:205	241:251	219:222	Eu1
MLG431	1	CHE212	CHE (1)	Root (1)	HDP (1)	127:130	132:132	195:195	143:145	202:202	251:253	219:222	Admixed

MLG	N	Sample(s) Code[1]	Country[2]	Feeding form	Host plant[3]	PhyIII_55	PhyIII_30	PhyIII_36	DV8	Dvit6	DVSSR4	DV4	Genetic group (K=2)
MLG432	1	CHE213	CHE (1)	Root (1)	HDP (1)	127:130	129:132	195:195	143:145	202:202	251:251	219:219	Admixed
MLG433	1	CHE214	CHE (1)	Root (1)	HDP (1)	130:130	132:132	195:207	145:145	202:202	241:251	219:219	Admixed
MLG434	1	CHE215	CHE (1)	Root (1)	HDP (1)	121:124	132:132	195:204	145:145	202:208	251:253	222:222	Admixed
MLG435	1	CHE216	CHE (1)	Leaf (1)	HDP (1)	124:130	132:132	195:207	145:145	208:208	241:253	219:222	Admixed
MLG436	1	CHE217	CHE (1)	Leaf (1)	HDP (1)	127:130	132:132	207:213	145:145	202:208	241:241	222:222	Eu1
MLG437	1	CHE218	CHE (1)	Leaf (1)	HDP (1)	124:127	132:132	207:207	143:145	208:208	251:253	222:222	Eu1
MLG438	1	CHE219	CHE (1)	Leaf (1)	HDP (1)	124:124	132:132	195:195	145:145	196:196	251:251	222:222	Eu1
MLG439	1	CHE220	CHE (1)	Leaf (1)	HDP (1)	130:130	132:132	213:213	145:145	202:202	241:241	222:222	Admixed
MLG440	1	CHE221	CHE (1)	Leaf (1)	HDP (1)	124:130	132:132	195:195	143:145	196:202	251:251	222:222	Eu1
MLG441	3	CHE222, CHE228, CHE229	CHE (3)	Root (3)	Rts (3)	124:127	132:132	195:207	145:145	202:208	241:253	222:222	Eu1
MLG442	1	CHE223	CHE (1)	Root (1)	Rts (1)	127:130	132:132	195:213	143:145	196:202	241:251	222:222	Eu1
MLG443	1	CHE224	CHE (1)	Root (1)	Rts (1)	124:124	132:132	195:207	143:145	196:208	251:253	222:222	Eu1
MLG444	1	CHE225	CHE (1)	Root (1)	Rts (1)	124:130	132:132	201:207	143:145	205:208	251:253	222:222	Eu1
MLG445	2	CHE226, CHE227	CHE (2)	Root (2)	Rts (2)	130:130	132:132	195:207	143:145	196:208	241:251	219:222	Eu1
MLG446	1	CHE230	CHE (1)	Root (1)	Rts (1)	124:130	132:132	195:195	143:145	196:202	241:241	222:222	Eu1
MLG447	1	CHE231	CHE (1)	Root (1)	Rts (1)	124:124	132:132	195:213	145:147	208:208	251:253	216:222	Admixed
MLG448	1	CHE232	CHE (1)	Leaf (1)	HDP (1)	127:127	132:132	195:207	143:147	202:205	251:253	210:219	Eu1
MLG449	1	CHE233	CHE (1)	Leaf (1)	HDP (1)	124:127	132:132	195:207	143:145	202:202	251:251	210:219	Eu1
MLG450	2	CHE234, CHE235	CHE (2)	Leaf (2)	HDP (2)	124:124	132:132	183:204	143:145	205:205	251:251	219:222	Admixed
MLG451	1	CHE236	CHE (1)	Leaf (1)	HDP (1)	127:130	132:132	195:204	143:145	205:205	251:253	210:219	Eu1
MLG452	1	CHE237	CHE (1)	Root (1)	Rts (1)	124:124	132:132	195:195	143:145	202:205	251:251	210:219	Eu1
MLG453	1	CHE238	CHE (1)	Root (1)	Rts (1)	124:124	132:132	204:204	143:145	205:205	253:253	219:219	Eu1
MLG454	1	CHE239	CHE (1)	Root (1)	Rts (1)	124:127	129:132	195:195	143:147	202:205	251:253	210:222	Admixed
MLG455	1	CHE240	CHE (1)	Root (1)	Rts (1)	124:130	129:132	195:204	143:143	202:205	251:251	210:222	Admixed

MLG	N	Sample(s) Code[1]	Country[2]	Feeding form	Host plant[3]	PhyIII_55	PhyIII_30	PhyIII_36	DV8	Dvit6	DVSSR4	DV4	Genetic group (K=2)
MLG456	1	CHE241	CHE (1)	Root (1)	Rts (1)	124:124	132:132	195:195	145:145	205:205	251:251	216:216	Admixed
MLG457	4	CHE242, CHE244, CHE245, CHE246	CHE (4)	Leaf (4)	HDP (4)	124:130	132:132	195:195	143:147	202:205	251:251	213:219	Admixed
MLG458	1	CHE243	CHE (1)	Leaf (1)	HDP (1)	115:130	132:132	195:195	143:147	202:205	251:251	213:219	Admixed
MLG459	1	CHE247	CHE (1)	Root (1)	Rts (1)	115:130	132:132	195:195	143:143	202:205	251:253	219:222	Admixed
MLG460	1	CHE248	CHE (1)	Root (1)	Rts (1)	115:130	132:132	195:195	143:147	202:205	251:251	222:222	Admixed
MLG461	2	CHE249, CHE251	CHE (2)	Root (2)	Rts (2)	115:130	132:132	195:195	143:147	202:202	251:251	213:213	Eu2
MLG462	1	CHE250	CHE (1)	Root (1)	Rts (1)	124:130	132:132	195:195	143:143	202:202	241:251	213:219	Admixed
MLG463	5	CHE252, CHE253, CHE254, CHE255, CHE258	CHE (5)	Leaf (4), Root (1)	HDP (5)	121:130	132:132	195:195	143:147	202:202	245:251	222:225	Admixed
MLG464	1	CHE256	CHE (1)	Leaf (1)	HDP (1)	130:130	132:132	195:195	145:145	202:202	245:251	222:225	Admixed
MLG465	2	CHE257, CHE259	CHE (2)	Root (2)	HDP (2)	124:130	132:132	195:195	143:147	202:202	241:251	222:222	Admixed
MLG466	1	CHE260	CHE (1)	Root (1)	HDP (1)	124:124	132:132	195:195	145:147	196:205	247:247	210:228	Eu1
MLG467	1	CHE261	CHE (1)	Root (1)	HDP (1)	121:124	132:132	195:195	145:147	202:202	251:253	219:222	Admixed
MLG468	1	CHE262	CHE (1)	Leaf (1)	HDP (1)	130:130	132:132	195:201	145:147	208:208	245:251	222:222	Admixed
MLG469	8	CHE263, CHE264, CHE265, CHE266, CHE267, CHE268, CHE269, CHE270	CHE (8)	Leaf (4), Root (4)	HDP (4), Rts (4)	121:127	132:132	195:201	145:147	208:208	245:251	222:222	Admixed
MLG470	1	CHE271	CHE (1)	Leaf (1)	HDP (1)	121:130	132:132	195:195	145:145	208:208	251:251	222:228	Eu2

MLG	N	Sample(s) Code[1]	Country[2]	Feeding form	Host plant[3]	PhyIII_55	PhyIII_30	PhyIII_36	DV8	Dvit6	DVSSR4	DV4	Genetic group (K=2)
MLG471	2	CHE272, CHE273	CHE (2)	Leaf (2)	HDP (2)	130:130	132:132	195:213	145:145	208:208	251:251	222:222	Admixed
MLG472	1	CHE274	CHE (1)	Leaf (1)	HDP (1)	127:130	132:135	195:213	143:145	202:202	251:251	222:228	Eu2
MLG473	1	CHE275	CHE (1)	Leaf (1)	HDP (1)	130:130	135:135	195:195	143:143	205:208	251:251	222:222	Eu2
MLG474	1	CHE276	CHE (1)	Root (1)	HDP (1)	124:130	132:132	195:195	143:145	205:208	251:251	222:222	Admixed
MLG475	3	CHE277, CHE280, HUN023	CHE (2), HUN (1)	Leaf (3)	HDP (2), Rts (1)	127:130	132:132	195:195	145:145	202:208	251:251	222:222	Admixed
MLG476	1	CHE278	CHE (1)	Leaf (1)	HDP (1)	121:127	132:132	195:195	145:145	205:208	251:253	222:222	Admixed
MLG477	1	CHE279	CHE (1)	Leaf (1)	HDP (1)	130:130	132:135	195:195	143:145	208:208	245:251	216:222	Eu2
MLG478	2	CHE281, CHE283	CHE (2)	Leaf (2)	Rts (2)	115:121	129:132	195:195	143:147	202:208	251:251	222:222	Eu2
MLG479	1	CHE282	CHE (1)	Leaf (1)	Rts (1)	115:121	129:132	201:201	143:147	202:208	251:251	222:222	Admixed
MLG480	1	URY001	URY (1)	Leaf (1)	Vvin (1)	124:130	129:130	207:210	145:145	205:205	253:253	216:222	Eu1
MLG481	1	URY002	URY (1)	Leaf (1)	Vvin (1)	130:130	132:132	195:201	145:145	205:205	253:253	216:222	Eu1
MLG482	1	URY003	URY (1)	Leaf (1)	Vvin (1)	130:130	132:132	195:201	143:143	205:205	245:245	216:219	Eu1
MLG483	1	URY004	URY (1)	Leaf (1)	Vvin (1)	124:130	129:132	207:210	145:145	205:205	253:253	216:222	Eu1
MLG484	3	URY005, URY006, URY007	URY (3)	Leaf (3)	Vvin (3)	124:124	132:132	195:207	143:145	202:205	251:253	216:222	Eu1
MLG485	1	URY008	URY (1)	Leaf (1)	Vvin (1)	124:130	132:132	195:201	143:143	205:205	245:251	210:216	Eu1
MLG486	1	URY009	URY (1)	Leaf (1)	Vvin (1)	124:124	132:132	195:201	143:143	202:205	241:253	210:219	Eu1
MLG487	2	URY010, URY011	URY (2)	Leaf (2)	Vvin (2)	124:127	132:132	195:207	143:143	202:205	251:253	222:222	Eu1
MLG488	2	URY012, URY014	URY (2)	Leaf (2)	Vvin (2)	124:124	132:132	195:207	143:145	205:205	245:253	210:216	Eu1
MLG489	1	URY013	URY (1)	Leaf (1)	Vvin (1)	127:127	132:135	195:195	143:143	202:205	253:253	216:216	Admixed
MLG490	4	URY015, URY017, URY018, URY019	URY (4)	Leaf (4)	Vvin (4)	124:124	132:132	207:210	145:145	202:205	251:253	210:216	Eu1

MLG	N	Sample(s) Code[1]	Country[2]	Feeding form	Host plant[3]	PhyIII_55	PhyIII_30	PhyIII_36	DV8	Dvit6	DVSSR4	DV4	Genetic group (K=2)
MLG491	1	URY016	URY (1)	Leaf (1)	Vvin (1)	124:130	132:132	195:195	143:147	202:205	245:253	216:222	Admixed
MLG492	2	URY020, URY021	URY (2)	Leaf (2)	Vvin (2)	130:130	129:132	195:201	143:147	205:208	251:253	219:222	Admixed
MLG493	2	URY022, URY023	URY (2)	Leaf (2)	Vvin (2)	124:127	132:132	195:210	143:147	196:196	253:253	216:222	Eu1
MLG494	1	URY024	URY (1)	Leaf (1)	Vvin (1)	124:127	132:132	195:210	143:149	196:196	253:253	216:222	Eu1
MLG495	2	URY025, URY026	URY (2)	Leaf (2)	Vvin (2)	124:124	132:132	195:207	143:143	205:208	241:253	210:219	Eu1
MLG496	1	URY027	URY (1)	Leaf (1)	Vvin (1)	124:127	132:132	207:210	143:145	202:205	251:253	216:216	Eu1
MLG497	1	URY028	URY (1)	Leaf (1)	Vvin (1)	124:127	132:132	195:195	145:147	205:205	253:253	210:219	Eu1
MLG498	1	URY029	URY (1)	Leaf (1)	Vvin (1)	124:130	132:132	195:195	147:147	205:205	245:253	210:216	Eu1
MLG499	5	URY030, URY031, URY032, URY033, URY034	URY (5)	Leaf (5)	Vvin (5)	124:127	132:132	195:210	143:147	205:205	253:253	210:216	Eu1
MLG500	2	URY035, URY036	URY (2)	Leaf (2)	Vvin (2)	124:130	132:132	195:195	143:143	205:205	253:253	210:216	Eu1
MLG501	1	URY037	URY (1)	Leaf (1)	Vvin (1)	124:130	132:132	195:195	143:145	202:205	251:253	216:216	Admixed
MLG502	1	URY038	URY (1)	Leaf (1)	Vvin (1)	124:127	132:132	195:195	143:145	202:205	251:251	216:219	Admixed
MLG503	2	URY039, URY040	URY (2)	Leaf (2)	Vvin (2)	127:130	129:132	195:195	143:143	205:208	253:253	216:219	Admixed
MLG504	3	URY041, URY043, URY044	URY (3)	Leaf (3)	Vvin (3)	130:130	129:132	195:198	143:143	205:205	241:253	219:219	Admixed
MLG505	1	URY042	URY (1)	Leaf (1)	Vvin (1)	-:-	132:132	195:195	143:145	205:205	253:253	216:219	Eu1
MLG506	1	SRB001	SRB (1)	Leaf (1)	RGV (1)	130:130	132:135	195:204	143:149	205:208	-:-	219:219	Admixed
MLG507	1	SRB002	SRB (1)	Leaf (1)	RGV (1)	124:130	132:141	195:195	143:143	205:205	-:-	216:225	Admixed
MLG508	1	SRB003	SRB (1)	Leaf (1)	RGV (1)	124:130	132:141	195:198	145:145	-:-	-:-	-:-	Admixed
MLG509	1	SRB004	SRB (1)	Root (1)	RGV (1)	124:127	132:141	195:195	143:145	205:208	251:251	210:222	Admixed
MLG510	1	SRB005	SRB (1)	Leaf (1)	RGV (1)	127:130	129:141	195:195	145:145	202:205	251:251	222:222	Admixed

MLG	N	Sample(s) Code[1]	Country[2]	Feeding form	Host plant[3]	PhyIII_55	PhyIII_30	PhyIII_36	DV8	Dvit6	DVSSR4	DV4	Genetic group (K=2)
MLG511	1	SRB006	SRB (1)	Leaf (1)	RGV (1)	127:130	132:132	195:195	143:143	202:208	251:251	216:222	Admixed
MLG512	1	SRB007	SRB (1)	Leaf (1)	RGV (1)	124:127	129:141	195:198	-:-	202:205	-:-	-:-	Admixed
MLG513	1	SRB008	SRB (1)	Leaf (1)	RGV (1)	124:130	132:135	195:198	143:145	202:202	241:241	219:228	Admixed
MLG514	1	SRB009	SRB (1)	Leaf (1)	RGV (1)	127:130	129:141	195:195	145:145	202:205	251:251	210:222	Admixed
MLG515	2	SRB010, SRB011	SRB (2)	Leaf (2)	RGV (2)	124:130	132:135	195:204	143:145	205:205	251:251	210:216	Admixed
MLG516	1	SRB012	SRB (1)	Root (1)	Rts (1)	-:-	132:135	195:198	145:145	202:202	251:251	219:219	Eu2
MLG517	1	SRB013	SRB (1)	Root (1)	Rts (1)	124:127	129:132	195:195	143:145	202:205	-:-	216:222	Admixed
MLG518	1	SRB014	SRB (1)	Leaf (1)	Rts (1)	124:136	123:132	195:198	143:145	-:-	-:-	216:216	Admixed
MLG519	1	SRB015	SRB (1)	Leaf (1)	Rts (1)	118:124	123:123	204:204	-:-	208:208	-:-	-:-	Admixed
MLG520	1	SRB016	SRB (1)	Root (1)	Rts (1)	127:127	132:132	195:195	143:145	202:202	251:251	219:219	Admixed
MLG521	3	SRB017, SRB023, SRB025	SRB (3)	Leaf (2), Root(1)	Rts (3)	130:130	129:132	195:195	143:145	202:208	251:253	216:219	Eu2
MLG522	1	SRB018	SRB (1)	Root (1)	Rts (1)	127:130	129:132	-:-	145:147	208:208	251:251	210:216	Admixed
MLG523	1	SRB019	SRB (1)	Root (1)	Rts (1)	127:130	129:132	195:195	145:147	208:208	251:251	210:216	Admixed
MLG524	3	SRB020, SRB021, SRB022	SRB (3)	Root (3)	Rts (3)	130:130	132:135	195:204	143:145	205:208	241:251	216:219	Admixed
MLG525	1	SRB024	SRB (1)	Leaf (1)	Rts (1)	124:130	129:132	-:-	143:145	202:208	-:-	-:-	Admixed
MLG526	2	SRB026, SRB027	SRB (2)	Leaf (2)	Rts (2)	127:130	129:132	195:195	143:145	202:205	241:251	219:228	Admixed
MLG527	1	SRB028	SRB (1)	Leaf (1)	Rts (1)	124:130	129:129	195:195	145:145	202:202	251:251	219:222	Eu2
MLG528	1	SRB029	SRB (1)	Leaf (1)	Rts (1)	124:130	129:129	195:195	145:145	202:202	-:-	219:219	Eu2
MLG529	1	SRB030	SRB (1)	Leaf (1)	Rts (1)	-:-	129:129	195:195	145:145	202:202	251:251	219:228	Eu2
MLG530	1	SRB031	SRB (1)	Leaf (1)	Vvin (1)	127:130	132:132	195:204	145:145	208:208	241:251	219:219	Admixed
MLG531	1	SRB032	SRB (1)	Leaf (1)	Vvin (1)	124:130	129:135	195:204	143:145	205:208	251:251	219:222	Eu2
MLG532	1	SRB033	SRB (1)	Leaf (1)	Vvin (1)	124:127	132:132	-:-	143:143	202:208	251:251	219:222	Admixed

MLG	N	Sample(s) Code[1]	Country[2]	Feeding form	Host plant[3]	PhyIII_55	PhyIII_30	PhyIII_36	DV8	Dvit6	DVSSR4	DV4	Genetic group (K=2)
MLG533	2	SRB034, SRB035	SRB (2)	Leaf (2)	Vvin (2)	130:130	129:132	195:195	143:145	202:208	-:-	216:219	Eu2
MLG534	1	SRB036	SRB (1)	Leaf (1)	RGV (1)	124:127	132:132	195:195	145:145	202:202	-:-	222:222	Admixed
MLG535	1	SRB037	SRB (1)	Leaf (1)	RGV (1)	124:127	132:141	195:195	145:145	202:208	241:251	222:222	Admixed
MLG536	1	SRB038	SRB (1)	Root (1)	RGV (1)	130:130	132:135	195:195	143:143	208:208	251:251	219:219	Eu2
MLG537	1	SRB039	SRB (1)	Root (1)	Vvin (1)	130:130	132:135	195:198	145:147	202:208	-:-	219:222	Eu2
MLG538	1	ITA001	ITA (1)	Leaf (1)	Rts (1)	115:130	132:132	195:198	143:143	202:202	241:251	210:216	Admixed
MLG539	2	ITA002, ITA045	ITA (2)	Leaf (2)	Rts (2)	115:130	132:135	195:195	143:145	202:205	251:253	210:216	Admixed
MLG540	1	ITA003	ITA (1)	Leaf (1)	Rts (1)	124:130	132:132	195:198	145:145	202:205	245:251	210:222	Admixed
MLG541	2	ITA004, ITA006	ITA (2)	Leaf (2)	Rts (2)	130:130	132:132	195:198	143:145	202:205	251:251	222:222	Admixed
MLG542	1	ITA005	ITA (1)	Leaf (1)	Rts (1)	127:130	132:132	195:204	143:145	202:202	251:253	210:210	Eu1
MLG543	1	ITA007	ITA (1)	Leaf (1)	Rts (1)	130:130	132:132	195:195	143:145	202:205	251:253	210:210	Eu1
MLG544	2	ITA008, ITA010	ITA (2)	Leaf (2)	Rts (2)	124:124	132:132	195:195	143:145	202:202	241:251	219:219	Admixed
MLG545	1	ITA009	ITA (1)	Leaf (1)	Rts (1)	124:127	129:132	195:195	143:143	196:205	241:253	210:210	Eu1
MLG546	1	ITA011	ITA (1)	Leaf (1)	Rts (1)	124:130	132:132	195:195	143:143	202:208	251:253	210:219	Admixed
MLG547	1	ITA012	ITA (1)	Leaf (1)	Vvin (1)	124:136	132:132	195:195	143:145	-:-	-:-	216:225	Admixed
MLG548	1	ITA013	ITA (1)	Leaf (1)	Vvin (1)	130:130	132:132	195:195	143:143	202:208	251:253	210:210	Admixed
MLG549	2	ITA014, ITA015	ITA (2)	Leaf (2)	Rts (2)	130:130	129:132	195:198	143:143	205:205	251:253	210:216	Admixed
MLG550	1	ITA016	ITA (1)	Leaf (1)	Rts (1)	124:127	129:132	195:195	143:143	202:208	251:253	210:216	Admixed
MLG551	1	ITA017	ITA (1)	Leaf (1)	Vvin (1)	130:130	132:132	195:207	143:145	205:205	251:253	222:228	Admixed
MLG552	1	ITA018	ITA (1)	Leaf (1)	Vvin (1)	130:130	132:132	195:195	143:145	205:205	253:255	222:228	Admixed
MLG553	1	ITA019	ITA (1)	Leaf (1)	Vvin (1)	124:124	132:132	195:198	145:147	205:205	251:251	216:222	Admixed
MLG554	1	ITA020	ITA (1)	Leaf (1)	Vvin (1)	124:124	132:132	195:198	143:145	205:205	251:251	216:222	Admixed
MLG555	1	ITA021	ITA (1)	Leaf (1)	Vvin (1)	130:133	132:132	195:204	143:143	202:208	245:251	216:222	Admixed

MLG	N	Sample(s) Code[1]	Country[2]	Feeding form	Host plant[3]	PhyIII_55	PhyIII_30	PhyIII_36	DV8	Dvit6	DVSSR4	DV4	Genetic group (K=2)
MLG556	3	ITA022, ITA025, ITA026	ITA (3)	Leaf (3)	Rts (3)	124:124	132:132	195:195	143:145	202:202	251:251	210:216	Admixed
MLG557	3	ITA023, ITA024, ITA032	ITA (3)	Leaf (3)	Rts (3)	130:130	132:135	195:195	143:143	202:208	251:253	210:222	Admixed
MLG558	4	ITA027, ITA029, ITA030, ITA031	ITA (4)	Leaf (4)	Rts (4)	124:124	132:132	195:195	143:145	202:202	251:251	210:222	Eu1
MLG559	1	ITA028	ITA (1)	Leaf (1)	Rts (1)	124:124	132:132	195:195	143:147	202:202	251:251	210:222	Admixed
MLG560	3	ITA033, ITA035, ITA036	ITA (3)	Leaf (3)	Rts (3)	124:124	132:132	195:195	143:145	205:205	253:253	210:210	Eu1
MLG561	1	ITA034	ITA (1)	Leaf (1)	Rts (1)	115:127	132:135	195:195	145:145	202:208	245:253	210:216	Admixed
MLG562	3	ITA037, ITA038, ITA041	ITA (3)	Leaf (3)	Rts (3)	124:127	132:132	195:195	143:143	202:205	251:251	210:219	Eu1
MLG563	2	ITA039, ITA040	ITA (2)	Leaf (2)	Rts (2)	124:127	132:132	195:195	145:145	202:205	251:251	210:219	Eu1
MLG564	1	ITA042	ITA (1)	Leaf (1)	Rts (1)	130:130	129:132	192:195	143:143	202:202	251:253	210:210	Admixed
MLG565	2	ITA043, ITA044	ITA (2)	Leaf (2)	Rts (2)	130:130	129:132	195:195	143:143	202:202	251:253	210:210	Admixed
MLG566	1	ITA046	ITA (1)	Leaf (1)	Rts (1)	115:130	132:135	198:198	143:143	202:202	241:251	210:216	Eu2
MLG567	1	ITA047	ITA (1)	Leaf (1)	Rts (1)	115:130	132:132	195:198	145:145	202:202	241:251	210:216	Admixed
MLG568	1	ITA048	ITA (1)	Leaf (1)	Rts (1)	124:130	132:135	195:195	147:147	205:208	249:251	210:222	Admixed
MLG569	1	ITA049	ITA (1)	Leaf (1)	Rts (1)	124:130	132:135	195:195	145:145	205:208	251:253	210:222	Admixed
MLG570	1	ITA050	ITA (1)	Leaf (1)	Rts (1)	127:130	120:135	195:195	145:145	202:208	251:251	219:219	Admixed
MLG571	1	ITA051	ITA (1)	Leaf (1)	Rts (1)	121:130	132:135	195:195	145:145	205:208	251:253	210:222	Admixed
MLG572	1	ITA052	ITA (1)	Leaf (1)	Rts (1)	124:130	132:135	195:195	145:145	205:208	249:251	210:222	Admixed
MLG573	1	ITA053	ITA (1)	Leaf (1)	Vvin (1)	124:133	132:132	195:195	143:145	208:208	253:253	216:222	Admixed

MLG	N	Sample(s) Code[1]	Country[2]	Feeding form	Host plant[3]	PhyIII_55	PhyIII_30	PhyIII_36	DV8	Dvit6	DVSSR4	DV4	Genetic group (K=2)
MLG574	1	ITA054	ITA (1)	Leaf (1)	Vvin (1)	130:130	129:132	195:207	147:147	202:208	253:253	219:222	Admixed
MLG575	1	ITA055	ITA (1)	Leaf (1)	Vvin (1)	130:130	132:132	195:207	143:143	205:208	245:253	222:228	Admixed
MLG576	1	ITA056	ITA (1)	Leaf (1)	Vvin (1)	124:130	132:135	195:207	147:147	205:208	251:253	219:219	Admixed
MLG577	1	ITA057	ITA (1)	Leaf (1)	Vvin (1)	121:127	132:132	195:195	143:145	205:205	245:251	222:222	Eu1
MLG578	1	ITA058	ITA (1)	Leaf (1)	Vvin (1)	127:130	129:135	195:204	143:145	202:202	251:251	216:216	Eu2
MLG579	1	ITA059	ITA (1)	Leaf (1)	Vvin (1)	124:130	129:132	195:207	143:147	208:208	245:251	222:222	Admixed
MLG580	1	ITA060	ITA (1)	Leaf (1)	Vvin (1)	124:124	132:132	204:207	143:143	202:202	245:251	222:222	Eu1
MLG581	1	ITA061	ITA (1)	Leaf (1)	Vvin (1)	124:130	132:132	195:207	147:147	208:208	251:251	219:222	Admixed
MLG582	1	ITA062	ITA (1)	Leaf (1)	Vvin (1)	127:130	132:132	195:195	147:147	208:208	251:253	216:222	Admixed
MLG583	1	ITA063	ITA (1)	Leaf (1)	Vvin (1)	127:130	129:132	195:207	143:143	202:202	251:251	216:222	Admixed
MLG584	1	ITA064	ITA (1)	Leaf (1)	Vvin (1)	127:130	132:132	195:207	143:143	208:208	251:251	216:216	Admixed
MLG585	1	ITA065	ITA (1)	Leaf (1)	Vvin (1)	130:130	132:132	195:207	145:145	202:202	245:251	210:216	Admixed
MLG586	1	ITA066	ITA (1)	Leaf (1)	Vvin (1)	124:130	132:132	195:195	143:145	202:208	245:251	210:222	Admixed
MLG587	1	ITA067	ITA (1)	Leaf (1)	Vvin (1)	124:130	132:132	195:207	145:147	208:208	251:251	210:222	Admixed
MLG588	1	ITA068	ITA (1)	Leaf (1)	Vvin (1)	130:130	135:141	195:198	145:145	202:205	251:251	216:222	Eu2
MLG589	1	ITA069	ITA (1)	Leaf (1)	Vvin (1)	127:130	132:141	195:195	143:143	205:208	251:251	216:216	Admixed
MLG590	1	ITA070	ITA (1)	Leaf (1)	Vvin (1)	124:130	135:135	195:195	145:145	205:208	251:253	216:219	Eu2
MLG591	1	ITA071	ITA (1)	Leaf (1)	Vvin (1)	130:130	132:132	195:207	147:147	208:208	251:251	210:222	Admixed
MLG592	1	ITA072	ITA (1)	Leaf (1)	Vvin (1)	130:130	132:132	195:207	143:145	205:205	251:251	210:210	Eu1
MLG593	1	ITA073	ITA (1)	Leaf (1)	Vvin (1)	124:127	132:132	195:207	143:145	205:208	245:251	210:222	Eu1
MLG594	1	ITA074	ITA (1)	Leaf (1)	Vvin (1)	130:133	132:141	195:195	145:145	202:208	245:245	222:228	Eu2
MLG595	1	ITA075	ITA (1)	Leaf (1)	Vvin (1)	130:130	132:132	207:207	145:145	202:202	251:251	210:219	Eu1
MLG596	1	ITA076	ITA (1)	Leaf (1)	Vvin (1)	124:130	132:132	195:207	143:145	196:202	251:251	210:222	Eu1
MLG597	1	ITA077	ITA (1)	Leaf (1)	Vvin (1)	130:133	132:132	195:195	143:145	202:208	245:253	219:222	Admixed
MLG598	1	ITA078	ITA (1)	Leaf (1)	Vvin (1)	130:130	132:132	195:195	143:145	205:208	251:251	210:222	Admixed
MLG599	1	ITA079	ITA (1)	Leaf (1)	Vvin (1)	130:130	132:132	195:195	143:143	205:205	251:253	210:228	Admixed

MLG	N	Sample(s) Code[1]	Country[2]	Feeding form	Host plant[3]	PhyIII_55	PhyIII_30	PhyIII_36	DV8	Dvit6	DVSSR4	DV4	Genetic group (K=2)
MLG600	7	ZAF001, ZAF002, ZAF003, ZAF004, ZAF005, ZAF006, ZAF007	ZAF (7)	Leaf (7)	Rts (7)	127:136	123:132	192:195	145:151	202:205	251:251	216:225	Admixed
MLG601	1	ZAF008	ZAF (1)	Leaf (1)	Rts (1)	127:127	123:132	192:195	145:151	202:205	251:251	216:225	Admixed
MLG602	1	HUN001	HUN (1)	Leaf (1)	Rts (1)	124:130	129:132	195:195	143:145	202:202	219:222	216:216	Admixed
MLG603	1	HUN002	HUN (1)	Leaf (1)	Rts (1)	127:130	129:129	195:195	145:145	202:208	251:251	216:222	Eu2
MLG604	1	HUN003	HUN (1)	Leaf (1)	Rts (1)	127:127	132:132	195:195	145:145	202:202	251:251	216:222	Admixed
MLG605	1	HUN004	HUN (1)	Leaf (1)	Rts (1)	127:130	132:132	195:204	145:147	202:208	251:251	210:222	Admixed
MLG606	1	HUN005	HUN (1)	Leaf (1)	Rts (1)	124:130	132:135	195:195	145:145	202:202	251:251	216:216	Eu2
MLG607	1	HUN006	HUN (1)	Leaf (1)	Rts (1)	124:124	132:132	195:204	143:145	202:208	251:251	216:219	Admixed
MLG608	1	HUN007	HUN (1)	Root (1)	Rts (1)	124:130	129:135	195:198	143:147	202:208	251:251	222:222	Eu2
MLG609	1	HUN008	HUN (1)	Leaf (1)	Rts (1)	124:127	129:132	195:195	143:147	202:202	251:251	216:222	Admixed
MLG610	1	HUN009	HUN (1)	Leaf (1)	Rts (1)	127:130	132:135	195:198	143:145	208:208	251:251	210:222	Eu2
MLG611	1	HUN010	HUN (1)	Leaf (1)	Rts (1)	124:130	123:132	195:204	143:145	205:205	241:251	210:210	Eu1
MLG612	1	HUN011	HUN (1)	Root (1)	Rts (1)	124:124	129:132	195:222	143:145	205:208	251:251	219:219	Admixed
MLG613	1	HUN012	HUN (1)	Root (1)	Rts (1)	124:124	129:132	195:222	143:145	205:208	251:251	210:219	Admixed
MLG614	1	HUN013	HUN (1)	Root (1)	Rts (1)	130:130	132:132	195:195	143:145	205:205	251:251	210:222	Eu1
MLG615	1	HUN014	HUN (1)	Leaf (1)	Rts (1)	124:130	132:132	195:222	143:145	202:208	251:251	216:222	Admixed
MLG616	1	HUN015	HUN (1)	Leaf (1)	Rts (1)	124:124	129:132	195:195	145:145	202:202	245:251	222:228	Admixed
MLG617	1	HUN016	HUN (1)	Leaf (1)	Rts (1)	127:130	129:132	195:195	143:145	202:202	251:251	210:222	Admixed
MLG618	1	HUN017	HUN (1)	Root (1)	Rts (1)	127:130	132:132	195:195	145:147	202:208	251:251	222:228	Admixed
MLG619	1	HUN018	HUN (1)	Root (1)	Rts (1)	130:130	132:132	204:204	145:147	202:202	251:251	210:216	Admixed
MLG620	1	HUN019	HUN (1)	Root (1)	Rts (1)	127:130	129:132	195:195	143:143	202:202	251:251	210:222	Admixed
MLG621	1	HUN020	HUN (1)	Root (1)	Rts (1)	127:130	132:132	195:195	145:145	202:208	245:251	222:222	Admixed

MLG	N	Sample(s) Code[1]	Country[2]	Feeding form	Host plant[3]	PhyIII_55	PhyIII_30	PhyIII_36	DV8	Dvit6	DVSSR4	DV4	Genetic group (K=2)
MLG622	1	HUN021	HUN (1)	Leaf (1)	Rts (1)	127:130	132:132	195:195	145:145	202:208	245:251	219:222	Admixed
MLG623	1	HUN022	HUN (1)	Leaf (1)	Rts (1)	130:130	129:132	195:195	145:147	202:202	251:251	222:222	Eu2
MLG624	1	HUN024	HUN (1)	Leaf (1)	Rts (1)	127:130	129:132	195:195	143:145	205:205	245:251	210:210	Eu1
MLG625	1	HUN025	HUN (1)	Root (1)	Rts (1)	127:130	129:135	195:195	145:147	196:202	251:251	219:222	Admixed
MLG626	1	HUN026	HUN (1)	Root (1)	Rts (1)	130:130	129:132	195:198	145:147	202:208	245:245	216:222	Eu2
MLG627	1	HUN027	HUN (1)	Root (1)	Rts (1)	124:130	132:132	195:195	145:145	196:208	251:251	219:222	Admixed
MLG628	1	HUN028	HUN (1)	Leaf (1)	Rts (1)	124:127	132:132	204:213	143:147	202:205	251:251	210:216	Eu1
MLG629	1	HUN029	HUN (1)	Leaf (1)	Rts (1)	115:130	129:132	195:195	145:145	202:202	251:251	222:222	Eu2
MLG630	1	HUN030	HUN (1)	Leaf (1)	Rts (1)	130:130	129:132	195:198	143:143	202:208	251:251	216:219	Eu2
MLG631	1	HUN031	HUN (1)	Leaf (1)	Rts (1)	124:130	129:132	195:198	145:147	205:205	251:251	219:228	Admixed
MLG632	1	HUN032	HUN (1)	Leaf (1)	Rts (1)	130:130	129:129	195:195	145:145	202:205	251:253	219:222	Eu2
MLG633	1	HUN033	HUN (1)	Root (1)	Rts (1)	124:127	129:132	195:195	143:143	205:208	245:251	213:219	Admixed
MLG634	1	HUN034	HUN (1)	Root (1)	Rts (1)	124:124	129:141	195:195	145:145	202:208	251:251	219:219	Admixed
MLG635	1	HUN035	HUN (1)	Leaf (1)	Rts (1)	130:130	129:129	195:204	145:145	205:205	251:253	213:216	Admixed
MLG636	1	HUN036	HUN (1)	Leaf (1)	Rts (1)	127:130	132:132	195:198	143:145	205:208	241:251	210:216	Admixed
MLG637	1	HUN037	HUN (1)	Leaf (1)	Rts (1)	127:130	132:132	195:198	143:145	205:208	251:251	213:216	Admixed
MLG638	1	HUN038	HUN (1)	Leaf (1)	Rts (1)	127:130	129:132	195:195	143:143	202:208	245:251	216:216	Admixed
MLG639	1	HUN039	HUN (1)	Leaf (1)	Rts (1)	130:130	129:132	195:195	143:145	202:208	241:251	210:219	Admixed
MLG640	1	HUN040	HUN (1)	Leaf (1)	Rts (1)	130:130	129:129	195:195	143:145	202:208	241:241	210:219	Admixed
MLG641	1	HUN041	HUN (1)	Root (1)	Rts (1)	130:130	132:132	195:204	145:145	208:208	245:251	210:222	Admixed
MLG642	1	HUN042	HUN (1)	Root (1)	Rts (1)	130:130	129:135	204:204	145:145	208:208	245:251	216:222	Eu2
MLG643	1	HUN043	HUN (1)	Root (1)	Rts (1)	127:130	129:135	195:195	145:147	202:202	245:251	210:222	Eu2
MLG644	1	HUN044	HUN (1)	Root (1)	Rts (1)	124:130	132:135	195:198	143:145	205:208	245:251	210:216	Admixed
MLG645	1	HUN045	HUN (1)	Root (1)	Rts (1)	130:130	132:132	195:204	145:145	202:208	251:251	210:222	Admixed
MLG646	1	HUN046	HUN (1)	Leaf (1)	Rts (1)	130:130	129:132	195:195	145:145	205:208	251:251	216:222	Admixed
MLG647	1	HUN047	HUN (1)	Leaf (1)	Rts (1)	130:130	129:132	195:195	145:145	208:208	251:251	216:222	Eu2

MLG	N	Sample(s) Code[1]	Country[2]	Feeding form	Host plant[3]	PhyIII_55	PhyIII_30	PhyIII_36	DV8	Dvit6	DVSSR4	DV4	Genetic group (K=2)
MLG648	1	HUN048	HUN (1)	Root (1)	Rts (1)	127:130	129:129	195:195	143:145	202:202	251:251	210:210	Admixed
MLG649	1	HUN049	HUN (1)	Root (1)	Rts (1)	130:130	129:135	195:207	145:147	202:202	251:251	210:210	Admixed
MLG650	1	HUN050	HUN (1)	Leaf (1)	Rts (1)	130:103	129:135	195:207	145:147	202:202	251:251	210:210	Admixed
MLG651	1	HUN051	HUN (1)	Leaf (1)	Rts (1)	124:130	129:132	195:204	143:143	202:202	245:251	216:222	Admixed
MLG652	1	HUN052	HUN (1)	Leaf (1)	Rts (1)	130:130	132:132	195:195	145:147	202:202	245:251	210:222	Admixed
MLG653	1	HUN053	HUN (1)	Leaf (1)	Rts (1)	124:130	132:132	195:195	145:145	202:208	251:251	222:222	Admixed
MLG654	1	HUN054	HUN (1)	Leaf (1)	Rts (1)	124:130	132:132	195:195	145:145	202:208	245:251	222:222	Admixed
MLG655	1	HUN055	HUN (1)	Leaf (1)	Rts (1)	130:130	132:132	204:204	143:143	208:208	241:241	210:210	Eu1
MLG656	1	HUN056	HUN (1)	Leaf (1)	Rts (1)	130:130	132:132	195:195	143:145	202:208	251:251	216:219	Admixed
MLG657	1	HUN057	HUN (1)	Leaf (1)	Rts (1)	124:130	129:132	195:204	143:145	202:208	241:251	216:225	Admixed
MLG658	1	HUN058	HUN (1)	Leaf (1)	Rts (1)	127:130	132:132	195:204	145:147	202:208	251:251	219:228	Admixed
MLG659	1	HUN059	HUN (1)	Root (1)	Rts (1)	124:124	132:132	195:204	145:145	202:208	251:251	219:222	Admixed
MLG660	1	HUN060	HUN (1)	Root (1)	Rts (1)	121:130	132:132	195:204	145:145	202:202	251:251	222:222	Admixed
MLG661	1	HUN061	HUN (1)	Root (1)	Rts (1)	124:130	132:132	195:195	145:145	202:205	251:253	216:222	Admixed
MLG662	1	HUN062	HUN (1)	Root (1)	Rts (1)	124:133	132:132	195:195	143:147	208:208	249:251	210:210	Admixed
MLG663	1	HUN063	HUN (1)	Root (1)	Rts (1)	127:130	129:132	195:195	145:145	202:205	251:251	216:216	Admixed
MLG664	1	HUN064	HUN (1)	Leaf (1)	Rts (1)	124:130	129:132	195:204	145:145	202:205	251:251	216:228	Admixed
MLG665	1	HUN065	HUN (1)	Root (1)	Rts (1)	127:130	129:132	195:195	145:147	208:208	251:251	210:225	Admixed
MLG666	1	HUN066	HUN (1)	Root (1)	Rts (1)	127:127	132:135	195:195	143:145	202:202	251:253	213:219	Admixed
MLG667	1	HUN067	HUN (1)	Root (1)	Rts (1)	124:127	132:132	195:195	143:145	202:208	253:253	222:228	Admixed
MLG668	1	HUN068	HUN (1)	Root (1)	Rts (1)	124:130	132:132	195:195	143:143	202:208	251:251	216:222	Admixed
MLG669	1	HUN069	HUN (1)	Root (1)	Rts (1)	127:127	126:132	195:222	145:145	202:205	253:253	222:222	Eu1
MLG670	1	HUN070	HUN (1)	Leaf (1)	Rts (1)	118:127	132:132	195:195	145:145	202:202	251:253	219:222	Admixed
MLG671	1	HUN071	HUN (1)	Leaf (1)	Rts (1)	127:130	132:132	195:195	143:145	202:202	253:253	222:222	Admixed
MLG672	1	HUN072	HUN (1)	Leaf (1)	Rts (1)	130:130	132:132	195:195	143:145	202:202	253:253	222:222	Admixed
MLG673	1	HUN073	HUN (1)	Leaf (1)	Rts (1)	124:130	132:132	195:195	143:145	208:208	241:251	213:219	Admixed

MLG	N	Sample(s) Code[1]	Country[2]	Feeding form	Host plant[3]	PhyIII_55	PhyIII_30	PhyIII_36	DV8	Dvit6	DVSSR4	DV4	Genetic group (K=2)
MLG674	1	HUN074	HUN (1)	Leaf (1)	Rts (1)	124:130	132:132	195:195	143:147	202:202	251:251	216:222	Admixed
MLG675	1	HUN075	HUN (1)	Leaf (1)	Rts (1)	124:127	132:132	195:222	143:145	202:208	251:251	219:222	Admixed
MLG676	1	HUN076	HUN (1)	Leaf (1)	Rts (1)	130:130	132:132	195:204	143:145	205:205	251:253	210:222	Eu1
MLG677	1	HUN077	HUN (1)	Leaf (1)	Rts (1)	124:130	132:132	195:195	145:145	202:205	251:251	210:216	Admixed
MLG678	1	HUN078	HUN (1)	Root (1)	Rts (1)	124:127	132:132	195:195	145:147	208:208	251:253	222:222	Admixed
MLG679	1	HUN079	HUN (1)	Root (1)	Rts (1)	121:124	132:132	195:195	143:145	202:208	253:253	219:222	Admixed
MLG680	1	HUN080	HUN (1)	Root (1)	Rts (1)	124:127	132:132	195:195	145:147	202:208	251:253	222:222	Admixed
MLG681	1	HUN081	HUN (1)	Leaf (1)	Rts (1)	127:127	132:132	195:222	145:145	202:205	253:253	222:222	Eu1
MLG682	1	HUN082	HUN (1)	Root (1)	Rts (1)	124:127	123:132	195:222	143:145	202:208	251:253	219:222	Admixed
MLG683	1	HUN083	HUN (1)	Root (1)	Rts (1)	130:130	129:135	195:195	145:147	202:208	251:253	219:228	Eu2
MLG684	1	HUN084	HUN (1)	Root (1)	Rts (1)	124:124	132:141	195:210	143:145	205:208	251:251	219:219	Admixed
MLG685	1	HUN085	HUN (1)	Root (1)	Rts (1)	127:127	129:132	195:195	143:145	202:205	251:253	210:210	Eu1
MLG686	1	HUN086	HUN (1)	Root (1)	Rts (1)	127:127	132:132	195:195	143:145	196:202	251:251	210:216	Eu1
MLG687	1	HUN087	HUN (1)	Root (1)	Rts (1)	124:127	132:132	195:195	143:145	196:202	251:253	216:225	Eu1
MLG688	1	HUN088	HUN (1)	Root (1)	Rts (1)	124:127	132:132	195:195	143:145	196:202	251:251	210:210	Eu1
MLG689	1	HUN089	HUN (1)	Root (1)	Rts (1)	130:130	132:132	195:195	143:147	202:208	251:251	216:219	Eu2
MLG690	1	HUN090	HUN (1)	Root (1)	Rts (1)	121:124	132:135	195:195	143:143	202:208	245:251	216:216	Eu2
MLG691	1	HUN091	HUN (1)	Root (1)	Rts (1)	121:130	132:135	195:204	143:143	202:208	245:251	216:216	Eu2
MLG692	1	HUN092	HUN (1)	Root (1)	Rts (1)	130:133	129:135	195:195	143:143	202:208	251:251	219:219	Eu2
MLG693	1	HUN093	HUN (1)	Root (1)	Rts (1)	127:130	132:132	195:195	143:145	208:208	251:251	219:222	Admixed
MLG694	1	HUN094	HUN (1)	Root (1)	Rts (1)	124:133	129:132	195:195	145:145	202:208	251:253	219:219	Eu2
MLG695	1	HUN095	HUN (1)	Root (1)	Rts (1)	124:127	132:132	195:195	143:147	208:208	241:251	219:219	Admixed
MLG696	1	HUN096	HUN (1)	Root (1)	Rts (1)	127:127	132:141	195:195	143:143	205:205	245:251	210:228	Admixed
MLG697	1	HUN097	HUN (1)	Root (1)	Rts (1)	127:127	132:141	195:195	143:143	205:205	245:251	210:210	Eu1
MLG698	1	HUN098	HUN (1)	Root (1)	Rts (1)	127:127	132:141	195:195	143:143	205:205	245:251	225:228	Admixed
MLG699	1	HUN099	HUN (1)	Root (1)	Rts (1)	127:130	132:132	195:204	143:147	202:202	251:251	222:222	Admixed

MLG	N	Sample(s) Code[1]	Country[2]	Feeding form	Host plant[3]	PhyIII_55	PhyIII_30	PhyIII_36	DV8	Dvit6	DVSSR4	DV4	Genetic group (K=2)
MLG700	1	HUN100	HUN (1)	Root (1)	Rts (1)	127:130	132:132	195:204	143:147	202:202	251:251	219:222	Admixed
MLG701	1	HUN101	HUN (1)	Root (1)	Rts (1)	130:130	132:132	195:204	143:145	202:208	251:253	210:222	Admixed
MLG702	1	HUN102	HUN (1)	Leaf (1)	Rts (1)	127:127	132:132	195:222	145:145	202:202	253:253	222:222	Eu1
MLG703	1	HRV001	HRV (1)	Leaf (1)	n.a. (1)	127:130	132:132	195:207	143:147	202:208	251:251	210:222	Admixed
MLG704	1	HRV002	HRV (1)	Leaf (1)	n.a. (1)	124:130	129:132	195:195	145:145	208:208	251:253	216:216	Eu2
MLG705	1	HRV003	HRV (1)	Leaf (1)	n.a. (1)	127:130	129:135	195:204	143:143	202:202	251:251	216:219	Eu2
MLG706	1	HRV004	HRV (1)	Leaf (1)	n.a. (1)	124:130	129:132	195:195	145:145	208:208	251:253	216:228	Eu2
MLG707	1	HRV005	HRV (1)	Leaf (1)	n.a. (1)	124:127	132:132	195:204	145:145	202:202	251:251	222:222	Admixed
MLG708	1	HRV006	HRV (1)	Leaf (1)	n.a. (1)	127:130	132:132	195:204	145:145	202:205	251:251	216:219	Admixed
MLG709	2	HRV007, HRV009	HRV (2)	Leaf (2)	n.a. (2)	124:130	132:135	195:195	143:143	208:208	241:253	216:222	Admixed
MLG710	1	HRV008	HRV (1)	Leaf (1)	n.a. (1)	124:130	132:135	195:195	143:147	208:208	245:251	210:222	Admixed
MLG711	1	HRV010	HRV (1)	Leaf (1)	Vvin (1)	121:130	129:129	195:195	143:143	202:202	241:251	219:222	Eu2
MLG712	1	HRV011	HRV (1)	Leaf (1)	Vvin (1)	115:127	129:129	195:204	143:143	208:208	245:251	219:222	Eu2
MLG713	1	HRV012	HRV (1)	Leaf (1)	Vvin (1)	115:127	132:132	195:195	145:145	205:208	251:253	216:216	Admixed
MLG714	3	HRV013, HRV015, HRV016	HRV (3)	Leaf (3)	Rts (2), Vvin (1)	130:130	132:135	195:204	143:145	202:202	245:251	222:222	Admixed
MLG715	1	HRV014	HRV (1)	Leaf (1)	Rts (1)	130:130	132:135	195:204	143:143	202:202	245:245	222:222	Admixed
MLG716	2	HRV017, HRV018	HRV (2)	Leaf (2)	Rts (2)	124:127	132:132	195:204	145:145	202:208	245:249	210:222	Eu1
MLG717	4	HRV019, HRV020, HRV021, HRV022	HRV (4)	Leaf (4)	Rts (4)	115:130	132:132	195:195	143:145	202:202	251:251	216:216	Eu2
MLG718	1	HRV023	HRV (1)	Leaf (1)	Rts (1)	130:130	129:132	195:195	143:145	202:205	245:255	222:228	Admixed
MLG719	1	HRV026	HRV (1)	Leaf (1)	Rts (1)	124:127	132:132	195:204	145:147	202:208	251:253	219:219	Admixed
MLG720	1	HRV034	HRV (1)	Leaf (1)	Rts (1)	124:127	132:132	195:207	145:147	202:208	251:253	219:222	Admixed
MLG721	4	HRV037, HRV038,	HRV (4)	Leaf (4)	Rts (4)	115:124	129:132	195:204	143:143	202:202	251:255	222:222	Eu2

MLG	N	Sample(s) Code[1]	Country[2]	Feeding form	Host plant[3]	PhyIII_55	PhyIII_30	PhyIII_36	DV8	Dvit6	DVSSR4	DV4	Genetic group (K=2)
		HRV039, HRV040											
MLG722	2	HRV041, HRV042	HRV (2)	Leaf (2)	Rts (2)	124:127	132:135	204:204	145:147	208:208	251:251	222:222	Admixed
MLG723	1	HRV043	HRV (1)	Leaf (1)	Rts (1)	115:127	129:132	195:204	145:147	202:208	251:251	222:222	Eu2
MLG724	1	HRV045	HRV (1)	Leaf (1)	Rts (1)	124:127	132:135	204:204	145:147	208:208	255:255	222:222	Eu2
MLG725	1	HRV046	HRV (1)	Leaf (1)	Rts (1)	124:127	132:135	204:204	145:147	208:208	245:251	222:222	Admixed
MLG726	4	HRV047, HRV048, HRV049, HRV050	HRV (4)	Leaf (4)	Rts (4)	127:130	132:132	195:195	145:145	205:205	251:253	222:222	Eu1
MLG727	1	HRV051	HRV (1)	Leaf (1)	Rts (1)	127:130	132:132	195:195	145:145	205:205	251:251	222:222	Admixed
MLG728	1	HRV052	HRV (1)	Leaf (1)	Vvin (1)	124:124	132:141	204:204	147:147	196:196	243:243	222:222	Eu1
MLG729	1	HRV053	HRV (1)	Leaf (1)	Rts (1)	121:127	132:132	195:207	143:143	205:208	241:251	216:222	Eu1
MLG730	2	HRV054, HRV095	HRV (2)	Leaf (2)	Rts (1), Vvin (1)	127:130	129:132	195:195	143:145	202:208	245:251	216:219	Admixed
MLG731	4	HRV055, HRV056, HRV057, HRV060	HRV (4)	Leaf (4)	Rts (4)	130:130	129:132	195:204	143:143	202:208	249:249	216:216	Admixed
MLG732	2	HRV058, HRV059	HRV (2)	Leaf (2)	Rts (2)	130:133	129:132	195:204	143:143	202:208	251:251	219:219	Eu2
MLG733	1	HRV061	HRV (1)	Leaf (1)	Rts (1)	127:130	132:132	195:195	145:147	202:208	251:251	213:213	Admixed
MLG734	1	HRV062	HRV (1)	Leaf (1)	Rts (1)	124:127	132:132	195:198	145:145	196:205	249:249	216:219	Eu1
MLG735	1	HRV063	HRV (1)	Leaf (1)	Rts (1)	121:127	132:132	198:204	143:145	205:205	251:253	219:219	Admixed
MLG736	1	HRV064	HRV (1)	Leaf (1)	Rts (1)	121:124	132:132	195:195	143:145	202:202	241:251	219:219	Admixed
MLG737	1	HRV065	HRV (1)	Leaf (1)	Rts (1)	124:127	132:132	195:195	145:145	205:205	249:249	222:222	Eu1
MLG738	1	HRV066	HRV (1)	Leaf (1)	Rts (1)	130:130	132:132	195:204	145:145	205:208	251:251	219:219	Admixed
MLG739	1	HRV067	HRV (1)	Leaf (1)	Rts (1)	130:130	132:132	195:204	145:145	205:208	241:249	210:219	Eu1
MLG740	1	HRV068	HRV (1)	Leaf (1)	Rts (1)	130:130	132:132	195:195	143:145	205:205	249:251	210:210	Eu1

MLG	N	Sample(s) Code[1]	Country[2]	Feeding form	Host plant[3]	PhyIII_55	PhyIII_30	PhyIII_36	DV8	Dvit6	DVSSR4	DV4	Genetic group (K=2)
MLG741	1	HRV069	HRV (1)	Leaf (1)	Rts (1)	121:127	132:132	198:204	143:145	205:205	251:251	222:222	Admixed
MLG742	1	HRV070	HRV (1)	Leaf (1)	Rts (1)	130:130	129:132	195:204	145:145	205:208	239:251	213:213	Admixed
MLG743	3	HRV071, HRV072, HRV073	HRV (3)	Leaf (3)	Rts (3)	127:127	132:132	195:204	143:145	202:208	241:251	219:219	Admixed
MLG744	1	HRV074	HRV (1)	Leaf (1)	Rts (1)	127:127	132:132	195:204	143:145	202:208	251:251	219:219	Admixed
MLG745	1	HRV075	HRV (1)	Leaf (1)	Rts (1)	127:127	132:132	195:204	145:145	202:208	251:251	210:210	Eu1
MLG746	1	HRV076	HRV (1)	Leaf (1)	Rts (1)	127:127	132:132	195:204	143:145	202:208	239:249	216:216	Admixed
MLG747	1	HRV077	HRV (1)	Leaf (1)	Rts (1)	127:127	132:132	195:204	143:145	202:202	239:249	216:216	Admixed
MLG748	1	HRV078	HRV (1)	Leaf (1)	Vvin (1)	124:124	129:132	195:195	143:145	202:202	251:253	216:225	Admixed
MLG749	1	HRV079	HRV (1)	Leaf (1)	Vvin (1)	124:127	132:132	195:195	143:145	202:202	253:253	216:225	Admixed
MLG750	1	HRV080	HRV (1)	Leaf (1)	Vvin (1)	124:124	129:135	195:195	143:145	202:202	251:251	216:222	Eu2
MLG751	1	HRV081	HRV (1)	Leaf (1)	Vvin (1)	124:124	129:132	195:195	143:143	202:202	253:253	216:225	Admixed
MLG752	1	HRV082	HRV (1)	Leaf (1)	Vvin (1)	124:127	123:129	195:195	143:145	202:202	253:253	216:225	Admixed
MLG753	2	HRV083, HRV084	HRV (2)	Leaf (2)	Vvin (2)	124:127	123:129	195:195	143:143	202:208	253:253	216:216	Admixed
MLG754	1	HRV085	HRV (1)	Leaf (1)	Vvin (1)	124:130	132:132	195:195	143:143	202:202	253:253	216:225	Admixed
MLG755	2	HRV086, HRV087	HRV (2)	Leaf (2)	Vvin (2)	124:127	123:129	195:195	143:145	202:208	253:253	216:216	Admixed
MLG756	1	HRV088	HRV (1)	Leaf (1)	Vvin (1)	130:130	123:129	195:195	143:143	202:208	253:253	216:216	Eu2
MLG757	1	HRV089	HRV (1)	Leaf (1)	Vvin (1)	124:130	123:129	195:195	143:143	202:208	253:253	216:216	Admixed
MLG758	1	HRV090	HRV (1)	Leaf (1)	Vvin (1)	121:130	129:132	195:195	145:147	204:207	241:251	222:222	Admixed
MLG759	1	HRV091	HRV (1)	Leaf (1)	Vvin (1)	124:127	129:129	195:198	143:143	202:208	251:251	219:219	Eu2
MLG760	1	HRV092	HRV (1)	Leaf (1)	Vvin (1)	121:130	132:132	195:207	143:143	204:207	241:251	216:222	Admixed
MLG761	1	HRV093	HRV (1)	Leaf (1)	Vvin (1)	130:130	129:132	195:195	143:145	202:202	253:253	216:225	Admixed
MLG762	1	HRV094	HRV (1)	Leaf (1)	Vvin (1)	130:130	129:129	195:195	145:147	204:207	241:251	222:222	Admixed
MLG763	1	HRV096	HRV (1)	Leaf (1)	Vvin (1)	124:127	129:129	195:198	143:143	202:208	251:251	210:219	Admixed
MLG764	1	HRV097	HRV (1)	Leaf (1)	Vvin (1)	127:127	129:135	192:195	143:143	205:208	251:251	210:210	Admixed

MLG	N	Sample(s) Code[1]	Country[2]	Feeding form	Host plant[3]	PhyIII_55	PhyIII_30	PhyIII_36	DV8	Dvit6	DVSSR4	DV4	Genetic group (K=2)
MLG765	3	HRV098, HRV099, HRV100	HRV (3)	Leaf (3)	Vvin (3)	124:124	129:129	195:195	143:145	205:208	251:251	219:225	Admixed
MLG766	2	HRV101, HRV102	HRV (2)	Leaf (2)	Vvin (2)	127:127	129:129	195:195	143:145	205:208	251:251	219:219	Admixed
MLG767	1	HRV103	HRV (1)	Leaf (1)	Vvin (1)	127:127	129:129	195:195	143:143	202:208	251:251	219:219	Eu2
MLG768	1	HRV104	HRV (1)	Leaf (1)	Vvin (1)	124:124	123:123	195:195	143:145	205:208	251:251	219:225	Admixed
MLG769	1	HRV105	HRV (1)	Leaf (1)	Vvin (1)	124:139	129:129	195:195	143:145	202:208	251:251	222:222	Eu2
MLG770	1	HRV106	HRV (1)	Leaf (1)	Vvin (1)	127:133	129:135	195:198	145:145	202:208	251:253	219:222	Eu2
MLG771	1	HRV107	HRV (1)	Leaf (1)	Vvin (1)	127:133	129:135	195:195	143:145	202:208	251:251	216:222	Eu2
MLG772	1	ROU001	ROU (1)	Leaf (1)	Rts (1)	127:130	132:132	195:195	143:143	208:208	251:251	216:219	Admixed
MLG773	1	ROU002	ROU (1)	Leaf (1)	Rts (1)	124:124	132:141	195:195	143:145	202:208	241:251	222:222	Admixed
MLG774	1	ROU003	ROU (1)	Leaf (1)	Rts (1)	124:127	132:135	195:195	145:145	208:208	251:251	219:222	Admixed

[1]: See Supplementary File 3 for Sample(s) codes details
[2]: AUT: Austria; CHE: Switzerland; DEU: Germany; HUN: Hungary; HRV: Croatia; ITA: Italy; ROU: Romania; SRB: Serbia; URY: Uruguay; ZAF: South Africa
[3]: Vvin: *Vitis vinifera* L. cultivar; Rts: *Vitis* spp. interspecific rootstock; HDP: *Vitis* spp. interspecific hybrid direct-producer; RGV: *Vitis* spp. interspecific resistant grape varieties
n.a.: not available

Supplementary Table S2. Genetic group assignation at K=2, 3 and 5 for 302 grape phylloxera MLGs identified in Europe (143 of Eu1 and 159 of Eu2) and 319 MLGs from the native range.

Sample name	Origin	Source	Genetic group at K=2	Genetic group at K=3	Genetic group at K=5
AR0681071	Native range (AR)	Lund et al. (2017)	Eu	Eu1'	Eu1'
AR0681072	Native range (AR)	Lund et al. (2017)	Eu	Eu1'	Eu1'
AR0681081	Native range (AR)	Lund et al. (2017)	Eu	Eu1'	Eu1'
AR0691092	Native range (AR)	Lund et al. (2017)	NR	NR	NR1
AR0691093	Native range (AR)	Lund et al. (2017)	NR	NR	NR1
AR0691095	Native range (AR)	Lund et al. (2017)	NR	NR	NR1
AR0691101	Native range (AR)	Lund et al. (2017)	NR	NR	NR1
AR0691102	Native range (AR)	Lund et al. (2017)	NR	NR	NR1
AR0691103	Native range (AR)	Lund et al. (2017)	NR	NR	NR1
AR0691104	Native range (AR)	Lund et al. (2017)	NR	NR	NR1
AR0701113	Native range (AR)	Lund et al. (2017)	NR	NR	NR1
AR0701121	Native range (AR)	Lund et al. (2017)	NR	admixed	NR1
AR0701131	Native range (AR)	Lund et al. (2017)	NR	NR	NR1
AR0701132	Native range (AR)	Lund et al. (2017)	NR	NR	NR1
AR0711141	Native range (AR)	Lund et al. (2017)	NR	NR	NR2
ARG1111871	Introduced region (ARG)	Lund et al. (2017)	Eu	Eu1'	admixed
ARG1111881	Introduced region (ARG)	Lund et al. (2017)	admixed	admixed	admixed
AUS1071701	Introduced region (AUT)	Lund et al. (2017)	Eu	Eu2'	Eu2'
AUS1071711	Introduced region (AUT)	Lund et al. (2017)	Eu	Eu2'	Eu2'
AUS1071721	Introduced region (AUT)	Lund et al. (2017)	Eu	Eu2'	Eu2'
AUS1071731	Introduced region (AUT)	Lund et al. (2017)	Eu	admixed	admixed
AUS1071741	Introduced region (AUT)	Lund et al. (2017)	Eu	Eu2'	Eu2'
AUS1071751	Introduced region (AUT)	Lund et al. (2017)	Eu	Eu2'	Eu2'
AUS1071761	Introduced region (AUT)	Lund et al. (2017)	Eu	admixed	admixed
AZ0861401	Native range (AZ)	Lund et al. (2017)	NR	NR	admixed

Sample name	Origin	Source	Genetic group at K=2	Genetic group at K=3	Genetic group at K=5
AZ0861402	Native range (AZ)	Lund et al. (2017)	admixed	Eu2'	NR3
AZ0861403	Native range (AZ)	Lund et al. (2017)	NR	NR	NR3
AZ0861404	Native range (AZ)	Lund et al. (2017)	NR	NR	NR3
AZ0861405	Native range (AZ)	Lund et al. (2017)	NR	NR	NR3
AZ0871412	Native range (AZ)	Lund et al. (2017)	admixed	admixed	NR3
AZ0871413	Native range (AZ)	Lund et al. (2017)	admixed	admixed	NR3
AZ0881421	Native range (AZ)	Lund et al. (2017)	admixed	admixed	NR3
AZ0881422	Native range (AZ)	Lund et al. (2017)	NR	NR	NR3
AZ0881423	Native range (AZ)	Lund et al. (2017)	NR	NR	NR3
AZ0881425	Native range (AZ)	Lund et al. (2017)	NR	NR	NR3
AZ0891432	Native range (AZ)	Lund et al. (2017)	NR	NR	NR3
AZ0901442	Native range (AZ)	Lund et al. (2017)	NR	NR	NR3
AZ0901444	Native range (AZ)	Lund et al. (2017)	Eu	Eu2'	NR3
AZ0901445	Native range (AZ)	Lund et al. (2017)	NR	admixed	NR3
AZ0911451	Native range (AZ)	Lund et al. (2017)	Eu	admixed	admixed
AZ0911454	Native range (AZ)	Lund et al. (2017)	NR	NR	NR3
AZ0911455	Native range (AZ)	Lund et al. (2017)	NR	NR	NR3
AZ0921461	Native range (AZ)	Lund et al. (2017)	NR	NR	NR3
AZ0931474	Native range (AZ)	Lund et al. (2017)	NR	NR	NR3
AZ0931482	Native range (AZ)	Lund et al. (2017)	admixed	admixed	NR3
AZ0941501	Native range (AZ)	Lund et al. (2017)	admixed	admixed	NR3
AZ0941504	Native range (AZ)	Lund et al. (2017)	NR	NR	NR3
BRA1091811	Introduced region (BRA)	Lund et al. (2017)	Eu	Eu1'	Eu1'
BRA1091821	Introduced region (BRA)	Lund et al. (2017)	Eu	admixed	admixed
BRA1091831	Introduced region (BRA)	Lund et al. (2017)	Eu	Eu1'	Eu1'
CA0010011	Introduced range (CA)	Lund et al. (2017)	NR	admixed	admixed
CA0020021	Introduced range (CA)	Lund et al. (2017)	Eu	admixed	admixed
CA0030032	Introduced range (CA)	Lund et al. (2017)	Eu	admixed	admixed

Sample name	Origin	Source	Genetic group at K=2	Genetic group at K=3	Genetic group at K=5
CA0040041	Introduced range (CA)	Lund et al. (2017)	Eu	admixed	admixed
CA0050051	Introduced range (CA)	Lund et al. (2017)	Eu	admixed	admixed
CA0060061	Introduced range (CA)	Lund et al. (2017)	NR	NR	admixed
FL0560861	Native range (FL)	Lund et al. (2017)	NR	NR	NR1
FL0560862	Native range (FL)	Lund et al. (2017)	NR	NR	NR1
FL0560863	Native range (FL)	Lund et al. (2017)	NR	NR	NR1
FL0560864	Native range (FL)	Lund et al. (2017)	NR	NR	admixed
FL0560865	Native range (FL)	Lund et al. (2017)	NR	NR	admixed
FL0570872	Native range (FL)	Lund et al. (2017)	NR	NR	admixed
FL0570877	Native range (FL)	Lund et al. (2017)	NR	NR	admixed
HUN1061631	Introduced region (HUN)	Lund et al. (2017)	Eu	admixed	admixed
HUN1061641	Introduced region (HUN)	Lund et al. (2017)	Eu	Eu2'	Eu2'
HUN1061661	Introduced region (HUN)	Lund et al. (2017)	Eu	Eu1'	admixed
HUN1061671	Introduced region (HUN)	Lund et al. (2017)	admixed	admixed	admixed
HUN1061681	Introduced region (HUN)	Lund et al. (2017)	Eu	admixed	admixed
HUN1061691	Introduced region (HUN)	Lund et al. (2017)	Eu	admixed	admixed
IN0290371	Native range (IN)	Lund et al. (2017)	admixed	Eu1'	admixed
IN0290372	Native range (IN)	Lund et al. (2017)	NR	admixed	admixed
IN0290373	Native range (IN)	Lund et al. (2017)	Eu	Eu1'	Eu1'
IN0300382	Native range (IN)	Lund et al. (2017)	NR	NR	NR2
IN0300383	Native range (IN)	Lund et al. (2017)	admixed	admixed	admixed
IN0310392	Native range (IN)	Lund et al. (2017)	NR	NR	NR2
IN0310393	Native range (IN)	Lund et al. (2017)	Eu	admixed	admixed
IN0320403	Native range (IN)	Lund et al. (2017)	Eu	Eu1'	Eu1'
IN0330412	Native range (IN)	Lund et al. (2017)	Eu	Eu1'	Eu1'
IN0330413	Native range (IN)	Lund et al. (2017)	Eu	Eu1'	Eu1'
MA0070071	Native range (MA)	Lund et al. (2017)	admixed	admixed	admixed
MA0080091	Native range (MA)	Lund et al. (2017)	Eu	Eu1'	Eu1'

Sample name	Origin	Source	Genetic group at K=2	Genetic group at K=3	Genetic group at K=5
MA0080092	Native range (MA)	Lund et al. (2017)	Eu	Eu1'	Eu1'
MA0080093	Native range (MA)	Lund et al. (2017)	Eu	Eu1'	Eu1'
MA0080094	Native range (MA)	Lund et al. (2017)	Eu	Eu1'	Eu1'
MA0090101	Native range (MA)	Lund et al. (2017)	Eu	Eu1'	Eu1'
MA0090102	Native range (MA)	Lund et al. (2017)	Eu	Eu1'	Eu1'
MA0090105	Native range (MA)	Lund et al. (2017)	admixed	Eu1'	admixed
MLG1	Eu2 (AUT)	This work	Eu	Eu2'	Eu2'
MLG101	Eu2 (AUT)	This work	Eu	Eu2'	Eu2'
MLG108	Eu2 (AUT)	This work	Eu	Eu2'	Eu2'
MLG114	Eu2 (AUT)	This work	Eu	Eu2'	Eu2'
MLG119	Eu2 (AUT)	This work	Eu	Eu2'	Eu2'
MLG122	Eu2 (AUT)	This work	Eu	Eu2'	Eu2'
MLG124	Eu2 (AUT)	This work	Eu	Eu2'	Eu2'
MLG125	Eu2 (AUT)	This work	Eu	Eu2'	Eu2'
MLG126	Eu2 (AUT)	This work	Eu	Eu2'	Eu2'
MLG127	Eu2 (AUT)	This work	Eu	Eu2'	Eu2'
MLG13	Eu2 (AUT)	This work	Eu	Eu2'	Eu2'
MLG130	Eu2 (AUT)	This work	Eu	Eu2'	Eu2'
MLG133	Eu2 (AUT)	This work	Eu	Eu2'	Eu2'
MLG134	Eu2 (AUT)	This work	Eu	Eu2'	Eu2'
MLG136	Eu2 (AUT)	This work	Eu	Eu2'	Eu2'
MLG138	Eu2 (AUT)	This work	Eu	Eu2'	Eu2'
MLG139	Eu2 (AUT)	This work	Eu	Eu2'	Eu2'
MLG141	Eu2 (AUT)	This work	Eu	Eu2'	Eu2'
MLG142	Eu2 (AUT)	This work	Eu	Eu2'	Eu2'
MLG144	Eu2 (AUT)	This work	Eu	Eu2'	Eu2'
MLG145	Eu2 (AUT)	This work	Eu	Eu2'	Eu2'
MLG146	Eu2 (AUT)	This work	Eu	Eu2'	Eu2'

Sample name	Origin	Source	Genetic group at K=2	Genetic group at K=3	Genetic group at K=5
MLG147	Eu2 (AUT)	This work	Eu	Eu2'	Eu2'
MLG149	Eu2 (AUT)	This work	Eu	Eu2'	Eu2'
MLG15	Eu2 (AUT)	This work	Eu	Eu2'	Eu2'
MLG150	Eu2 (AUT)	This work	Eu	Eu2'	Eu2'
MLG154	Eu1 (AUT)	This work	Eu	Eu1'	Eu1'
MLG158	Eu2 (AUT)	This work	Eu	Eu2'	Eu2'
MLG16	Eu2 (AUT)	This work	Eu	Eu2'	Eu2'
MLG160	Eu2 (AUT)	This work	Eu	Eu2'	Eu2'
MLG161	Eu2 (AUT)	This work	Eu	Eu2'	Eu2'
MLG163	Eu2 (AUT)	This work	Eu	Eu2'	Eu2'
MLG166	Eu2 (AUT)	This work	Eu	Eu2'	Eu2'
MLG17	Eu2 (AUT)	This work	Eu	admixed	admixed
MLG170	Eu2 (AUT)	This work	Eu	Eu2'	Eu2'
MLG171	Eu2 (AUT)	This work	Eu	Eu2'	Eu2'
MLG172	Eu2 (AUT)	This work	Eu	Eu2'	Eu2'
MLG173	Eu2 (AUT)	This work	Eu	Eu2'	Eu2'
MLG174	Eu2 (AUT)	This work	Eu	Eu2'	Eu2'
MLG175	Eu2 (AUT)	This work	Eu	Eu2'	Eu2'
MLG176	Eu2 (AUT)	This work	Eu	Eu2'	Eu2'
MLG177	Eu2 (AUT)	This work	Eu	Eu2'	Eu2'
MLG178	Eu2 (AUT)	This work	Eu	Eu2'	Eu2'
MLG179	Eu2 (AUT)	This work	Eu	Eu2'	Eu2'
MLG18	Eu2 (AUT)	This work	Eu	Eu2'	Eu2'
MLG180	Eu2 (AUT)	This work	Eu	Eu2'	Eu2'
MLG183	Eu1 (AUT)	This work	Eu	admixed	admixed
MLG185	Eu1 (AUT)	This work	Eu	admixed	admixed
MLG187	Eu2 (AUT)	This work	Eu	Eu2'	Eu2'
MLG188	Eu2 (AUT)	This work	Eu	Eu2'	Eu2'

Sample name	Origin	Source	Genetic group at K=2	Genetic group at K=3	Genetic group at K=5
MLG189	Eu2 (AUT)	This work	Eu	Eu2'	Eu2'
MLG19	Eu2 (AUT)	This work	Eu	Eu2'	Eu2'
MLG190	Eu2 (AUT)	This work	Eu	Eu2'	Eu2'
MLG191	Eu2 (AUT)	This work	Eu	Eu2'	Eu2'
MLG192	Eu2 (AUT)	This work	Eu	Eu2'	Eu2'
MLG197	Eu2 (AUT)	This work	Eu	Eu2'	Eu2'
MLG199	Eu2 (AUT)	This work	Eu	Eu2'	Eu2'
MLG2	Eu2 (AUT)	This work	Eu	Eu2'	Eu2'
MLG202	Eu2 (AUT)	This work	Eu	Eu2'	Eu2'
MLG208	Eu2 (AUT)	This work	Eu	Eu2'	Eu2'
MLG209	Eu2 (AUT)	This work	Eu	Eu2'	Eu2'
MLG210	Eu2 (AUT)	This work	Eu	Eu2'	Eu2'
MLG214	Eu2 (AUT)	This work	Eu	Eu2'	Eu2'
MLG217	Eu2 (AUT)	This work	admixed	admixed	admixed
MLG222	Eu2 (AUT)	This work	Eu	Eu2'	Eu2'
MLG224	Eu2 (AUT)	This work	Eu	Eu2'	Eu2'
MLG226	Eu2 (AUT)	This work	Eu	Eu2'	Eu2'
MLG227	Eu1 (AUT)	This work	Eu	Eu1'	Eu1'
MLG228	Eu1 (AUT)	This work	Eu	Eu1'	Eu1'
MLG229	Eu2 (AUT)	This work	Eu	Eu2'	Eu2'
MLG234	Eu1 (DEU)	This work	Eu	admixed	admixed
MLG239	Eu2 (DEU)	This work	Eu	Eu2'	Eu2'
MLG24	Eu2 (AUT)	This work	Eu	Eu2'	Eu2'
MLG243	Eu1 (DEU)	This work	Eu	Eu1'	Eu1'
MLG244	Eu1 (DEU)	This work	Eu	Eu1'	Eu1'
MLG246	Eu1 (DEU)	This work	Eu	Eu1'	Eu1'
MLG248	Eu2 (DEU)	This work	Eu	Eu2'	Eu2'
MLG251	Eu2 (DEU)	This work	Eu	Eu2'	Eu2'

Sample name	Origin	Source	Genetic group at K=2	Genetic group at K=3	Genetic group at K=5
MLG253	Eu2 (DEU)	This work	Eu	Eu2'	Eu2'
MLG257	Eu1 (DEU)	This work	Eu	Eu1'	Eu1'
MLG269	Eu1 (DEU)	This work	NR	NR	NR1
MLG27	Eu2 (AUT)	This work	admixed	admixed	admixed
MLG271	Eu1 (DEU)	This work	Eu	Eu1'	Eu1'
MLG272	Eu1 (DEU)	This work	Eu	Eu1'	Eu1'
MLG274	Eu1 (DEU)	This work	Eu	Eu1'	Eu1'
MLG275	Eu1 (DEU)	This work	Eu	Eu1'	Eu1'
MLG276	Eu1 (DEU)	This work	Eu	Eu1'	Eu1'
MLG277	Eu1 (DEU)	This work	Eu	Eu1'	Eu1'
MLG278	Eu1 (DEU)	This work	Eu	Eu1'	Eu1'
MLG281	Eu1 (DEU)	This work	Eu	Eu1'	Eu1'
MLG283	Eu1 (DEU)	This work	Eu	Eu1'	Eu1'
MLG284	Eu1 (DEU)	This work	Eu	Eu1'	Eu1'
MLG285	Eu1 (DEU)	This work	Eu	Eu1'	Eu1'
MLG289	Eu1 (DEU)	This work	Eu	Eu1'	Eu1'
MLG291	Eu2 (DEU)	This work	Eu	Eu2'	Eu2'
MLG292	Eu2 (DEU)	This work	Eu	admixed	admixed
MLG296	Eu2 (DEU)	This work	Eu	admixed	admixed
MLG297	Eu1 (DEU)	This work	Eu	Eu1'	Eu1'
MLG298	Eu1 (DEU)	This work	Eu	Eu1'	Eu1'
MLG299	Eu1 (DEU)	This work	Eu	Eu1'	Eu1'
MLG307	Eu2 (DEU)	This work	Eu	Eu2'	Eu2'
MLG31	Eu2 (AUT)	This work	Eu	Eu2'	Eu2'
MLG310	Eu1 (CHE)	This work	Eu	Eu1'	Eu1'
MLG316	Eu1 (CHE)	This work	Eu	Eu1'	Eu1'
MLG323	Eu1 (CHE)	This work	Eu	Eu1'	Eu1'
MLG324	Eu1 (CHE)	This work	Eu	Eu1'	Eu1'

Sample name	Origin	Source	Genetic group at K=2	Genetic group at K=3	Genetic group at K=5
MLG327	Eu1 (CHE)	This work	Eu	admixed	admixed
MLG328	Eu1 (CHE)	This work	Eu	Eu1'	Eu1'
MLG33	Eu2 (AUT)	This work	Eu	Eu2'	Eu2'
MLG333	Eu1 (CHE)	This work	Eu	Eu1'	Eu1'
MLG334	Eu1 (CHE)	This work	Eu	admixed	Eu1'
MLG335	Eu1 (CHE)	This work	Eu	Eu1'	Eu1'
MLG337	Eu1 (CHE)	This work	Eu	Eu1'	Eu1'
MLG340	Eu1 (CHE)	This work	Eu	Eu1'	Eu1'
MLG341	Eu1 (CHE)	This work	Eu	Eu1'	Eu1'
MLG35	Eu2 (AUT)	This work	Eu	Eu2'	Eu2'
MLG354	Eu1 (CHE)	This work	Eu	Eu1'	Eu1'
MLG355	Eu1 (CHE)	This work	Eu	Eu1'	Eu1'
MLG358	Eu1 (CHE)	This work	Eu	Eu1'	Eu1'
MLG36	Eu2 (AUT)	This work	Eu	Eu2'	Eu2'
MLG361	Eu1 (CHE)	This work	Eu	Eu1'	Eu1'
MLG362	Eu1 (CHE)	This work	Eu	Eu1'	Eu1'
MLG364	Eu1 (CHE)	This work	Eu	admixed	admixed
MLG371	Eu1 (CHE)	This work	Eu	Eu1'	Eu1'
MLG372	Eu1 (CHE)	This work	Eu	Eu1'	Eu1'
MLG373	Eu1 (CHE)	This work	Eu	Eu1'	Eu1'
MLG374	Eu1 (CHE)	This work	Eu	Eu1'	Eu1'
MLG375	Eu1 (CHE)	This work	Eu	Eu1'	Eu1'
MLG376	Eu1 (CHE)	This work	Eu	Eu1'	Eu1'
MLG38	Eu2 (AUT)	This work	Eu	Eu2'	Eu2'
MLG39	Eu2 (AUT)	This work	Eu	admixed	admixed
MLG397	Eu1 (CHE)	This work	Eu	admixed	admixed
MLG398	Eu1 (CHE)	This work	Eu	Eu1'	Eu1'
MLG403	Eu1 (CHE)	This work	Eu	Eu1'	Eu1'

Sample name	Origin	Source	Genetic group at K=2	Genetic group at K=3	Genetic group at K=5
MLG405	Eu1 (CHE)	This work	Eu	Eu1'	Eu1'
MLG41	Eu2 (AUT)	This work	Eu	admixed	admixed
MLG410	Eu1 (CHE)	This work	Eu	admixed	admixed
MLG411	Eu1 (CHE)	This work	Eu	admixed	admixed
MLG412	Eu1 (CHE)	This work	Eu	Eu1'	Eu1'
MLG413	Eu1 (CHE)	This work	Eu	Eu1'	Eu1'
MLG414	Eu1 (CHE)	This work	Eu	Eu1'	Eu1'
MLG415	Eu1 (CHE)	This work	Eu	Eu1'	Eu1'
MLG416	Eu1 (CHE)	This work	Eu	Eu1'	Eu1'
MLG417	Eu1 (CHE)	This work	Eu	Eu1'	Eu1'
MLG419	Eu1 (CHE)	This work	Eu	Eu1'	Eu1'
MLG420	Eu1 (CHE)	This work	Eu	Eu1'	Eu1'
MLG422	Eu1 (CHE)	This work	Eu	Eu1'	Eu1'
MLG423	Eu1 (CHE)	This work	Eu	Eu1'	Eu1'
MLG426	Eu1 (CHE)	This work	Eu	Eu1'	Eu1'
MLG427	Eu1 (CHE)	This work	Eu	Eu1'	Eu1'
MLG430	Eu1 (CHE)	This work	Eu	Eu1'	Eu1'
MLG436	Eu1 (CHE)	This work	Eu	Eu1'	Eu1'
MLG437	Eu1 (CHE)	This work	Eu	Eu1'	Eu1'
MLG438	Eu1 (CHE)	This work	Eu	Eu1'	Eu1'
MLG440	Eu1 (CHE)	This work	Eu	Eu1'	Eu1'
MLG441	Eu1 (CHE)	This work	Eu	Eu1'	Eu1'
MLG442	Eu1 (CHE)	This work	Eu	Eu1'	Eu1'
MLG443	Eu1 (CHE)	This work	Eu	Eu1'	Eu1'
MLG444	Eu1 (CHE)	This work	Eu	Eu1'	Eu1'
MLG445	Eu1 (CHE)	This work	Eu	Eu1'	Eu1'
MLG446	Eu1 (CHE)	This work	Eu	Eu1'	Eu1'
MLG448	Eu1 (CHE)	This work	Eu	Eu1'	Eu1'

Sample name	Origin	Source	Genetic group at K=2	Genetic group at K=3	Genetic group at K=5
MLG449	Eu1 (CHE)	This work	Eu	Eu1'	Eu1'
MLG45	Eu2 (AUT)	This work	Eu	Eu2'	Eu2'
MLG451	Eu1 (CHE)	This work	Eu	Eu1'	Eu1'
MLG452	Eu1 (CHE)	This work	Eu	Eu1'	Eu1'
MLG453	Eu1 (CHE)	This work	Eu	Eu1'	Eu1'
MLG461	Eu2 (CHE)	This work	Eu	Eu2'	Eu2'
MLG466	Eu1 (CHE)	This work	Eu	Eu1'	Eu1'
MLG470	Eu2 (CHE)	This work	Eu	Eu2'	Eu2'
MLG472	Eu2 (CHE)	This work	Eu	Eu2'	Eu2'
MLG473	Eu2 (CHE)	This work	Eu	Eu2'	Eu2'
MLG477	Eu2 (CHE)	This work	Eu	Eu2'	Eu2'
MLG478	Eu2 (CHE)	This work	Eu	Eu2'	Eu2'
MLG480	Eu1 (URY)	This work	Eu	Eu1'	Eu1'
MLG481	Eu1 (URY)	This work	Eu	Eu1'	Eu1'
MLG482	Eu1 (URY)	This work	Eu	Eu1'	Eu1'
MLG483	Eu1 (URY)	This work	Eu	Eu1'	Eu1'
MLG484	Eu1 (URY)	This work	Eu	Eu1'	Eu1'
MLG485	Eu1 (URY)	This work	Eu	Eu1'	Eu1'
MLG486	Eu1 (URY)	This work	Eu	Eu1'	Eu1'
MLG487	Eu1 (URY)	This work	Eu	Eu1'	Eu1'
MLG488	Eu1 (URY)	This work	Eu	Eu1'	Eu1'
MLG490	Eu1 (URY)	This work	Eu	Eu1'	Eu1'
MLG493	Eu1 (URY)	This work	Eu	Eu1'	Eu1'
MLG494	Eu1 (URY)	This work	Eu	Eu1'	Eu1'
MLG495	Eu1 (URY)	This work	Eu	Eu1'	Eu1'
MLG496	Eu1 (URY)	This work	Eu	Eu1'	Eu1'
MLG497	Eu1 (URY)	This work	Eu	Eu1'	Eu1'
MLG498	Eu1 (URY)	This work	Eu	Eu1'	Eu1'

Sample name	Origin	Source	Genetic group at K=2	Genetic group at K=3	Genetic group at K=5
MLG499	Eu1 (URY)	This work	Eu	Eu1'	Eu1'
MLG500	Eu1 (URY)	This work	Eu	Eu1'	Eu1'
MLG505	Eu1 (URY)	This work	Eu	Eu1'	Eu1'
MLG516	Eu2 (SRB)	This work	Eu	Eu2'	Eu2'
MLG521	Eu2 (SRB)	This work	Eu	Eu2'	Eu2'
MLG527	Eu2 (SRB)	This work	Eu	Eu2'	Eu2'
MLG528	Eu2 (SRB)	This work	Eu	Eu2'	Eu2'
MLG529	Eu2 (SRB)	This work	Eu	Eu2'	Eu2'
MLG531	Eu2 (SRB)	This work	Eu	Eu2'	Eu2'
MLG533	Eu2 (SRB)	This work	Eu	Eu2'	Eu2'
MLG536	Eu2 (SRB)	This work	Eu	Eu2'	Eu2'
MLG537	Eu2 (SRB)	This work	Eu	Eu2'	Eu2'
MLG542	Eu1 (ITA)	This work	Eu	admixed	admixed
MLG543	Eu1 (ITA)	This work	Eu	admixed	admixed
MLG545	Eu1 (ITA)	This work	Eu	Eu1'	Eu1'
MLG558	Eu1 (ITA)	This work	Eu	admixed	admixed
MLG56	Eu2 (AUT)	This work	Eu	Eu2'	Eu2'
MLG560	Eu1 (ITA)	This work	Eu	Eu1'	Eu1'
MLG562	Eu1 (ITA)	This work	Eu	Eu1'	Eu1'
MLG563	Eu1 (ITA)	This work	Eu	Eu1'	Eu1'
MLG566	Eu2 (ITA)	This work	Eu	Eu2'	Eu2'
MLG577	Eu1 (ITA)	This work	Eu	Eu1'	Eu1'
MLG578	Eu2 (ITA)	This work	Eu	Eu2'	Eu2'
MLG58	Eu2 (AUT)	This work	Eu	Eu2'	Eu2'
MLG580	Eu1 (ITA)	This work	Eu	Eu1'	Eu1'
MLG588	Eu2 (ITA)	This work	Eu	Eu2'	Eu2'
MLG59	Eu2 (AUT)	This work	Eu	Eu2'	Eu2'
MLG590	Eu2 (ITA)	This work	Eu	Eu2'	Eu2'

Sample name	Origin	Source	Genetic group at K=2	Genetic group at K=3	Genetic group at K=5
MLG592	Eu1 (ITA)	This work	Eu	Eu1'	Eu1'
MLG593	Eu1 (ITA)	This work	Eu	Eu1'	Eu1'
MLG594	Eu2 (ITA)	This work	Eu	Eu2'	Eu2'
MLG595	Eu1 (ITA)	This work	Eu	Eu1'	Eu1'
MLG596	Eu1 (ITA)	This work	Eu	Eu1'	Eu1'
MLG6	Eu1 (AUT)	This work	Eu	Eu1'	Eu1'
MLG603	Eu2 (HUN)	This work	Eu	Eu2'	Eu2'
MLG606	Eu2 (HUN)	This work	Eu	Eu2'	Eu2'
MLG608	Eu2 (HUN)	This work	Eu	Eu2'	Eu2'
MLG61	Eu2 (AUT)	This work	Eu	Eu2'	Eu2'
MLG610	Eu2 (HUN)	This work	Eu	Eu2'	Eu2'
MLG611	Eu1 (HUN)	This work	admixed	admixed	admixed
MLG614	Eu1 (HUN)	This work	Eu	Eu1'	Eu1'
MLG623	Eu2 (HUN)	This work	Eu	Eu2'	Eu2'
MLG624	Eu1 (HUN)	This work	Eu	admixed	admixed
MLG626	Eu2 (HUN)	This work	Eu	Eu2'	Eu2'
MLG628	Eu1 (HUN)	This work	Eu	Eu1'	Eu1'
MLG629	Eu2 (HUN)	This work	Eu	Eu2'	Eu2'
MLG630	Eu2 (HUN)	This work	Eu	Eu2'	Eu2'
MLG632	Eu2 (HUN)	This work	Eu	Eu2'	Eu2'
MLG64	Eu2 (AUT)	This work	Eu	Eu2'	Eu2'
MLG642	Eu2 (HUN)	This work	Eu	Eu2'	Eu2'
MLG643	Eu2 (HUN)	This work	Eu	Eu2'	Eu2'
MLG647	Eu2 (HUN)	This work	Eu	Eu2'	Eu2'
MLG655	Eu1 (HUN)	This work	Eu	Eu2'	Eu2'
MLG669	Eu1 (HUN)	This work	admixed	admixed	admixed
MLG67	Eu1 (AUT)	This work	Eu	Eu1'	Eu1'
MLG676	Eu1 (HUN)	This work	Eu	Eu1'	Eu1'

Sample name	Origin	Source	Genetic group at K=2	Genetic group at K=3	Genetic group at K=5
MLG681	Eu1 (HUN)	This work	Eu	Eu1'	Eu1'
MLG683	Eu2 (HUN)	This work	Eu	Eu2'	Eu2'
MLG685	Eu1 (HUN)	This work	Eu	admixed	admixed
MLG686	Eu1 (HUN)	This work	Eu	Eu1'	Eu1'
MLG687	Eu1 (HUN)	This work	Eu	Eu1'	Eu1'
MLG688	Eu1 (HUN)	This work	Eu	Eu1'	Eu1'
MLG689	Eu2 (HUN)	This work	Eu	Eu2'	Eu2'
MLG690	Eu2 (HUN)	This work	Eu	Eu2'	Eu2'
MLG691	Eu2 (HUN)	This work	Eu	Eu2'	Eu2'
MLG692	Eu2 (HUN)	This work	Eu	Eu2'	Eu2'
MLG694	Eu2 (HUN)	This work	Eu	Eu2'	Eu2'
MLG697	Eu1 (HUN)	This work	Eu	Eu1'	Eu1'
MLG702	Eu1 (HUN)	This work	Eu	Eu1'	Eu1'
MLG704	Eu2 (HRV)	This work	Eu	Eu2'	Eu2'
MLG705	Eu2 (HRV)	This work	Eu	Eu2'	Eu2'
MLG706	Eu2 (HRV)	This work	Eu	Eu2'	Eu2'
MLG711	Eu2 (HRV)	This work	Eu	Eu2'	Eu2'
MLG712	Eu2 (HRV)	This work	Eu	Eu2'	Eu2'
MLG716	Eu1 (HRV)	This work	Eu	admixed	admixed
MLG717	Eu2 (HRV)	This work	Eu	Eu2'	Eu2'
MLG72	Eu2 (AUT)	This work	Eu	Eu2'	Eu2'
MLG721	Eu2 (HRV)	This work	Eu	Eu2'	Eu2'
MLG723	Eu2 (HRV)	This work	Eu	Eu2'	Eu2'
MLG724	Eu2 (HRV)	This work	Eu	Eu2'	admixed
MLG726	Eu1 (HRV)	This work	Eu	Eu1'	Eu1'
MLG728	Eu1 (HRV)	This work	Eu	Eu1'	Eu1'
MLG729	Eu1 (HRV)	This work	Eu	Eu1'	Eu1'
MLG73	Eu2 (AUT)	This work	Eu	Eu2'	Eu2'

Sample name	Origin	Source	Genetic group at K=2	Genetic group at K=3	Genetic group at K=5
MLG732	Eu2 (HRV)	This work	Eu	Eu2'	Eu2'
MLG734	Eu1 (HRV)	This work	Eu	Eu1'	Eu1'
MLG737	Eu1 (HRV)	This work	Eu	Eu1'	Eu1'
MLG739	Eu1 (HRV)	This work	Eu	admixed	admixed
MLG74	Eu2 (AUT)	This work	Eu	Eu2'	Eu2'
MLG740	Eu1 (HRV)	This work	Eu	Eu1'	Eu1'
MLG745	Eu1 (HRV)	This work	Eu	admixed	admixed
MLG75	Eu2 (AUT)	This work	Eu	Eu2'	Eu2'
MLG750	Eu2 (HRV)	This work	Eu	Eu2'	Eu2'
MLG756	Eu2 (HRV)	This work	Eu	Eu2'	Eu2'
MLG759	Eu2 (HRV)	This work	Eu	Eu2'	Eu2'
MLG76	Eu2 (AUT)	This work	Eu	Eu2'	Eu2'
MLG767	Eu2 (HRV)	This work	Eu	Eu2'	Eu2'
MLG769	Eu2 (HRV)	This work	Eu	Eu2'	Eu2'
MLG770	Eu2 (HRV)	This work	Eu	Eu2'	Eu2'
MLG771	Eu2 (HRV)	This work	Eu	Eu2'	Eu2'
MLG81	Eu2 (AUT)	This work	Eu	Eu2'	Eu2'
MLG82	Eu2 (AUT)	This work	Eu	Eu2'	Eu2'
MLG83	Eu2 (AUT)	This work	Eu	Eu2'	Eu2'
MLG85	Eu2 (AUT)	This work	Eu	Eu2'	Eu2'
MLG86	Eu2 (AUT)	This work	Eu	Eu2'	Eu2'
MLG88	Eu1 (AUT)	This work	Eu	Eu1'	Eu1'
MLG91	Eu2 (AUT)	This work	Eu	Eu2'	Eu2'
MLG93	Eu2 (AUT)	This work	Eu	Eu2'	Eu2'
MLG94	Eu2 (AUT)	This work	Eu	Eu2'	Eu2'
MLG96	Eu2 (AUT)	This work	Eu	Eu2'	Eu2'
MLG98	Eu2 (AUT)	This work	Eu	Eu2'	Eu2'
MLG99	Eu2 (AUT)	This work	admixed	admixed	admixed

Sample name	Origin	Source	Genetic group at K=2	Genetic group at K=3	Genetic group at K=5
MN0340421	Native range (MN)	Lund et al. (2017)	admixed	Eu1'	admixed
MN0340423	Native range (MN)	Lund et al. (2017)	NR	NR	NR1
MN0350431	Native range (MN)	Lund et al. (2017)	Eu	Eu1'	Eu1'
MN0350433	Native range (MN)	Lund et al. (2017)	admixed	Eu1'	admixed
MN0350442	Native range (MN)	Lund et al. (2017)	admixed	Eu1'	admixed
MN0350443	Native range (MN)	Lund et al. (2017)	admixed	admixed	admixed
MO0721152	Native range (MO)	Lund et al. (2017)	NR	NR	NR1
MO0721162	Native range (MO)	Lund et al. (2017)	NR	NR	NR1
MO0721163	Native range (MO)	Lund et al. (2017)	NR	NR	NR1
MO0721165	Native range (MO)	Lund et al. (2017)	NR	NR	NR1
MO0731171	Native range (MO)	Lund et al. (2017)	NR	NR	NR1
MO0741192	Native range (MO)	Lund et al. (2017)	NR	admixed	admixed
MO0741193	Native range (MO)	Lund et al. (2017)	NR	NR	NR2
MO0741194	Native range (MO)	Lund et al. (2017)	NR	NR	NR2
MO0741195	Native range (MO)	Lund et al. (2017)	NR	NR	NR2
MO0741196	Native range (MO)	Lund et al. (2017)	NR	NR	NR2
MO0741201	Native range (MO)	Lund et al. (2017)	NR	NR	NR2
MO0741202	Native range (MO)	Lund et al. (2017)	NR	NR	NR2
MO0751211	Native range (MO)	Lund et al. (2017)	NR	NR	NR2
MO0751221	Native range (MO)	Lund et al. (2017)	NR	NR	NR2
MO0761231	Native range (MO)	Lund et al. (2017)	NR	NR	admixed
MO0761232	Native range (MO)	Lund et al. (2017)	NR	NR	NR2
MO0771241	Native range (MO)	Lund et al. (2017)	NR	NR	NR2
MO0771242	Native range (MO)	Lund et al. (2017)	NR	NR	NR2
MO0771243	Native range (MO)	Lund et al. (2017)	NR	NR	NR2
MO0771251	Native range (MO)	Lund et al. (2017)	NR	NR	NR2
MO0771253	Native range (MO)	Lund et al. (2017)	NR	NR	NR2
MO0771254	Native range (MO)	Lund et al. (2017)	NR	NR	NR2

Sample name	Origin	Source	Genetic group at K=2	Genetic group at K=3	Genetic group at K=5
MO0781262	Native range (MO)	Lund et al. (2017)	NR	NR	NR2
MO0781271	Native range (MO)	Lund et al. (2017)	NR	NR	NR2
MO0781272	Native range (MO)	Lund et al. (2017)	NR	NR	NR2
MO0781281	Native range (MO)	Lund et al. (2017)	NR	NR	NR2
MO0801302	Native range (MO)	Lund et al. (2017)	NR	NR	NR2
MO0801303	Native range (MO)	Lund et al. (2017)	NR	NR	NR2
MO0801304	Native range (MO)	Lund et al. (2017)	NR	NR	NR2
MO0801312	Native range (MO)	Lund et al. (2017)	NR	NR	NR2
MO0801314	Native range (MO)	Lund et al. (2017)	NR	NR	NR2
MO0801321	Native range (MO)	Lund et al. (2017)	NR	admixed	admixed
MO0801322	Native range (MO)	Lund et al. (2017)	NR	NR	NR2
MO0801323	Native range (MO)	Lund et al. (2017)	NR	NR	NR2
NC0500751	Native range (NC)	Lund et al. (2017)	NR	NR	NR1
NC0500761	Native range (NC)	Lund et al. (2017)	NR	NR	NR1
NC0500763	Native range (NC)	Lund et al. (2017)	NR	NR	NR1
NC0510781	Native range (NC)	Lund et al. (2017)	NR	NR	NR1
NC0510782	Native range (NC)	Lund et al. (2017)	NR	NR	NR1
NC0510783	Native range (NC)	Lund et al. (2017)	NR	NR	NR1
NC0510784	Native range (NC)	Lund et al. (2017)	NR	NR	NR1
NC0510791	Native range (NC)	Lund et al. (2017)	NR	NR	NR1
NC0510793	Native range (NC)	Lund et al. (2017)	NR	NR	admixed
NC0530811	Native range (NC)	Lund et al. (2017)	NR	NR	NR1
NC0530813	Native range (NC)	Lund et al. (2017)	NR	NR	NR1
NC0540821	Native range (NC)	Lund et al. (2017)	NR	NR	admixed
NC0550824	Native range (NC)	Lund et al. (2017)	NR	NR	NR1
NC0550832	Native range (NC)	Lund et al. (2017)	NR	NR	NR1
NC0550841	Native range (NC)	Lund et al. (2017)	NR	NR	NR1
NC0550842	Native range (NC)	Lund et al. (2017)	NR	NR	NR1

Sample name	Origin	Source	Genetic group at K=2	Genetic group at K=3	Genetic group at K=5
NC0550843	Native range (NC)	Lund et al. (2017)	NR	NR	NR1
NM0951512	Native range (NM)	Lund et al. (2017)	NR	NR	admixed
NM0961522	Native range (NM)	Lund et al. (2017)	NR	NR	admixed
NM0961523	Native range (NM)	Lund et al. (2017)	NR	NR	admixed
NM0961524	Native range (NM)	Lund et al. (2017)	NR	NR	admixed
NM0971531	Native range (NM)	Lund et al. (2017)	NR	NR	admixed
NM0981542	Native range (NM)	Lund et al. (2017)	NR	admixed	NR3
NM0981543	Native range (NM)	Lund et al. (2017)	NR	NR	NR3
NM0991551	Native range (NM)	Lund et al. (2017)	NR	admixed	NR3
NM0991552	Native range (NM)	Lund et al. (2017)	NR	NR	admixed
NM0991553	Native range (NM)	Lund et al. (2017)	NR	NR	NR3
NM1001561	Native range (NM)	Lund et al. (2017)	NR	admixed	NR3
NM1001562	Native range (NM)	Lund et al. (2017)	NR	admixed	admixed
NM1001563	Native range (NM)	Lund et al. (2017)	NR	admixed	NR3
NM1011572	Native range (NM)	Lund et al. (2017)	NR	NR	NR3
NM1021583	Native range (NM)	Lund et al. (2017)	NR	NR	NR1
NM1041601	Native range (NM)	Lund et al. (2017)	NR	admixed	NR3
NM1041602	Native range (NM)	Lund et al. (2017)	NR	NR	NR1
NY0100111	Native range (NY)	Lund et al. (2017)	admixed	Eu1'	admixed
NY0110121	Native range (NY)	Lund et al. (2017)	Eu	Eu1'	Eu1'
NY0110122	Native range (NY)	Lund et al. (2017)	Eu	Eu1'	Eu1'
NY0110123	Native range (NY)	Lund et al. (2017)	Eu	Eu1'	Eu1'
NY0110131	Native range (NY)	Lund et al. (2017)	Eu	Eu1'	Eu1'
NY0120151	Native range (NY)	Lund et al. (2017)	Eu	Eu1'	Eu1'
NY0120161	Native range (NY)	Lund et al. (2017)	Eu	Eu1'	Eu1'
NY0140191	Native range (NY)	Lund et al. (2017)	Eu	Eu1'	Eu1'
NY0140192	Native range (NY)	Lund et al. (2017)	Eu	Eu1'	Eu1'
NY0140193	Native range (NY)	Lund et al. (2017)	Eu	Eu1'	Eu1'

Sample name	Origin	Source	Genetic group at K=2	Genetic group at K=3	Genetic group at K=5
NY0150201	Native range (NY)	Lund et al. (2017)	NR	admixed	NR1
NY0160211	Native range (NY)	Lund et al. (2017)	Eu	admixed	admixed
NY0160212	Native range (NY)	Lund et al. (2017)	Eu	admixed	admixed
NY0160213	Native range (NY)	Lund et al. (2017)	Eu	Eu1'	Eu1'
NY0170221	Native range (NY)	Lund et al. (2017)	Eu	admixed	admixed
NY0180231	Native range (NY)	Lund et al. (2017)	Eu	Eu1'	Eu1'
NY0180232	Native range (NY)	Lund et al. (2017)	Eu	Eu1'	Eu1'
NY0200251	Native range (NY)	Lund et al. (2017)	Eu	Eu1'	Eu1'
NY0210261	Native range (NY)	Lund et al. (2017)	Eu	Eu1'	Eu1'
NY0220271	Native range (NY)	Lund et al. (2017)	Eu	Eu1'	Eu1'
NY0220272	Native range (NY)	Lund et al. (2017)	Eu	Eu1'	Eu1'
NY0230282	Native range (NY)	Lund et al. (2017)	Eu	Eu1'	Eu1'
NY0230283	Native range (NY)	Lund et al. (2017)	Eu	admixed	admixed
NY0240291	Native range (NY)	Lund et al. (2017)	Eu	Eu1'	Eu1'
NY0240292	Native range (NY)	Lund et al. (2017)	Eu	Eu1'	Eu1'
NY0250301	Native range (NY)	Lund et al. (2017)	Eu	Eu1'	Eu1'
NY0250302	Native range (NY)	Lund et al. (2017)	Eu	Eu1'	Eu1'
OH0280351	Native range (OH)	Lund et al. (2017)	NR	NR	NR2
OH0280361	Native range (OH)	Lund et al. (2017)	NR	NR	NR2
OH0280362	Native range (OH)	Lund et al. (2017)	NR	NR	NR2
OH0280363	Native range (OH)	Lund et al. (2017)	NR	NR	NR2
OK0811331	Native range (OK)	Lund et al. (2017)	NR	NR	NR2
OK0811332	Native range (OK)	Lund et al. (2017)	NR	NR	NR2
OK0811333	Native range (OK)	Lund et al. (2017)	NR	NR	NR2
OK0821343	Native range (OK)	Lund et al. (2017)	NR	NR	NR2
PA0260311	Native range (PA)	Lund et al. (2017)	Eu	Eu1'	Eu1'
PA0270321	Native range (PA)	Lund et al. (2017)	Eu	Eu1'	Eu1'
PA0270322	Native range (PA)	Lund et al. (2017)	Eu	Eu1'	Eu1'

Sample name	Origin	Source	Genetic group at K=2	Genetic group at K=3	Genetic group at K=5
PA0270325	Native range (PA)	Lund et al. (2017)	Eu	Eu1'	Eu1'
PA0270331	Native range (PA)	Lund et al. (2017)	Eu	Eu1'	admixed
PA0270332	Native range (PA)	Lund et al. (2017)	Eu	Eu1'	Eu1'
PA0270333	Native range (PA)	Lund et al. (2017)	Eu	Eu1'	Eu1'
PA0270341	Native range (PA)	Lund et al. (2017)	Eu	Eu1'	admixed
SD0360451	Native range (SD)	Lund et al. (2017)	Eu	Eu1'	Eu1'
SD0360452	Native range (SD)	Lund et al. (2017)	admixed	Eu1'	admixed
SD0360461	Native range (SD)	Lund et al. (2017)	Eu	Eu1'	admixed
SD0360462	Native range (SD)	Lund et al. (2017)	NR	admixed	NR1
SD0370471	Native range (SD)	Lund et al. (2017)	Eu	Eu1'	admixed
SD0370472	Native range (SD)	Lund et al. (2017)	admixed	Eu1'	admixed
SD0370473	Native range (SD)	Lund et al. (2017)	admixed	Eu1'	admixed
SD0370481	Native range (SD)	Lund et al. (2017)	admixed	admixed	admixed
SD0370482	Native range (SD)	Lund et al. (2017)	Eu	Eu1'	Eu1'
SD0370483	Native range (SD)	Lund et al. (2017)	admixed	Eu1'	admixed
SD0370491	Native range (SD)	Lund et al. (2017)	Eu	Eu1'	Eu1'
SD0370492	Native range (SD)	Lund et al. (2017)	NR	admixed	admixed
SD0370502	Native range (SD)	Lund et al. (2017)	admixed	admixed	NR3
SD0370503	Native range (SD)	Lund et al. (2017)	Eu	Eu1'	Eu1'
TN0580881	Native range (TN)	Lund et al. (2017)	admixed	admixed	NR2
TN0580891	Native range (TN)	Lund et al. (2017)	NR	NR	NR2
TN0580892	Native range (TN)	Lund et al. (2017)	NR	NR	NR2
TN0580893	Native range (TN)	Lund et al. (2017)	NR	NR	NR2
TN0580901	Native range (TN)	Lund et al. (2017)	NR	NR	NR2
TN0590911	Native range (TN)	Lund et al. (2017)	NR	NR	NR2
TN0590913	Native range (TN)	Lund et al. (2017)	NR	NR	NR2
TN0590914	Native range (TN)	Lund et al. (2017)	NR	NR	NR2
TN0590921	Native range (TN)	Lund et al. (2017)	NR	NR	NR2

Sample name	Origin	Source	Genetic group at K=2	Genetic group at K=3	Genetic group at K=5
TN0590923	Native range (TN)	Lund et al. (2017)	NR	NR	NR2
TN0590925	Native range (TN)	Lund et al. (2017)	NR	NR	NR2
TN0590931	Native range (TN)	Lund et al. (2017)	NR	NR	NR2
TN0590932	Native range (TN)	Lund et al. (2017)	NR	NR	NR2
TN0590934	Native range (TN)	Lund et al. (2017)	NR	NR	NR2
TN0600941	Native range (TN)	Lund et al. (2017)	NR	NR	NR2
TN0600952	Native range (TN)	Lund et al. (2017)	NR	NR	NR2
TN0600954	Native range (TN)	Lund et al. (2017)	NR	NR	NR2
TN0610961	Native range (TN)	Lund et al. (2017)	NR	admixed	NR1
TN0610962	Native range (TN)	Lund et al. (2017)	NR	NR	NR1
TN0610963	Native range (TN)	Lund et al. (2017)	NR	NR	NR1
TN0610964	Native range (TN)	Lund et al. (2017)	NR	NR	NR1
TN0620971	Native range (TN)	Lund et al. (2017)	NR	NR	NR2
TN0620972	Native range (TN)	Lund et al. (2017)	admixed	admixed	admixed
TN0620974	Native range (TN)	Lund et al. (2017)	NR	NR	NR2
TN0630983	Native range (TN)	Lund et al. (2017)	NR	NR	NR2
TN0630991	Native range (TN)	Lund et al. (2017)	NR	NR	NR2
TN0630992	Native range (TN)	Lund et al. (2017)	NR	NR	NR2
TN0630993	Native range (TN)	Lund et al. (2017)	NR	NR	NR2
TN0641001	Native range (TN)	Lund et al. (2017)	NR	NR	NR2
TN0641011	Native range (TN)	Lund et al. (2017)	NR	NR	admixed
TN0641012	Native range (TN)	Lund et al. (2017)	NR	NR	NR2
TN0641014	Native range (TN)	Lund et al. (2017)	NR	NR	NR2
TN0651021	Native range (TN)	Lund et al. (2017)	NR	NR	NR2
TN0651022	Native range (TN)	Lund et al. (2017)	NR	NR	admixed
TN0661031	Native range (TN)	Lund et al. (2017)	NR	NR	NR2
TN0661033	Native range (TN)	Lund et al. (2017)	NR	NR	NR2
TN0661034	Native range (TN)	Lund et al. (2017)	NR	NR	admixed

Sample name	Origin	Source	Genetic group at K=2	Genetic group at K=3	Genetic group at K=5
TN0661035	Native range (TN)	Lund et al. (2017)	NR	NR	NR2
TN0661041	Native range (TN)	Lund et al. (2017)	NR	NR	NR2
TN0661044	Native range (TN)	Lund et al. (2017)	NR	NR	NR2
TN0661051	Native range (TN)	Lund et al. (2017)	NR	NR	NR2
TN0671062	Native range (TN)	Lund et al. (2017)	NR	NR	NR2
TN0671063	Native range (TN)	Lund et al. (2017)	NR	NR	NR2
TN0671064	Native range (TN)	Lund et al. (2017)	NR	NR	NR2
TX0831355	Native range (TX)	Lund et al. (2017)	NR	NR	NR2
TX0841361	Native range (TX)	Lund et al. (2017)	NR	NR	NR2
TX0841362	Native range (TX)	Lund et al. (2017)	NR	NR	NR2
TX0841363	Native range (TX)	Lund et al. (2017)	NR	NR	NR2
TX0841365	Native range (TX)	Lund et al. (2017)	NR	NR	NR2
TX0841371	Native range (TX)	Lund et al. (2017)	NR	NR	NR2
TX0851383	Native range (TX)	Lund et al. (2017)	NR	NR	NR2
TX0851391	Native range (TX)	Lund et al. (2017)	NR	admixed	admixed
TX0851392	Native range (TX)	Lund et al. (2017)	NR	NR	NR2
URU1081771	Introduced region (URU)	Lund et al. (2017)	Eu	admixed	admixed
URU1081781	Introduced region (URU)	Lund et al. (2017)	Eu	Eu1'	Eu1'
URU1081791	Introduced region (URU)	Lund et al. (2017)	Eu	Eu1'	Eu1'
URU1081801	Introduced region (URU)	Lund et al. (2017)	Eu	Eu1'	admixed
UT1051611	Native range (UT)	Lund et al. (2017)	NR	NR	admixed
UT1051613	Native range (UT)	Lund et al. (2017)	NR	NR	NR2
VA0410561	Native range (VA)	Lund et al. (2017)	NR	NR	NR1
VA0420571	Native range (VA)	Lund et al. (2017)	NR	NR	NR2
VA0420581	Native range (VA)	Lund et al. (2017)	NR	NR	NR2
VA0420582	Native range (VA)	Lund et al. (2017)	NR	NR	admixed
VA0420591	Native range (VA)	Lund et al. (2017)	NR	NR	admixed
VA0420592	Native range (VA)	Lund et al. (2017)	NR	NR	admixed

Sample name	Origin	Source	Genetic group at K=2	Genetic group at K=3	Genetic group at K=5
VA0430601	Native range (VA)	Lund et al. (2017)	NR	NR	admixed
VA0430611	Native range (VA)	Lund et al. (2017)	NR	NR	admixed
VA0430612	Native range (VA)	Lund et al. (2017)	admixed	admixed	admixed
VA0440621	Native range (VA)	Lund et al. (2017)	NR	NR	NR1
VA0440631	Native range (VA)	Lund et al. (2017)	NR	NR	NR1
VA0440632	Native range (VA)	Lund et al. (2017)	NR	NR	NR1
VA0440641	Native range (VA)	Lund et al. (2017)	NR	NR	admixed
VA0450661	Native range (VA)	Lund et al. (2017)	NR	NR	NR1
VA0450662	Native range (VA)	Lund et al. (2017)	NR	NR	NR1
VA0450671	Native range (VA)	Lund et al. (2017)	NR	NR	NR1
VA0450672	Native range (VA)	Lund et al. (2017)	NR	NR	admixed
VA0460681	Native range (VA)	Lund et al. (2017)	admixed	admixed	NR1
VA0460682	Native range (VA)	Lund et al. (2017)	NR	NR	NR1
VA0470691	Native range (VA)	Lund et al. (2017)	admixed	admixed	NR1
VA0480711	Native range (VA)	Lund et al. (2017)	NR	NR	admixed
VA0480712	Native range (VA)	Lund et al. (2017)	NR	NR	NR1
VA0490721	Native range (VA)	Lund et al. (2017)	NR	NR	NR1
VA0490723	Native range (VA)	Lund et al. (2017)	NR	NR	NR1
VA0490725	Native range (VA)	Lund et al. (2017)	NR	NR	NR1
VA0490732	Native range (VA)	Lund et al. (2017)	NR	NR	NR1
VA0490741	Native range (VA)	Lund et al. (2017)	NR	NR	NR1
VA0490742	Native range (VA)	Lund et al. (2017)	NR	NR	NR1
VA0490743	Native range (VA)	Lund et al. (2017)	NR	NR	NR1
WV0380511	Native range (WV)	Lund et al. (2017)	NR	NR	NR2
WV0380512	Native range (WV)	Lund et al. (2017)	NR	NR	NR2
WV0380513	Native range (WV)	Lund et al. (2017)	NR	NR	NR2
WV0390521	Native range (WV)	Lund et al. (2017)	NR	NR	NR2
WV0400531	Native range (WV)	Lund et al. (2017)	NR	NR	NR2

Sample name	Origin	Source	Genetic group at K=2	Genetic group at K=3	Genetic group at K=5
WV0400532	Native range (WV)	Lund et al. (2017)	NR	NR	NR2
WV0400541	Native range (WV)	Lund et al. (2017)	NR	NR	NR2
WV0400542	Native range (WV)	Lund et al. (2017)	NR	NR	NR2
WV0400543	Native range (WV)	Lund et al. (2017)	NR	NR	NR2
WV0400551	Native range (WV)	Lund et al. (2017)	NR	NR	NR2
WV0400552	Native range (WV)	Lund et al. (2017)	NR	NR	NR2
WV0400553	Native range (WV)	Lund et al. (2017)	NR	NR	NR2
WV0400554	Native range (WV)	Lund et al. (2017)	NR	NR	NR2

Supplementary Table S3. List of the 1173 grape phylloxera individuals included in this study. For each sample, we indicate the sampling site (country), host plant and grape phylloxera feeding form.

Sample code	Sampling location (lat, long)	Sampling year	Country[1]	Host plant	Phylloxera feeding form
AUT001	48.41, 15.61	2018	AUT	*Vitis vinifera* L. cultivar	Leaf-feeding form
AUT002	48.41, 15.61	2018	AUT	*Vitis vinifera* L. cultivar	Leaf-feeding form
AUT003	48.41, 15.61	2018	AUT	*Vitis vinifera* L. cultivar	Leaf-feeding form
AUT004	48.41, 15.61	2018	AUT	*Vitis vinifera* L. cultivar	Leaf-feeding form
AUT005	48.41, 15.61	2018	AUT	*Vitis vinifera* L. cultivar	Leaf-feeding form
AUT006	48.41, 15.61	2018	AUT	*Vitis* spp. interspecific resistant grape variety	Leaf-feeding form
AUT007	48.41, 15.61	2018	AUT	*Vitis* spp. interspecific resistant grape variety	Leaf-feeding form
AUT008	48.41, 15.61	2018	AUT	*Vitis* spp. interspecific resistant grape variety	Leaf-feeding form
AUT009	48.41, 15.61	2018	AUT	*Vitis* spp. interspecific resistant grape variety	Leaf-feeding form
AUT010	48.41, 15.61	2018	AUT	*Vitis vinifera* L. cultivar	Leaf-feeding form
AUT011	48.41, 15.61	2018	AUT	*Vitis vinifera* L. cultivar	Leaf-feeding form
AUT012	48.41, 15.61	2018	AUT	*Vitis vinifera* L. cultivar	Leaf-feeding form
AUT013	48.41, 15.61	2018	AUT	*Vitis vinifera* L. cultivar	Leaf-feeding form
AUT014	48.41, 15.61	2018	AUT	*Vitis vinifera* L. cultivar	Leaf-feeding form
AUT015	48.41, 15.61	2018	AUT	*Vitis* spp. interspecific resistant grape variety	Leaf-feeding form
AUT016	48.41, 15.61	2018	AUT	*Vitis* spp. interspecific resistant grape variety	Leaf-feeding form
AUT017	48.41, 15.61	2018	AUT	*Vitis* spp. interspecific resistant grape variety	Leaf-feeding form
AUT018	48.32, 16.07	2018	AUT	*Vitis* spp. interspecific rootstock	Leaf-feeding form
AUT019	48.32, 16.07	2018	AUT	*Vitis* spp. interspecific rootstock	Leaf-feeding form
AUT020	48.32, 16.07	2018	AUT	*Vitis* spp. interspecific rootstock	Leaf-feeding form
AUT021	48.32, 16.07	2019	AUT	*Vitis* spp. interspecific rootstock	Leaf-feeding form
AUT022	48.32, 16.07	2019	AUT	*Vitis* spp. interspecific rootstock	Leaf-feeding form
AUT023	48.32, 16.07	2019	AUT	*Vitis* spp. interspecific rootstock	Leaf-feeding form
AUT024	48.37, 16.41	2012	AUT	*Vitis* spp. interspecific rootstock	Leaf-feeding form
AUT025	48.37, 16.41	2012	AUT	*Vitis* spp. interspecific rootstock	Leaf-feeding form

Sample code	Sampling location (lat, long)	Sampling year	Country[1]	Host plant	Phylloxera feeding form
AUT026	48.37, 16.41	2012	AUT	*Vitis* spp. interspecific rootstock	Leaf-feeding form
AUT027	48.37, 16.41	2012	AUT	*Vitis* spp. interspecific rootstock	Leaf-feeding form
AUT028	48.37, 16.41	2012	AUT	*Vitis* spp. interspecific rootstock	Leaf-feeding form
AUT029	48.37, 16.41	2012	AUT	*Vitis* spp. interspecific rootstock	Leaf-feeding form
AUT030	48.37, 16.41	2012	AUT	*Vitis* spp. interspecific rootstock	Leaf-feeding form
AUT031	48.37, 16.41	2012	AUT	*Vitis* spp. interspecific rootstock	Leaf-feeding form
AUT032	48.37, 16.41	2012	AUT	*Vitis* spp. interspecific rootstock	Leaf-feeding form
AUT033	48.37, 16.41	2012	AUT	*Vitis* spp. interspecific rootstock	Leaf-feeding form
AUT034	48.37, 16.41	2012	AUT	*Vitis* spp. interspecific rootstock	Leaf-feeding form
AUT035	48.37, 16.41	2012	AUT	*Vitis* spp. interspecific rootstock	Leaf-feeding form
AUT036	48.37, 16.41	2012	AUT	*Vitis* spp. interspecific rootstock	Leaf-feeding form
AUT037	48.37, 16.41	2012	AUT	*Vitis* spp. interspecific rootstock	Leaf-feeding form
AUT038	48.37, 16.41	2012	AUT	*Vitis* spp. interspecific rootstock	Leaf-feeding form
AUT039	48.37, 16.41	2012	AUT	*Vitis* spp. interspecific rootstock	Leaf-feeding form
AUT040	48.37, 16.41	2012	AUT	*Vitis* spp. interspecific rootstock	Leaf-feeding form
AUT041	48.37, 16.41	2012	AUT	*Vitis* spp. interspecific rootstock	Leaf-feeding form
AUT042	48.37, 16.41	2012	AUT	*Vitis* spp. interspecific rootstock	Leaf-feeding form
AUT043	48.37, 16.41	2012	AUT	*Vitis* spp. interspecific rootstock	Leaf-feeding form
AUT044	48.37, 16.41	2012	AUT	*Vitis* spp. interspecific rootstock	Leaf-feeding form
AUT045	48.37, 16.41	2012	AUT	*Vitis* spp. interspecific rootstock	Leaf-feeding form
AUT046	48.37, 16.41	2012	AUT	*Vitis* spp. interspecific rootstock	Leaf-feeding form
AUT047	48.37, 16.41	2012	AUT	*Vitis* spp. interspecific rootstock	Leaf-feeding form
AUT048	48.37, 16.41	2012	AUT	*Vitis* spp. interspecific rootstock	Leaf-feeding form
AUT049	48.37, 16.41	2012	AUT	*Vitis* spp. interspecific rootstock	Leaf-feeding form
AUT050	48.37, 16.41	2012	AUT	*Vitis* spp. interspecific rootstock	Leaf-feeding form
AUT051	48.37, 16.41	2012	AUT	*Vitis* spp. interspecific rootstock	Leaf-feeding form
AUT052	48.37, 16.41	2012	AUT	*Vitis* spp. interspecific rootstock	Leaf-feeding form

Sample code	Sampling location (lat, long)	Sampling year	Country[1]	Host plant	Phylloxera feeding form
AUT053	48.37, 16.41	2012	AUT	*Vitis* spp. interspecific rootstock	Leaf-feeding form
AUT054	48.31, 16.37	n.a.	AUT	*Vitis* spp. interspecific rootstock	Leaf-feeding form
AUT055	48.31, 16.37	n.a.	AUT	*Vitis* spp. interspecific rootstock	Leaf-feeding form
AUT056	48.31, 16.37	n.a.	AUT	*Vitis* spp. interspecific rootstock	Leaf-feeding form
AUT057	48.31, 16.37	n.a.	AUT	*Vitis* spp. interspecific rootstock	Leaf-feeding form
AUT058	48.31, 16.37	n.a.	AUT	*Vitis* spp. interspecific rootstock	Leaf-feeding form
AUT059	48.31, 16.37	n.a.	AUT	*Vitis* spp. interspecific rootstock	Leaf-feeding form
AUT060	48.31, 16.37	n.a.	AUT	*Vitis* spp. interspecific rootstock	Leaf-feeding form
AUT061	48.31, 16.37	n.a.	AUT	*Vitis* spp. interspecific rootstock	Leaf-feeding form
AUT062	48.31, 16.37	n.a.	AUT	*Vitis* spp. interspecific rootstock	Leaf-feeding form
AUT063	48.31, 16.37	n.a.	AUT	*Vitis* spp. interspecific rootstock	Leaf-feeding form
AUT064	48.31, 16.37	n.a.	AUT	*Vitis* spp. interspecific rootstock	Leaf-feeding form
AUT065	48.31, 16.37	n.a.	AUT	*Vitis* spp. interspecific rootstock	Leaf-feeding form
AUT066	48.31, 16.37	n.a.	AUT	*Vitis* spp. interspecific rootstock	Leaf-feeding form
AUT067	48.31, 16.37	n.a.	AUT	*Vitis* spp. interspecific rootstock	Leaf-feeding form
AUT068	48.31, 16.37	n.a.	AUT	*Vitis* spp. interspecific rootstock	Leaf-feeding form
AUT069	48.31, 16.37	n.a.	AUT	*Vitis* spp. interspecific rootstock	Leaf-feeding form
AUT070	48.31, 16.37	n.a.	AUT	*Vitis* spp. interspecific rootstock	Leaf-feeding form
AUT071	48.31, 16.37	n.a.	AUT	*Vitis* spp. interspecific rootstock	Leaf-feeding form
AUT072	48.31, 16.37	n.a.	AUT	*Vitis* spp. interspecific rootstock	Leaf-feeding form
AUT073	48.31, 16.37	n.a.	AUT	*Vitis* spp. interspecific rootstock	Leaf-feeding form
AUT074	48.31, 16.37	n.a.	AUT	*Vitis* spp. interspecific rootstock	Leaf-feeding form
AUT075	48.31, 16.37	n.a.	AUT	*Vitis* spp. interspecific rootstock	Leaf-feeding form
AUT076	48.31, 16.37	n.a.	AUT	*Vitis* spp. interspecific rootstock	Leaf-feeding form
AUT077	48.31, 16.37	n.a.	AUT	*Vitis* spp. interspecific rootstock	Leaf-feeding form
AUT078	48.31, 16.37	n.a.	AUT	*Vitis* spp. interspecific rootstock	Leaf-feeding form
AUT079	48.31, 16.37	n.a.	AUT	*Vitis* spp. interspecific rootstock	Leaf-feeding form

Sample code	Sampling location (lat, long)	Sampling year	Country[1]	Host plant	Phylloxera feeding form
AUT080	48.31, 16.37	n.a.	AUT	*Vitis* spp. interspecific rootstock	Leaf-feeding form
AUT081	48.31, 16.37	n.a.	AUT	*Vitis* spp. interspecific rootstock	Leaf-feeding form
AUT082	47.76, 16.66	2015	AUT	*Vitis* spp. interspecific rootstock	Leaf-feeding form
AUT083	47.76, 16.66	2015	AUT	*Vitis* spp. interspecific rootstock	Leaf-feeding form
AUT084	47.76, 16.66	2015	AUT	*Vitis* spp. interspecific rootstock	Leaf-feeding form
AUT085	47.76, 16.66	2015	AUT	*Vitis* spp. interspecific rootstock	Leaf-feeding form
AUT086	47.76, 16.66	2015	AUT	*Vitis* spp. interspecific rootstock	Leaf-feeding form
AUT087	47.76, 16.66	2015	AUT	*Vitis* spp. interspecific rootstock	Leaf-feeding form
AUT088	47.76, 16.66	2015	AUT	*Vitis* spp. interspecific rootstock	Leaf-feeding form
AUT089	47.76, 16.66	2015	AUT	*Vitis* spp. interspecific rootstock	Leaf-feeding form
AUT090	47.76, 16.66	2015	AUT	*Vitis* spp. interspecific rootstock	Leaf-feeding form
AUT091	47.76, 16.66	2015	AUT	*Vitis* spp. interspecific rootstock	Leaf-feeding form
AUT092	47.76, 16.66	2015	AUT	*Vitis* spp. interspecific rootstock	Leaf-feeding form
AUT093	47.76, 16.66	2015	AUT	*Vitis* spp. interspecific rootstock	Leaf-feeding form
AUT094	47.76, 16.66	2015	AUT	*Vitis* spp. interspecific rootstock	Leaf-feeding form
AUT095	48.35, 15.75	2015	AUT	*Vitis* spp. interspecific rootstock	Leaf-feeding form
AUT096	48.35, 15.75	2015	AUT	*Vitis* spp. interspecific rootstock	Leaf-feeding form
AUT097	48.35, 15.75	2015	AUT	*Vitis* spp. interspecific rootstock	Leaf-feeding form
AUT098	48.35, 15.75	2015	AUT	*Vitis* spp. interspecific rootstock	Leaf-feeding form
AUT099	48.35, 15.75	2015	AUT	*Vitis* spp. interspecific rootstock	Leaf-feeding form
AUT100	48.35, 15.75	2015	AUT	*Vitis* spp. interspecific rootstock	Leaf-feeding form
AUT101	48.35, 15.75	2015	AUT	*Vitis* spp. interspecific rootstock	Leaf-feeding form
AUT102	48.35, 15.75	2015	AUT	*Vitis* spp. interspecific rootstock	Leaf-feeding form
AUT103	48.35, 15.75	2015	AUT	*Vitis* spp. interspecific rootstock	Leaf-feeding form
AUT104	48.35, 15.75	2015	AUT	*Vitis* spp. interspecific rootstock	Leaf-feeding form
AUT105	48.35, 15.75	2015	AUT	*Vitis* spp. interspecific rootstock	Leaf-feeding form
AUT106	48.35, 15.75	2015	AUT	*Vitis* spp. interspecific rootstock	Leaf-feeding form

Sample code	Sampling location (lat, long)	Sampling year	Country[1]	Host plant	Phylloxera feeding form
AUT107	48.35, 15.75	2015	AUT	*Vitis* spp. interspecific rootstock	Leaf-feeding form
AUT108	48.35, 15.75	2015	AUT	*Vitis* spp. interspecific rootstock	Leaf-feeding form
AUT109	48.35, 15.75	2015	AUT	*Vitis* spp. interspecific rootstock	Leaf-feeding form
AUT110	48.35, 15.75	2015	AUT	*Vitis* spp. interspecific rootstock	Leaf-feeding form
AUT111	48.35, 15.75	2015	AUT	*Vitis* spp. interspecific rootstock	Leaf-feeding form
AUT112	48.35, 15.75	2015	AUT	*Vitis* spp. interspecific rootstock	Leaf-feeding form
AUT113	48.35, 15.75	2015	AUT	*Vitis* spp. interspecific rootstock	Leaf-feeding form
AUT114	48.35, 15.75	2015	AUT	*Vitis* spp. interspecific rootstock	Leaf-feeding form
AUT115	48.35, 15.75	2015	AUT	*Vitis* spp. interspecific rootstock	Leaf-feeding form
AUT116	48.35, 15.75	2015	AUT	*Vitis* spp. interspecific rootstock	Leaf-feeding form
AUT117	48.35, 15.75	2015	AUT	*Vitis* spp. interspecific rootstock	Leaf-feeding form
AUT118	48.35, 15.75	2015	AUT	*Vitis* spp. interspecific rootstock	Leaf-feeding form
AUT119	48.35, 15.75	2015	AUT	*Vitis* spp. interspecific rootstock	Leaf-feeding form
AUT120	48.35, 15.75	2015	AUT	*Vitis* spp. interspecific rootstock	Leaf-feeding form
AUT121	48.35, 15.75	2015	AUT	*Vitis* spp. interspecific rootstock	Leaf-feeding form
AUT122	48.32, 15.73	2015	AUT	*Vitis* spp. interspecific rootstock	Leaf-feeding form
AUT123	48.32, 15.73	2015	AUT	*Vitis* spp. interspecific rootstock	Leaf-feeding form
AUT124	48.32, 15.73	2015	AUT	*Vitis* spp. interspecific rootstock	Leaf-feeding form
AUT125	48.32, 15.73	2015	AUT	*Vitis* spp. interspecific rootstock	Leaf-feeding form
AUT126	48.32, 15.73	2015	AUT	*Vitis* spp. interspecific rootstock	Leaf-feeding form
AUT127	48.32, 15.73	2015	AUT	*Vitis* spp. interspecific rootstock	Leaf-feeding form
AUT128	48.32, 15.73	2015	AUT	*Vitis* spp. interspecific rootstock	Leaf-feeding form
AUT129	48.32, 15.73	2015	AUT	*Vitis* spp. interspecific rootstock	Leaf-feeding form
AUT130	48.32, 15.73	2015	AUT	*Vitis* spp. interspecific rootstock	Leaf-feeding form
AUT131	48.32, 15.73	2015	AUT	*Vitis* spp. interspecific rootstock	Leaf-feeding form
AUT132	48.32, 15.73	2015	AUT	*Vitis* spp. interspecific rootstock	Leaf-feeding form
AUT133	48.32, 15.73	2015	AUT	*Vitis* spp. interspecific rootstock	Leaf-feeding form

Sample code	Sampling location (lat, long)	Sampling year	Country[1]	Host plant	Phylloxera feeding form
AUT134	48.32, 15.73	2015	AUT	*Vitis* spp. interspecific rootstock	Leaf-feeding form
AUT135	48.32, 15.73	2015	AUT	*Vitis* spp. interspecific rootstock	Leaf-feeding form
AUT136	48.32, 15.73	2015	AUT	*Vitis* spp. interspecific rootstock	Leaf-feeding form
AUT137	48.32, 15.73	2015	AUT	*Vitis* spp. interspecific rootstock	Leaf-feeding form
AUT138	48.32, 15.73	2015	AUT	*Vitis* spp. interspecific rootstock	Leaf-feeding form
AUT139	48.32, 15.73	2015	AUT	*Vitis* spp. interspecific rootstock	Leaf-feeding form
AUT140	48.32, 15.73	2015	AUT	*Vitis* spp. interspecific rootstock	Leaf-feeding form
AUT141	48.32, 15.73	2015	AUT	*Vitis* spp. interspecific rootstock	Leaf-feeding form
AUT142	48.32, 15.73	2015	AUT	*Vitis* spp. interspecific rootstock	Leaf-feeding form
AUT143	48.32, 15.73	2015	AUT	*Vitis* spp. interspecific rootstock	Leaf-feeding form
AUT144	48.32, 15.73	2015	AUT	*Vitis* spp. interspecific rootstock	Leaf-feeding form
AUT145	48.51, 15.67	2015	AUT	*Vitis* spp. interspecific rootstock	Leaf-feeding form
AUT146	48.51, 15.67	2015	AUT	*Vitis* spp. interspecific rootstock	Leaf-feeding form
AUT147	48.51, 15.67	2015	AUT	*Vitis* spp. interspecific rootstock	Leaf-feeding form
AUT148	48.51, 15.67	2015	AUT	*Vitis* spp. interspecific rootstock	Leaf-feeding form
AUT149	48.51, 15.67	2015	AUT	*Vitis* spp. interspecific rootstock	Leaf-feeding form
AUT150	48.51, 15.67	2015	AUT	*Vitis* spp. interspecific rootstock	Leaf-feeding form
AUT151	48.51, 15.67	2015	AUT	*Vitis* spp. interspecific rootstock	Leaf-feeding form
AUT152	48.49, 15.68	2015	AUT	*Vitis* spp. interspecific rootstock	Leaf-feeding form
AUT153	48.49, 15.68	2015	AUT	*Vitis* spp. interspecific rootstock	Leaf-feeding form
AUT154	48.49, 15.68	2015	AUT	*Vitis* spp. interspecific rootstock	Leaf-feeding form
AUT155	48.49, 15.68	2015	AUT	*Vitis* spp. interspecific rootstock	Leaf-feeding form
AUT156	48.49, 15.68	2015	AUT	*Vitis* spp. interspecific rootstock	Leaf-feeding form
AUT157	48.49, 15.68	2015	AUT	*Vitis* spp. interspecific rootstock	Leaf-feeding form
AUT158	48.49, 15.68	2015	AUT	*Vitis* spp. interspecific rootstock	Leaf-feeding form
AUT159	48.49, 15.68	2015	AUT	*Vitis* spp. interspecific rootstock	Leaf-feeding form
AUT160	48.49, 15.68	2015	AUT	*Vitis* spp. interspecific rootstock	Leaf-feeding form

Sample code	Sampling location (lat, long)	Sampling year	Country[1]	Host plant	Phylloxera feeding form
AUT161	48.49, 15.68	2015	AUT	*Vitis* spp. interspecific rootstock	Leaf-feeding form
AUT162	48.49, 15.68	2015	AUT	*Vitis* spp. interspecific rootstock	Leaf-feeding form
AUT163	48.49, 15.68	2015	AUT	*Vitis* spp. interspecific rootstock	Leaf-feeding form
AUT164	48.49, 15.68	2015	AUT	*Vitis* spp. interspecific rootstock	Leaf-feeding form
AUT165	48.49, 15.68	2015	AUT	*Vitis* spp. interspecific rootstock	Leaf-feeding form
AUT166	48.49, 15.68	2015	AUT	*Vitis* spp. interspecific rootstock	Leaf-feeding form
AUT167	48.49, 15.68	2015	AUT	*Vitis* spp. interspecific rootstock	Leaf-feeding form
AUT168	48.49, 15.68	2015	AUT	*Vitis* spp. interspecific rootstock	Leaf-feeding form
AUT169	48.49, 15.68	2015	AUT	*Vitis* spp. interspecific rootstock	Leaf-feeding form
AUT170	48.49, 15.68	2015	AUT	*Vitis* spp. interspecific rootstock	Leaf-feeding form
AUT171	48.49, 15.68	2015	AUT	*Vitis* spp. interspecific rootstock	Leaf-feeding form
AUT172	48.49, 15.68	2015	AUT	*Vitis* spp. interspecific rootstock	Leaf-feeding form
AUT173	48.49, 15.68	2015	AUT	*Vitis* spp. interspecific rootstock	Leaf-feeding form
AUT174	48.49, 15.68	2015	AUT	*Vitis* spp. interspecific rootstock	Leaf-feeding form
AUT175	48.49, 15.68	2015	AUT	*Vitis* spp. interspecific rootstock	Leaf-feeding form
AUT176	48.49, 15.68	2015	AUT	*Vitis* spp. interspecific rootstock	Leaf-feeding form
AUT177	48.49, 15.68	2015	AUT	*Vitis* spp. interspecific rootstock	Leaf-feeding form
AUT178	48.49, 15.68	2015	AUT	*Vitis* spp. interspecific rootstock	Leaf-feeding form
AUT179	48.49, 15.68	2015	AUT	*Vitis* spp. interspecific rootstock	Leaf-feeding form
AUT180	48.49, 15.68	2015	AUT	*Vitis* spp. interspecific rootstock	Leaf-feeding form
AUT181	48.49, 15.68	2015	AUT	*Vitis* spp. interspecific rootstock	Leaf-feeding form
AUT182	48.49, 15.68	2015	AUT	*Vitis* spp. interspecific rootstock	Leaf-feeding form
AUT183	48.49, 15.68	2015	AUT	*Vitis* spp. interspecific rootstock	Leaf-feeding form
AUT184	48.49, 15.68	2015	AUT	*Vitis* spp. interspecific rootstock	Leaf-feeding form
AUT185	48.49, 15.68	2015	AUT	*Vitis* spp. interspecific rootstock	Leaf-feeding form
AUT186	48.49, 15.68	2015	AUT	*Vitis* spp. interspecific rootstock	Leaf-feeding form
AUT187	48.49, 15.68	2015	AUT	*Vitis* spp. interspecific rootstock	Leaf-feeding form

Sample code	Sampling location (lat, long)	Sampling year	Country[1]	Host plant	Phylloxera feeding form
AUT188	48.49, 15.68	2015	AUT	*Vitis* spp. interspecific rootstock	Leaf-feeding form
AUT189	48.49, 15.68	2015	AUT	*Vitis* spp. interspecific rootstock	Leaf-feeding form
AUT190	48.49, 15.68	2015	AUT	*Vitis* spp. interspecific rootstock	Leaf-feeding form
AUT191	48.49, 15.68	2015	AUT	*Vitis* spp. interspecific rootstock	Leaf-feeding form
AUT192	48.49, 15.68	2015	AUT	*Vitis* spp. interspecific rootstock	Leaf-feeding form
AUT193	48.49, 15.68	2015	AUT	*Vitis* spp. interspecific rootstock	Leaf-feeding form
AUT194	48.49, 15.68	2015	AUT	*Vitis* spp. interspecific rootstock	Leaf-feeding form
AUT195	48.49, 15.68	2015	AUT	*Vitis* spp. interspecific rootstock	Leaf-feeding form
AUT196	48.49, 15.68	2015	AUT	*Vitis* spp. interspecific rootstock	Leaf-feeding form
AUT197	48.42, 15.62	2015	AUT	*Vitis* spp. interspecific rootstock	Leaf-feeding form
AUT198	48.42, 15.62	2015	AUT	*Vitis* spp. interspecific rootstock	Leaf-feeding form
AUT199	48.42, 15.62	2015	AUT	*Vitis* spp. interspecific rootstock	Leaf-feeding form
AUT200	48.42, 15.62	2015	AUT	*Vitis* spp. interspecific rootstock	Leaf-feeding form
AUT201	48.42, 15.62	2015	AUT	*Vitis* spp. interspecific rootstock	Leaf-feeding form
AUT202	48.42, 15.62	2015	AUT	*Vitis* spp. interspecific rootstock	Leaf-feeding form
AUT203	48.42, 15.62	2015	AUT	*Vitis* spp. interspecific rootstock	Leaf-feeding form
AUT204	48.35, 15.7	2015	AUT	*Vitis* spp. interspecific rootstock	Leaf-feeding form
AUT205	48.35, 15.7	2015	AUT	*Vitis* spp. interspecific rootstock	Leaf-feeding form
AUT206	48.35, 15.7	2015	AUT	*Vitis* spp. interspecific rootstock	Leaf-feeding form
AUT207	48.35, 15.7	2015	AUT	*Vitis* spp. interspecific rootstock	Leaf-feeding form
AUT208	48.35, 15.7	2015	AUT	*Vitis* spp. interspecific rootstock	Leaf-feeding form
AUT209	48.35, 15.7	2015	AUT	*Vitis* spp. interspecific rootstock	Leaf-feeding form
AUT210	48.35, 15.7	2015	AUT	*Vitis* spp. interspecific rootstock	Leaf-feeding form
AUT211	48.35, 15.69	2015	AUT	*Vitis* spp. interspecific rootstock	Leaf-feeding form
AUT212	48.35, 15.69	2015	AUT	*Vitis* spp. interspecific rootstock	Leaf-feeding form
AUT213	48.35, 15.69	2015	AUT	*Vitis* spp. interspecific rootstock	Leaf-feeding form
AUT214	48.35, 15.69	2015	AUT	*Vitis* spp. interspecific rootstock	Leaf-feeding form

**Sample code**	**Sampling location (lat, long)**	**Sampling year**	**Country[1]**	**Host plant**	**Phylloxera feeding form**
AUT215	48.35, 15.69	2015	AUT	*Vitis* spp. interspecific rootstock	Leaf-feeding form
AUT216	48.35, 15.69	2015	AUT	*Vitis* spp. interspecific rootstock	Leaf-feeding form
AUT217	48.35, 15.69	2015	AUT	*Vitis* spp. interspecific rootstock	Leaf-feeding form
AUT218	48.35, 15.69	2015	AUT	*Vitis* spp. interspecific rootstock	Leaf-feeding form
AUT219	48.35, 15.69	2015	AUT	*Vitis* spp. interspecific rootstock	Leaf-feeding form
AUT220	48.35, 15.69	2015	AUT	*Vitis* spp. interspecific rootstock	Leaf-feeding form
AUT221	48.35, 15.69	2015	AUT	*Vitis* spp. interspecific rootstock	Leaf-feeding form
AUT222	48.35, 15.69	2015	AUT	*Vitis* spp. interspecific rootstock	Leaf-feeding form
AUT223	48.35, 15.69	2015	AUT	*Vitis* spp. interspecific rootstock	Leaf-feeding form
AUT224	48.35, 15.69	2015	AUT	*Vitis* spp. interspecific rootstock	Leaf-feeding form
AUT225	48.35, 15.69	2015	AUT	*Vitis* spp. interspecific rootstock	Leaf-feeding form
AUT226	48.35, 15.69	2015	AUT	*Vitis* spp. interspecific rootstock	Leaf-feeding form
AUT227	48.35, 15.69	2015	AUT	*Vitis* spp. interspecific rootstock	Leaf-feeding form
AUT228	48.35, 15.69	2015	AUT	*Vitis* spp. interspecific rootstock	Leaf-feeding form
AUT229	48.35, 15.69	2015	AUT	*Vitis* spp. interspecific rootstock	Leaf-feeding form
AUT230	48.35, 15.69	2015	AUT	*Vitis* spp. interspecific rootstock	Leaf-feeding form
AUT231	48.35, 15.69	2015	AUT	*Vitis* spp. interspecific rootstock	Leaf-feeding form
AUT232	48.35, 15.69	2015	AUT	*Vitis* spp. interspecific rootstock	Leaf-feeding form
AUT233	48.35, 15.69	2015	AUT	*Vitis* spp. interspecific rootstock	Leaf-feeding form
AUT234	48.35, 15.69	2015	AUT	*Vitis* spp. interspecific rootstock	Leaf-feeding form
AUT235	48.35, 15.69	2015	AUT	*Vitis* spp. interspecific rootstock	Leaf-feeding form
AUT236	48.35, 15.69	2015	AUT	*Vitis* spp. interspecific rootstock	Leaf-feeding form
AUT237	48.35, 15.69	2015	AUT	*Vitis* spp. interspecific rootstock	Leaf-feeding form
AUT238	48.35, 15.69	2015	AUT	*Vitis* spp. interspecific rootstock	Leaf-feeding form
AUT239	48.35, 15.69	2015	AUT	*Vitis* spp. interspecific rootstock	Leaf-feeding form
AUT240	48.35, 15.69	2015	AUT	*Vitis* spp. interspecific rootstock	Leaf-feeding form
AUT241	48.35, 15.69	2015	AUT	*Vitis* spp. interspecific rootstock	Leaf-feeding form

Sample code	Sampling location (lat, long)	Sampling year	Country[1]	Host plant	Phylloxera feeding form
AUT242	48.35, 15.69	2015	AUT	*Vitis* spp. interspecific rootstock	Leaf-feeding form
AUT243	48.35, 15.69	2015	AUT	*Vitis* spp. interspecific rootstock	Leaf-feeding form
AUT244	48.35, 15.69	2015	AUT	*Vitis* spp. interspecific rootstock	Leaf-feeding form
AUT245	48.35, 15.69	2015	AUT	*Vitis* spp. interspecific rootstock	Leaf-feeding form
AUT246	48.35, 15.69	2015	AUT	*Vitis* spp. interspecific rootstock	Leaf-feeding form
AUT247	48.35, 15.69	2015	AUT	*Vitis* spp. interspecific rootstock	Leaf-feeding form
AUT248	48.35, 15.69	2015	AUT	*Vitis* spp. interspecific rootstock	Leaf-feeding form
AUT249	48.35, 15.69	2015	AUT	*Vitis* spp. interspecific rootstock	Leaf-feeding form
AUT250	48.35, 15.69	2015	AUT	*Vitis* spp. interspecific rootstock	Leaf-feeding form
AUT251	48.35, 15.69	2015	AUT	*Vitis* spp. interspecific rootstock	Leaf-feeding form
AUT252	48.35, 15.69	2015	AUT	*Vitis* spp. interspecific rootstock	Leaf-feeding form
AUT253	48.35, 15.69	2015	AUT	*Vitis* spp. interspecific rootstock	Leaf-feeding form
AUT254	48.51, 16.24	2015	AUT	*Vitis* spp. interspecific rootstock	Leaf-feeding form
AUT255	48.51, 16.24	2015	AUT	*Vitis* spp. interspecific rootstock	Leaf-feeding form
AUT256	48.51, 16.24	2015	AUT	*Vitis* spp. interspecific rootstock	Leaf-feeding form
AUT257	48.51, 16.24	2015	AUT	*Vitis* spp. interspecific rootstock	Leaf-feeding form
AUT258	48.51, 16.24	2015	AUT	*Vitis* spp. interspecific rootstock	Leaf-feeding form
AUT259	48.51, 16.24	2015	AUT	*Vitis* spp. interspecific rootstock	Leaf-feeding form
AUT260	48.51, 16.24	2015	AUT	*Vitis* spp. interspecific rootstock	Leaf-feeding form
AUT261	48.51, 16.24	2015	AUT	*Vitis* spp. interspecific rootstock	Leaf-feeding form
AUT262	48.51, 16.24	2015	AUT	*Vitis* spp. interspecific rootstock	Leaf-feeding form
AUT263	48.51, 16.24	2015	AUT	*Vitis* spp. interspecific rootstock	Leaf-feeding form
AUT264	48.68, 16.62	2015	AUT	*Vitis* spp. interspecific rootstock	Leaf-feeding form
AUT265	48.68, 16.62	2015	AUT	*Vitis* spp. interspecific rootstock	Leaf-feeding form
AUT266	48.68, 16.62	2015	AUT	*Vitis* spp. interspecific rootstock	Leaf-feeding form
AUT267	48.68, 16.62	2015	AUT	*Vitis* spp. interspecific rootstock	Leaf-feeding form
AUT268	48.68, 16.62	2015	AUT	*Vitis* spp. interspecific rootstock	Leaf-feeding form

Sample code	Sampling location (lat, long)	Sampling year	Country[1]	Host plant	Phylloxera feeding form
AUT269	48.68, 16.62	2015	AUT	*Vitis* spp. interspecific rootstock	Leaf-feeding form
AUT270	48.68, 16.62	2015	AUT	*Vitis* spp. interspecific rootstock	Leaf-feeding form
AUT271	48.68, 16.62	2015	AUT	*Vitis* spp. interspecific rootstock	Leaf-feeding form
AUT272	48.68, 16.62	2015	AUT	*Vitis* spp. interspecific rootstock	Leaf-feeding form
AUT273	48.68, 16.62	2015	AUT	*Vitis* spp. interspecific rootstock	Leaf-feeding form
AUT274	48.68, 16.62	2015	AUT	*Vitis* spp. interspecific rootstock	Leaf-feeding form
AUT275	48.68, 16.62	2015	AUT	*Vitis* spp. interspecific rootstock	Leaf-feeding form
AUT276	48.68, 16.62	2015	AUT	*Vitis* spp. interspecific rootstock	Leaf-feeding form
AUT277	48.68, 16.62	2015	AUT	*Vitis* spp. interspecific rootstock	Leaf-feeding form
AUT278	48.68, 16.62	2015	AUT	*Vitis* spp. interspecific rootstock	Leaf-feeding form
AUT279	48.68, 16.62	2015	AUT	*Vitis* spp. interspecific rootstock	Leaf-feeding form
AUT280	48.68, 16.62	2015	AUT	*Vitis* spp. interspecific rootstock	Leaf-feeding form
AUT281	48.68, 16.62	2015	AUT	*Vitis* spp. interspecific rootstock	Leaf-feeding form
AUT282	48.68, 16.62	2015	AUT	*Vitis* spp. interspecific rootstock	Leaf-feeding form
AUT283	48.68, 16.62	2015	AUT	*Vitis* spp. interspecific rootstock	Leaf-feeding form
AUT284	48.68, 16.62	2015	AUT	*Vitis* spp. interspecific rootstock	Leaf-feeding form
AUT285	48.68, 16.62	2015	AUT	*Vitis* spp. interspecific rootstock	Leaf-feeding form
AUT286	48.68, 16.62	2015	AUT	*Vitis* spp. interspecific rootstock	Leaf-feeding form
AUT287	48.68, 16.62	2015	AUT	*Vitis* spp. interspecific rootstock	Leaf-feeding form
AUT288	48.68, 16.62	2015	AUT	*Vitis* spp. interspecific rootstock	Leaf-feeding form
AUT289	48.68, 16.62	2015	AUT	*Vitis* spp. interspecific rootstock	Leaf-feeding form
AUT290	48.68, 16.62	2015	AUT	*Vitis* spp. interspecific rootstock	Leaf-feeding form
AUT291	48.68, 16.62	2015	AUT	*Vitis* spp. interspecific rootstock	Leaf-feeding form
AUT292	48.68, 16.62	2015	AUT	*Vitis* spp. interspecific rootstock	Leaf-feeding form
AUT293	48.68, 16.62	2015	AUT	*Vitis* spp. interspecific rootstock	Leaf-feeding form
AUT294	48.68, 16.62	2015	AUT	*Vitis* spp. interspecific rootstock	Leaf-feeding form
AUT295	48.68, 16.62	2015	AUT	*Vitis* spp. interspecific rootstock	Leaf-feeding form

Sample code	Sampling location (lat, long)	Sampling year	Country[1]	Host plant	Phylloxera feeding form
AUT296	48.68, 16.62	2015	AUT	*Vitis* spp. interspecific rootstock	Leaf-feeding form
AUT297	48.68, 16.62	2015	AUT	*Vitis* spp. interspecific rootstock	Leaf-feeding form
AUT298	48.68, 16.62	2015	AUT	*Vitis* spp. interspecific rootstock	Leaf-feeding form
AUT299	48.68, 16.62	2015	AUT	*Vitis* spp. interspecific rootstock	Leaf-feeding form
AUT300	48.68, 16.62	2015	AUT	*Vitis* spp. interspecific rootstock	Leaf-feeding form
AUT301	48.68, 16.62	2015	AUT	*Vitis* spp. interspecific rootstock	Leaf-feeding form
AUT302	48.68, 16.62	2015	AUT	*Vitis* spp. interspecific rootstock	Leaf-feeding form
AUT303	48.68, 16.62	2015	AUT	*Vitis* spp. interspecific rootstock	Leaf-feeding form
AUT304	48.68, 16.62	2015	AUT	*Vitis* spp. interspecific rootstock	Leaf-feeding form
AUT305	48.68, 16.62	2015	AUT	*Vitis* spp. interspecific rootstock	Leaf-feeding form
AUT306	48.68, 16.62	2015	AUT	*Vitis* spp. interspecific rootstock	Leaf-feeding form
AUT307	48.68, 16.62	2015	AUT	*Vitis* spp. interspecific rootstock	Leaf-feeding form
AUT308	48.68, 16.62	2015	AUT	*Vitis* spp. interspecific rootstock	Leaf-feeding form
AUT309	48.7, 16.71	2015	AUT	*Vitis* spp. interspecific rootstock	Leaf-feeding form
AUT310	48.7, 16.71	2015	AUT	*Vitis* spp. interspecific rootstock	Leaf-feeding form
AUT311	48.7, 16.71	2015	AUT	*Vitis* spp. interspecific rootstock	Leaf-feeding form
AUT312	48.7, 16.71	2015	AUT	*Vitis* spp. interspecific rootstock	Leaf-feeding form
AUT313	48.7, 16.71	2015	AUT	*Vitis* spp. interspecific rootstock	Leaf-feeding form
AUT314	48.7, 16.71	2015	AUT	*Vitis* spp. interspecific rootstock	Leaf-feeding form
AUT315	48.7, 16.71	2015	AUT	*Vitis* spp. interspecific rootstock	Leaf-feeding form
AUT316	48.7, 16.71	2015	AUT	*Vitis* spp. interspecific rootstock	Leaf-feeding form
AUT317	48.7, 16.71	2015	AUT	*Vitis* spp. interspecific rootstock	Leaf-feeding form
AUT318	48.7, 16.71	2015	AUT	*Vitis* spp. interspecific rootstock	Leaf-feeding form
AUT319	48.7, 16.71	2015	AUT	*Vitis* spp. interspecific rootstock	Leaf-feeding form
AUT320	48.7, 16.71	2015	AUT	*Vitis* spp. interspecific rootstock	Leaf-feeding form
AUT321	48.7, 16.71	2015	AUT	*Vitis* spp. interspecific rootstock	Leaf-feeding form
AUT322	48.7, 16.71	2015	AUT	*Vitis* spp. interspecific rootstock	Leaf-feeding form

Sample code	Sampling location (lat, long)	Sampling year	Country[1]	Host plant	Phylloxera feeding form
AUT323	48.7, 16.71	2015	AUT	*Vitis* spp. interspecific rootstock	Leaf-feeding form
AUT324	48.7, 16.71	2015	AUT	*Vitis* spp. interspecific rootstock	Leaf-feeding form
AUT325	48.7, 16.71	2015	AUT	*Vitis* spp. interspecific rootstock	Leaf-feeding form
AUT326	48.7, 16.71	2015	AUT	*Vitis* spp. interspecific rootstock	Leaf-feeding form
AUT327	48.7, 16.71	2015	AUT	*Vitis* spp. interspecific rootstock	Leaf-feeding form
AUT328	48.7, 16.71	2015	AUT	*Vitis* spp. interspecific rootstock	Leaf-feeding form
AUT329	48.7, 16.71	2015	AUT	*Vitis* spp. interspecific rootstock	Leaf-feeding form
AUT330	48.7, 16.71	2015	AUT	*Vitis* spp. interspecific rootstock	Leaf-feeding form
AUT331	48.7, 16.71	2015	AUT	*Vitis* spp. interspecific rootstock	Leaf-feeding form
AUT332	48.7, 16.71	2015	AUT	*Vitis* spp. interspecific rootstock	Leaf-feeding form
AUT333	48.7, 16.71	2015	AUT	*Vitis* spp. interspecific rootstock	Leaf-feeding form
AUT334	48.7, 16.71	2015	AUT	*Vitis* spp. interspecific rootstock	Leaf-feeding form
AUT335	48.7, 16.71	2015	AUT	*Vitis* spp. interspecific rootstock	Leaf-feeding form
AUT336	48.7, 16.71	2015	AUT	*Vitis* spp. interspecific rootstock	Leaf-feeding form
AUT337	48.7, 16.71	2015	AUT	*Vitis* spp. interspecific rootstock	Leaf-feeding form
AUT338	48.7, 16.71	2015	AUT	*Vitis* spp. interspecific rootstock	Leaf-feeding form
AUT339	48.7, 16.71	2015	AUT	*Vitis* spp. interspecific rootstock	Leaf-feeding form
AUT340	48.7, 16.71	2015	AUT	*Vitis* spp. interspecific rootstock	Leaf-feeding form
AUT341	48.7, 16.71	2015	AUT	*Vitis* spp. interspecific rootstock	Leaf-feeding form
AUT342	48.7, 16.71	2015	AUT	*Vitis* spp. interspecific rootstock	Leaf-feeding form
AUT343	48.7, 16.71	2015	AUT	*Vitis* spp. interspecific rootstock	Leaf-feeding form
AUT344	48.7, 16.71	2015	AUT	*Vitis* spp. interspecific rootstock	Leaf-feeding form
AUT345	48.7, 16.71	2015	AUT	*Vitis* spp. interspecific rootstock	Leaf-feeding form
AUT346	48.7, 16.71	2015	AUT	*Vitis* spp. interspecific rootstock	Leaf-feeding form
AUT347	48.64, 16.64	2015	AUT	*Vitis* spp. interspecific rootstock	Leaf-feeding form
AUT348	48.64, 16.64	2015	AUT	*Vitis* spp. interspecific rootstock	Leaf-feeding form
AUT349	48.64, 16.64	2015	AUT	*Vitis* spp. interspecific rootstock	Leaf-feeding form

Sample code	Sampling location (lat, long)	Sampling year	Country[1]	Host plant	Phylloxera feeding form
AUT350	48.64, 16.64	2015	AUT	*Vitis* spp. interspecific rootstock	Leaf-feeding form
AUT351	48.64, 16.64	2015	AUT	*Vitis* spp. interspecific rootstock	Leaf-feeding form
AUT352	48.64, 16.64	2015	AUT	*Vitis* spp. interspecific rootstock	Leaf-feeding form
AUT353	48.64, 16.64	2015	AUT	*Vitis* spp. interspecific rootstock	Leaf-feeding form
AUT354	48.64, 16.64	2015	AUT	*Vitis* spp. interspecific rootstock	Leaf-feeding form
AUT355	48.64, 16.64	2015	AUT	*Vitis* spp. interspecific rootstock	Leaf-feeding form
AUT356	48.64, 16.64	2015	AUT	*Vitis* spp. interspecific rootstock	Leaf-feeding form
AUT357	48.64, 16.64	2015	AUT	*Vitis* spp. interspecific rootstock	Leaf-feeding form
AUT358	48.64, 16.64	2015	AUT	*Vitis* spp. interspecific rootstock	Leaf-feeding form
AUT359	48.64, 16.64	2015	AUT	*Vitis* spp. interspecific rootstock	Leaf-feeding form
AUT360	48.64, 16.64	2015	AUT	*Vitis* spp. interspecific rootstock	Leaf-feeding form
AUT361	48.64, 16.64	2015	AUT	*Vitis* spp. interspecific rootstock	Leaf-feeding form
AUT362	48.64, 16.64	2015	AUT	*Vitis* spp. interspecific rootstock	Leaf-feeding form
AUT363	48.64, 16.64	2015	AUT	*Vitis* spp. interspecific rootstock	Leaf-feeding form
AUT364	48.64, 16.64	2015	AUT	*Vitis* spp. interspecific rootstock	Leaf-feeding form
AUT365	48.64, 16.64	2015	AUT	*Vitis* spp. interspecific rootstock	Leaf-feeding form
AUT366	48.64, 16.64	2015	AUT	*Vitis* spp. interspecific rootstock	Leaf-feeding form
AUT367	48.64, 16.64	2015	AUT	*Vitis* spp. interspecific rootstock	Leaf-feeding form
AUT368	48.64, 16.64	2015	AUT	*Vitis* spp. interspecific rootstock	Leaf-feeding form
AUT369	48.63, 16.65	2015	AUT	*Vitis* spp. interspecific rootstock	Leaf-feeding form
AUT370	48.63, 16.65	2015	AUT	*Vitis* spp. interspecific rootstock	Leaf-feeding form
AUT371	48.63, 16.65	2015	AUT	*Vitis* spp. interspecific rootstock	Leaf-feeding form
AUT372	48.63, 16.65	2015	AUT	*Vitis* spp. interspecific rootstock	Leaf-feeding form
AUT373	48.63, 16.65	2015	AUT	*Vitis* spp. interspecific rootstock	Leaf-feeding form
AUT374	48.63, 16.65	2015	AUT	*Vitis* spp. interspecific rootstock	Leaf-feeding form
AUT375	48.63, 16.65	2015	AUT	*Vitis* spp. interspecific rootstock	Leaf-feeding form
AUT376	48.63, 16.65	2015	AUT	*Vitis* spp. interspecific rootstock	Leaf-feeding form

Sample code	Sampling location (lat, long)	Sampling year	Country[1]	Host plant	Phylloxera feeding form
AUT377	48.63, 16.65	2015	AUT	*Vitis* spp. interspecific rootstock	Leaf-feeding form
AUT378	48.63, 16.65	2015	AUT	*Vitis* spp. interspecific rootstock	Leaf-feeding form
AUT379	48.63, 16.65	2015	AUT	*Vitis* spp. interspecific rootstock	Leaf-feeding form
AUT380	48.63, 16.65	2015	AUT	*Vitis* spp. interspecific rootstock	Leaf-feeding form
AUT381	48.63, 16.65	2015	AUT	*Vitis* spp. interspecific rootstock	Leaf-feeding form
AUT382	48.63, 16.65	2015	AUT	*Vitis* spp. interspecific rootstock	Leaf-feeding form
AUT383	48.63, 16.65	2015	AUT	*Vitis* spp. interspecific rootstock	Leaf-feeding form
AUT384	48.63, 16.65	2015	AUT	*Vitis* spp. interspecific rootstock	Leaf-feeding form
AUT385	48.63, 16.65	2015	AUT	*Vitis* spp. interspecific rootstock	Leaf-feeding form
AUT386	48.63, 16.65	2015	AUT	*Vitis* spp. interspecific rootstock	Leaf-feeding form
AUT387	48.63, 16.65	2015	AUT	*Vitis* spp. interspecific rootstock	Leaf-feeding form
AUT388	48.63, 16.65	2015	AUT	*Vitis* spp. interspecific rootstock	Leaf-feeding form
AUT389	48.63, 16.65	2015	AUT	*Vitis* spp. interspecific rootstock	Leaf-feeding form
AUT390	48.63, 16.65	2015	AUT	*Vitis* spp. interspecific rootstock	Leaf-feeding form
AUT391	48.63, 16.65	2015	AUT	*Vitis* spp. interspecific rootstock	Leaf-feeding form
DEU001	50.03, 8.12	2018	DEU	*Vitis vinifera* L. cultivar	Leaf-feeding form
DEU002	50.03, 8.12	2018	DEU	*Vitis vinifera* L. cultivar	Leaf-feeding form
DEU003	50.03, 8.12	2018	DEU	*Vitis vinifera* L. cultivar	Leaf-feeding form
DEU004	50.03, 8.12	2018	DEU	*Vitis vinifera* L. cultivar	Leaf-feeding form
DEU005	50.03, 8.12	2018	DEU	*Vitis vinifera* L. cultivar	Leaf-feeding form
DEU006	n.a.	2018	DEU	*Vitis vinifera* L. cultivar	Leaf-feeding form
DEU007	n.a.	2018	DEU	*Vitis vinifera* L. cultivar	Leaf-feeding form
DEU008	n.a.	2018	DEU	*Vitis vinifera* L. cultivar	Leaf-feeding form
DEU009	n.a.	2018	DEU	*Vitis vinifera* L. cultivar	Leaf-feeding form
DEU010	n.a.	2018	DEU	*Vitis vinifera* L. cultivar	Leaf-feeding form
DEU011	n.a.	2018	DEU	*Vitis vinifera* L. cultivar	Leaf-feeding form
DEU012	50.03, 8.12	2018	DEU	*Vitis vinifera* L. cultivar	Leaf-feeding form

Sample code	Sampling location (lat, long)	Sampling year	Country[1]	Host plant	Phylloxera feeding form
DEU013	50.03, 8.12	2018	DEU	*Vitis vinifera* L. cultivar	Leaf-feeding form
DEU014	50.03, 8.12	2018	DEU	*Vitis vinifera* L. cultivar	Leaf-feeding form
DEU015	50.03, 8.12	2018	DEU	*Vitis vinifera* L. cultivar	Leaf-feeding form
DEU016	50.03, 8.12	2018	DEU	*Vitis vinifera* L. cultivar	Leaf-feeding form
DEU017	50.01, 8.02	2018	DEU	*Vitis vinifera* L. cultivar	Leaf-feeding form
DEU018	50.01, 8.02	2018	DEU	*Vitis vinifera* L. cultivar	Leaf-feeding form
DEU019	47.95, 7.75	2015	DEU	*Vitis vinifera* L. cultivar	Leaf-feeding form
DEU020	47.95, 7.75	2015	DEU	*Vitis vinifera* L. cultivar	Leaf-feeding form
DEU021	47.95, 7.75	2015	DEU	*Vitis vinifera* L. cultivar	Leaf-feeding form
DEU022	47.95, 7.75	2015	DEU	*Vitis vinifera* L. cultivar	Leaf-feeding form
DEU023	47.95, 7.75	2015	DEU	*Vitis vinifera* L. cultivar	Leaf-feeding form
DEU024	47.95, 7.75	2015	DEU	*Vitis vinifera* L. cultivar	Leaf-feeding form
DEU025	47.95, 7.75	2015	DEU	*Vitis vinifera* L. cultivar	Leaf-feeding form
DEU026	47.95, 7.75	2015	DEU	*Vitis vinifera* L. cultivar	Leaf-feeding form
DEU027	47.95, 7.75	2015	DEU	*Vitis vinifera* L. cultivar	Leaf-feeding form
DEU028	47.95, 7.75	2015	DEU	*Vitis vinifera* L. cultivar	Leaf-feeding form
DEU029	47.95, 7.75	2015	DEU	*Vitis vinifera* L. cultivar	Leaf-feeding form
DEU030	47.96, 7.77	2015	DEU	*Vitis* spp. interspecific hybrid direct-producer	Leaf-feeding form
DEU031	47.96, 7.77	2015	DEU	*Vitis* spp. interspecific hybrid direct-producer	Leaf-feeding form
DEU032	47.96, 7.77	2015	DEU	*Vitis* spp. interspecific hybrid direct-producer	Leaf-feeding form
DEU033	47.96, 7.77	2015	DEU	*Vitis* spp. interspecific hybrid direct-producer	Leaf-feeding form
DEU034	47.96, 7.77	2015	DEU	*Vitis* spp. interspecific hybrid direct-producer	Leaf-feeding form
DEU035	47.96, 7.77	2015	DEU	*Vitis* spp. interspecific hybrid direct-producer	Leaf-feeding form
DEU036	47.96, 7.77	2015	DEU	*Vitis* spp. interspecific hybrid direct-producer	Leaf-feeding form
DEU037	47.96, 7.77	2015	DEU	*Vitis* spp. interspecific hybrid direct-producer	Leaf-feeding form
DEU038	47.96, 7.77	2015	DEU	*Vitis* spp. interspecific hybrid direct-producer	Leaf-feeding form
DEU039	47.96, 7.77	2015	DEU	*Vitis* spp. interspecific hybrid direct-producer	Leaf-feeding form

Sample code	Sampling location (lat, long)	Sampling year	Country[1]	Host plant	Phylloxera feeding form
DEU040	47.96, 7.77	2015	DEU	*Vitis* spp. interspecific hybrid direct-producer	Leaf-feeding form
DEU041	47.96, 7.77	2015	DEU	*Vitis* spp. interspecific hybrid direct-producer	Leaf-feeding form
DEU042	47.96, 7.77	2015	DEU	*Vitis* spp. interspecific hybrid direct-producer	Leaf-feeding form
DEU043	47.96, 7.77	2015	DEU	*Vitis* spp. interspecific hybrid direct-producer	Leaf-feeding form
DEU044	47.96, 7.77	2015	DEU	*Vitis* spp. interspecific hybrid direct-producer	Leaf-feeding form
DEU045	47.96, 7.77	2015	DEU	*Vitis* spp. interspecific hybrid direct-producer	Leaf-feeding form
DEU046	47.96, 7.77	2015	DEU	*Vitis* spp. interspecific hybrid direct-producer	Leaf-feeding form
DEU047	47.96, 7.77	2015	DEU	*Vitis* spp. interspecific hybrid direct-producer	Leaf-feeding form
DEU048	47.96, 7.77	2015	DEU	*Vitis* spp. interspecific hybrid direct-producer	Leaf-feeding form
DEU049	47.96, 7.77	2015	DEU	*Vitis* spp. interspecific hybrid direct-producer	Leaf-feeding form
DEU050	47.96, 7.77	2015	DEU	*Vitis* spp. interspecific hybrid direct-producer	Leaf-feeding form
DEU051	47.96, 7.77	2015	DEU	*Vitis* spp. interspecific hybrid direct-producer	Leaf-feeding form
DEU052	47.96, 7.77	2015	DEU	*Vitis* spp. interspecific hybrid direct-producer	Leaf-feeding form
DEU053	47.96, 7.77	2015	DEU	*Vitis* spp. interspecific hybrid direct-producer	Leaf-feeding form
DEU054	47.96, 7.77	2015	DEU	*Vitis* spp. interspecific hybrid direct-producer	Leaf-feeding form
DEU055	47.96, 7.77	2015	DEU	*Vitis* spp. interspecific hybrid direct-producer	Leaf-feeding form
DEU056	47.96, 7.77	2015	DEU	*Vitis* spp. interspecific hybrid direct-producer	Leaf-feeding form
DEU057	48.19, 7.81	2015	DEU	*Vitis* spp. interspecific hybrid direct-producer	Leaf-feeding form
DEU058	48.19, 7.81	2015	DEU	*Vitis* spp. interspecific hybrid direct-producer	Leaf-feeding form
DEU059	48.19, 7.81	2015	DEU	*Vitis* spp. interspecific hybrid direct-producer	Leaf-feeding form
DEU060	48.19, 7.81	2015	DEU	*Vitis* spp. interspecific hybrid direct-producer	Leaf-feeding form
DEU061	48.19, 7.81	2015	DEU	*Vitis* spp. interspecific hybrid direct-producer	Leaf-feeding form
DEU062	48.19, 7.81	2015	DEU	*Vitis* spp. interspecific hybrid direct-producer	Leaf-feeding form
DEU063	48.19, 7.81	2015	DEU	*Vitis* spp. interspecific hybrid direct-producer	Leaf-feeding form
DEU064	48.19, 7.81	2015	DEU	*Vitis* spp. interspecific hybrid direct-producer	Leaf-feeding form
DEU065	48.19, 7.81	2015	DEU	*Vitis* spp. interspecific hybrid direct-producer	Leaf-feeding form
DEU066	48.19, 7.81	2015	DEU	*Vitis* spp. interspecific hybrid direct-producer	Leaf-feeding form

Sample code	Sampling location (lat, long)	Sampling year	Country[1]	Host plant	Phylloxera feeding form
DEU067	48.19, 7.81	2015	DEU	*Vitis* spp. interspecific hybrid direct-producer	Leaf-feeding form
DEU068	48.19, 7.81	2015	DEU	*Vitis* spp. interspecific hybrid direct-producer	Leaf-feeding form
DEU069	48.19, 7.81	2015	DEU	*Vitis* spp. interspecific hybrid direct-producer	Leaf-feeding form
DEU070	48.19, 7.81	2015	DEU	*Vitis* spp. interspecific hybrid direct-producer	Leaf-feeding form
DEU071	48.19, 7.81	2015	DEU	*Vitis* spp. interspecific hybrid direct-producer	Leaf-feeding form
DEU072	48.19, 7.81	2015	DEU	*Vitis* spp. interspecific hybrid direct-producer	Leaf-feeding form
DEU073	48.19, 7.81	2015	DEU	*Vitis* spp. interspecific hybrid direct-producer	Leaf-feeding form
DEU074	48.19, 7.81	2015	DEU	*Vitis* spp. interspecific hybrid direct-producer	Leaf-feeding form
DEU075	48.19, 7.81	2015	DEU	*Vitis* spp. interspecific hybrid direct-producer	Leaf-feeding form
DEU076	48.19, 7.81	2015	DEU	*Vitis* spp. interspecific hybrid direct-producer	Leaf-feeding form
DEU077	48.19, 7.81	2015	DEU	*Vitis* spp. interspecific hybrid direct-producer	Leaf-feeding form
DEU078	48.19, 7.81	2015	DEU	*Vitis* spp. interspecific hybrid direct-producer	Leaf-feeding form
DEU079	48.19, 7.81	2015	DEU	*Vitis* spp. interspecific hybrid direct-producer	Leaf-feeding form
DEU080	48.19, 7.81	2015	DEU	*Vitis* spp. interspecific hybrid direct-producer	Leaf-feeding form
DEU081	48.19, 7.81	2015	DEU	*Vitis* spp. interspecific hybrid direct-producer	Leaf-feeding form
DEU082	48.19, 7.81	2015	DEU	*Vitis* spp. interspecific hybrid direct-producer	Leaf-feeding form
DEU083	48.19, 7.81	2015	DEU	*Vitis* spp. interspecific hybrid direct-producer	Leaf-feeding form
DEU084	48.19, 7.81	2015	DEU	*Vitis* spp. interspecific hybrid direct-producer	Leaf-feeding form
DEU085	48.19, 7.81	2015	DEU	*Vitis* spp. interspecific hybrid direct-producer	Leaf-feeding form
DEU086	48.19, 7.81	2015	DEU	*Vitis* spp. interspecific hybrid direct-producer	Leaf-feeding form
DEU087	48.19, 7.81	2015	DEU	*Vitis* spp. interspecific hybrid direct-producer	Leaf-feeding form
DEU088	48.19, 7.81	2015	DEU	*Vitis* spp. interspecific hybrid direct-producer	Leaf-feeding form
DEU089	48.19, 7.81	2015	DEU	*Vitis* spp. interspecific hybrid direct-producer	Leaf-feeding form
DEU090	48.19, 7.81	2015	DEU	*Vitis* spp. interspecific hybrid direct-producer	Leaf-feeding form
DEU091	48.19, 7.81	2015	DEU	*Vitis* spp. interspecific hybrid direct-producer	Leaf-feeding form
DEU092	48.19, 7.81	2015	DEU	*Vitis* spp. interspecific hybrid direct-producer	Leaf-feeding form
DEU093	48.19, 7.81	2015	DEU	*Vitis* spp. interspecific hybrid direct-producer	Leaf-feeding form

**Sample code**	**Sampling location (lat, long)**	**Sampling year**	**Country[1]**	**Host plant**	**Phylloxera feeding form**
DEU094	47.83, 7.67	n.a.	DEU	*Vitis vinifera* L. cultivar	Leaf-feeding form
DEU095	47.83, 7.67	n.a.	DEU	*Vitis vinifera* L. cultivar	Leaf-feeding form
DEU096	47.83, 7.67	n.a.	DEU	*Vitis vinifera* L. cultivar	Leaf-feeding form
DEU097	47.83, 7.67	n.a.	DEU	*Vitis vinifera* L. cultivar	Root-feeding form
DEU098	47.83, 7.67	n.a.	DEU	*Vitis vinifera* L. cultivar	Root-feeding form
DEU099	47.94, 7.76	n.a.	DEU	*Vitis vinifera* L. cultivar	Leaf-feeding form
DEU100	47.94, 7.76	n.a.	DEU	*Vitis vinifera* L. cultivar	Leaf-feeding form
DEU101	47.94, 7.76	n.a.	DEU	*Vitis vinifera* L. cultivar	Leaf-feeding form
DEU102	47.94, 7.76	n.a.	DEU	*Vitis vinifera* L. cultivar	Root-feeding form
DEU103	47.94, 7.76	n.a.	DEU	*Vitis vinifera* L. cultivar	Root-feeding form
DEU104	48.04, 7.65	n.a.	DEU	*Vitis* spp. interspecific hybrid direct-producer	Leaf-feeding form
DEU105	48.04, 7.65	n.a.	DEU	*Vitis* spp. interspecific hybrid direct-producer	Leaf-feeding form
DEU106	48.04, 7.65	n.a.	DEU	*Vitis* spp. interspecific hybrid direct-producer	Leaf-feeding form
DEU107	48.04, 7.65	n.a.	DEU	*Vitis* spp. interspecific hybrid direct-producer	Leaf-feeding form
DEU108	48.04, 7.65	n.a.	DEU	*Vitis vinifera* L. cultivar	Root-feeding form
DEU109	48.12, 7.74	n.a.	DEU	*Vitis* spp. interspecific hybrid direct-producer	Leaf-feeding form
DEU110	48.12, 7.74	n.a.	DEU	*Vitis* spp. interspecific hybrid direct-producer	Leaf-feeding form
DEU111	48.12, 7.74	n.a.	DEU	*Vitis* spp. interspecific hybrid direct-producer	Leaf-feeding form
DEU112	48.12, 7.74	n.a.	DEU	*Vitis* spp. interspecific hybrid direct-producer	Leaf-feeding form
DEU113	48.12, 7.74	n.a.	DEU	*Vitis* spp. interspecific hybrid direct-producer	Root-feeding form
DEU114	n.a.	n.a.	DEU	*Vitis vinifera* L. cultivar	Leaf-feeding form
DEU115	n.a.	n.a.	DEU	*Vitis vinifera* L. cultivar	Leaf-feeding form
DEU116	n.a.	n.a.	DEU	*Vitis vinifera* L. cultivar	Leaf-feeding form
DEU117	n.a.	n.a.	DEU	*Vitis vinifera* L. cultivar	Leaf-feeding form
CHE001	n.a.	2018	CHE	*Vitis* spp. interspecific hybrid direct-producer	Leaf-feeding form
CHE002	n.a.	2018	CHE	*Vitis* spp. interspecific hybrid direct-producer	Leaf-feeding form
CHE003	n.a.	2018	CHE	*Vitis* spp. interspecific hybrid direct-producer	Leaf-feeding form

Sample code	Sampling location (lat, long)	Sampling year	Country[1]	Host plant	Phylloxera feeding form
CHE004	46.42, 6.27	2018	CHE	*Vitis vinifera* L. cultivar	Leaf-feeding form
CHE005	46.42, 6.27	n.a.	CHE	*Vitis vinifera* L. cultivar	Leaf-feeding form
CHE006	47.57, 8.25	2013	CHE	*Vitis* spp. interspecific rootstock	Leaf-feeding form
CHE007	47.57, 8.25	2013	CHE	*Vitis* spp. interspecific rootstock	Leaf-feeding form
CHE008	47.57, 8.25	2013	CHE	*Vitis* spp. interspecific rootstock	Leaf-feeding form
CHE009	47.57, 8.25	2013	CHE	*Vitis* spp. interspecific rootstock	Leaf-feeding form
CHE010	47.42, 8.17	2013	CHE	*Vitis* spp. interspecific hybrid direct-producer	Leaf-feeding form
CHE011	47.42, 8.17	2013	CHE	*Vitis* spp. interspecific hybrid direct-producer	Leaf-feeding form
CHE012	47.42, 8.17	2013	CHE	*Vitis* spp. interspecific hybrid direct-producer	Leaf-feeding form
CHE013	47.42, 8.17	2013	CHE	*Vitis* spp. interspecific hybrid direct-producer	Leaf-feeding form
CHE014	47.42, 8.17	2013	CHE	*Vitis* spp. interspecific hybrid direct-producer	Leaf-feeding form
CHE015	47.42, 8.17	2013	CHE	*Vitis* spp. interspecific hybrid direct-producer	Root-feeding form
CHE016	47.42, 8.17	2013	CHE	*Vitis* spp. interspecific hybrid direct-producer	Root-feeding form
CHE017	47.42, 8.17	2013	CHE	*Vitis* spp. interspecific hybrid direct-producer	Root-feeding form
CHE018	47.42, 8.17	2013	CHE	*Vitis* spp. interspecific hybrid direct-producer	Root-feeding form
CHE019	47.43, 8.13	2013	CHE	*Vitis* spp. interspecific hybrid direct-producer	Leaf-feeding form
CHE020	47.43, 8.13	2013	CHE	*Vitis* spp. interspecific hybrid direct-producer	Leaf-feeding form
CHE021	47.43, 8.13	2013	CHE	*Vitis* spp. interspecific hybrid direct-producer	Leaf-feeding form
CHE022	47.43, 8.13	2013	CHE	*Vitis* spp. interspecific hybrid direct-producer	Leaf-feeding form
CHE023	47.43, 8.13	2013	CHE	*Vitis* spp. interspecific hybrid direct-producer	Leaf-feeding form
CHE024	47.43, 8.13	2013	CHE	*Vitis* spp. interspecific hybrid direct-producer	Root-feeding form
CHE025	47.43, 8.13	2013	CHE	*Vitis* spp. interspecific hybrid direct-producer	Root-feeding form
CHE026	47.43, 8.13	2013	CHE	*Vitis* spp. interspecific hybrid direct-producer	Root-feeding form
CHE027	47.43, 8.13	2013	CHE	*Vitis* spp. interspecific hybrid direct-producer	Root-feeding form
CHE028	47.43, 8.13	2013	CHE	*Vitis* spp. interspecific hybrid direct-producer	Root-feeding form
CHE029	47.43, 8.13	2013	CHE	*Vitis* spp. interspecific hybrid direct-producer	Root-feeding form
CHE030	47.43, 8.12	2013	CHE	*Vitis* spp. interspecific hybrid direct-producer	Leaf-feeding form

**Sample code**	**Sampling location (lat, long)**	**Sampling year**	**Country**[1]	**Host plant**	**Phylloxera feeding form**
CHE031	47.43, 8.12	2013	CHE	*Vitis* spp. interspecific hybrid direct-producer	Leaf-feeding form
CHE032	47.43, 8.12	2013	CHE	*Vitis* spp. interspecific hybrid direct-producer	Leaf-feeding form
CHE033	47.43, 8.12	2013	CHE	*Vitis* spp. interspecific hybrid direct-producer	Leaf-feeding form
CHE034	47.43, 8.12	2013	CHE	*Vitis* spp. interspecific hybrid direct-producer	Leaf-feeding form
CHE035	47.43, 8.12	2013	CHE	*Vitis* spp. interspecific hybrid direct-producer	Root-feeding form
CHE036	47.43, 8.12	2013	CHE	*Vitis* spp. interspecific hybrid direct-producer	Root-feeding form
CHE037	47.43, 8.12	2013	CHE	*Vitis* spp. interspecific hybrid direct-producer	Root-feeding form
CHE038	47.43, 8.12	2013	CHE	*Vitis* spp. interspecific hybrid direct-producer	Root-feeding form
CHE039	47.43, 8.12	2013	CHE	*Vitis* spp. interspecific hybrid direct-producer	Root-feeding form
CHE040	47.43, 8.12	2013	CHE	*Vitis* spp. interspecific hybrid direct-producer	Root-feeding form
CHE041	47.43, 8.12	2013	CHE	*Vitis* spp. interspecific hybrid direct-producer	Root-feeding form
CHE042	47.43, 8.12	2013	CHE	*Vitis* spp. interspecific hybrid direct-producer	Root-feeding form
CHE043	47.43, 8.12	2013	CHE	*Vitis* spp. interspecific hybrid direct-producer	Root-feeding form
CHE044	47.43, 8.12	2013	CHE	*Vitis* spp. interspecific hybrid direct-producer	Root-feeding form
CHE045	47.43, 8.12	2013	CHE	*Vitis* spp. interspecific hybrid direct-producer	Root-feeding form
CHE046	47.43, 8.12	2013	CHE	*Vitis* spp. interspecific hybrid direct-producer	Root-feeding form
CHE047	47.43, 8.12	2013	CHE	*Vitis* spp. interspecific hybrid direct-producer	Root-feeding form
CHE048	47.43, 8.12	2013	CHE	*Vitis* spp. interspecific hybrid direct-producer	Root-feeding form
CHE049	47.43, 8.12	2013	CHE	*Vitis* spp. interspecific hybrid direct-producer	Root-feeding form
CHE050	47.43, 8.12	2013	CHE	*Vitis* spp. interspecific hybrid direct-producer	Root-feeding form
CHE051	47.43, 8.12	2013	CHE	*Vitis* spp. interspecific hybrid direct-producer	Root-feeding form
CHE052	47.43, 8.12	2013	CHE	*Vitis* spp. interspecific hybrid direct-producer	Root-feeding form
CHE053	47.52, 7.83	2013	CHE	*Vitis* spp. interspecific resistant grape variety	Leaf-feeding form
CHE054	47.52, 7.83	2013	CHE	*Vitis* spp. interspecific resistant grape variety	Leaf-feeding form
CHE055	47.52, 7.83	2013	CHE	*Vitis* spp. interspecific resistant grape variety	Leaf-feeding form
CHE056	47.52, 7.83	2013	CHE	*Vitis* spp. interspecific resistant grape variety	Leaf-feeding form
CHE057	47.52, 7.83	2013	CHE	*Vitis* spp. interspecific resistant grape variety	Leaf-feeding form

Sample code	Sampling location (lat, long)	Sampling year	Country[1]	Host plant	Phylloxera feeding form
CHE058	47.52, 7.83	2013	CHE	*Vitis* spp. interspecific resistant grape variety	Root-feeding form
CHE059	47.52, 7.83	2013	CHE	*Vitis* spp. interspecific resistant grape variety	Root-feeding form
CHE060	47.52, 7.82	2013	CHE	*Vitis* spp. interspecific resistant grape variety	Root-feeding form
CHE061	47.52, 7.82	2013	CHE	*Vitis* spp. interspecific resistant grape variety	Root-feeding form
CHE062	47.52, 7.82	2013	CHE	*Vitis* spp. interspecific resistant grape variety	Root-feeding form
CHE063	47.52, 7.82	2013	CHE	*Vitis* spp. interspecific resistant grape variety	Leaf-feeding form
CHE064	47.52, 7.82	2013	CHE	*Vitis* spp. interspecific resistant grape variety	Leaf-feeding form
CHE065	47.52, 7.82	2013	CHE	*Vitis* spp. interspecific resistant grape variety	Leaf-feeding form
CHE066	47.52, 7.82	2013	CHE	*Vitis* spp. interspecific resistant grape variety	Leaf-feeding form
CHE067	47.52, 7.82	2013	CHE	*Vitis* spp. interspecific resistant grape variety	Leaf-feeding form
CHE068	47.52, 7.82	2013	CHE	*Vitis vinifera* L. cultivar	Root-feeding form
CHE069	47.52, 7.82	2013	CHE	*Vitis vinifera* L. cultivar	Root-feeding form
CHE070	47.52, 7.82	2013	CHE	*Vitis vinifera* L. cultivar	Leaf-feeding form
CHE071	47.52, 7.82	2013	CHE	*Vitis vinifera* L. cultivar	Leaf-feeding form
CHE072	47.52, 7.82	2013	CHE	*Vitis vinifera* L. cultivar	Leaf-feeding form
CHE073	47.52, 7.82	2013	CHE	*Vitis vinifera* L. cultivar	Leaf-feeding form
CHE074	47.52, 7.82	2013	CHE	*Vitis vinifera* L. cultivar	Leaf-feeding form
CHE075	47.48, 7.57	2013	CHE	*Vitis* spp. interspecific hybrid direct-producer	Leaf-feeding form
CHE076	47.48, 7.57	2013	CHE	*Vitis* spp. interspecific hybrid direct-producer	Leaf-feeding form
CHE077	47.48, 7.57	2013	CHE	*Vitis* spp. interspecific hybrid direct-producer	Leaf-feeding form
CHE078	47.48, 7.57	2013	CHE	*Vitis* spp. interspecific hybrid direct-producer	Leaf-feeding form
CHE079	47.48, 7.57	2013	CHE	*Vitis* spp. interspecific hybrid direct-producer	Leaf-feeding form
CHE080	47.48, 7.57	2013	CHE	*Vitis* spp. interspecific hybrid direct-producer	Root-feeding form
CHE081	47.48, 7.48	2013	CHE	*Vitis vinifera* L. cultivar	Leaf-feeding form
CHE082	47.48, 7.48	2013	CHE	*Vitis vinifera* L. cultivar	Leaf-feeding form
CHE083	47.48, 7.48	2013	CHE	*Vitis vinifera* L. cultivar	Leaf-feeding form
CHE084	47.48, 7.48	2013	CHE	*Vitis vinifera* L. cultivar	Leaf-feeding form

**Sample code**	**Sampling location (lat, long)**	**Sampling year**	**Country[1]**	**Host plant**	**Phylloxera feeding form**
CHE085	47.48, 7.48	2013	CHE	*Vitis vinifera* L. cultivar	Leaf-feeding form
CHE086	47.48, 7.48	2013	CHE	*Vitis* spp. interspecific rootstock	Root-feeding form
CHE087	47.48, 7.48	2013	CHE	*Vitis* spp. interspecific rootstock	Root-feeding form
CHE088	47.5, 7.53	2013	CHE	*Vitis* spp. interspecific hybrid direct-producer	Leaf-feeding form
CHE089	47.5, 7.53	2013	CHE	*Vitis* spp. interspecific hybrid direct-producer	Leaf-feeding form
CHE090	47.5, 7.53	2013	CHE	*Vitis* spp. interspecific hybrid direct-producer	Leaf-feeding form
CHE091	47.5, 7.53	2013	CHE	*Vitis* spp. interspecific hybrid direct-producer	Leaf-feeding form
CHE092	47.5, 7.53	2013	CHE	*Vitis* spp. interspecific hybrid direct-producer	Leaf-feeding form
CHE093	47.5, 7.53	2013	CHE	*Vitis* spp. interspecific hybrid direct-producer	Leaf-feeding form
CHE094	47.5, 7.53	2013	CHE	*Vitis* spp. interspecific hybrid direct-producer	Leaf-feeding form
CHE095	47.5, 7.53	2013	CHE	*Vitis* spp. interspecific hybrid direct-producer	Leaf-feeding form
CHE096	47.5, 7.53	2013	CHE	*Vitis* spp. interspecific hybrid direct-producer	Leaf-feeding form
CHE097	47.5, 7.53	2013	CHE	n.a.	n.a.
CHE098	46.98, 9.55	2013	CHE	*Vitis* spp. interspecific hybrid direct-producer	Leaf-feeding form
CHE099	46.98, 9.55	2013	CHE	*Vitis* spp. interspecific hybrid direct-producer	Leaf-feeding form
CHE100	46.98, 9.55	2013	CHE	*Vitis* spp. interspecific hybrid direct-producer	Leaf-feeding form
CHE101	46.98, 9.55	2013	CHE	*Vitis* spp. interspecific hybrid direct-producer	Leaf-feeding form
CHE102	46.98, 9.55	2013	CHE	*Vitis* spp. interspecific hybrid direct-producer	Leaf-feeding form
CHE103	46.98, 9.55	2013	CHE	*Vitis* spp. interspecific hybrid direct-producer	Root-feeding form
CHE104	46.98, 9.55	2013	CHE	*Vitis* spp. interspecific hybrid direct-producer	Root-feeding form
CHE105	46.98, 9.55	2013	CHE	*Vitis* spp. interspecific hybrid direct-producer	Root-feeding form
CHE106	46.98, 9.55	2013	CHE	*Vitis* spp. interspecific hybrid direct-producer	Root-feeding form
CHE107	46.98, 9.55	2013	CHE	*Vitis* spp. interspecific hybrid direct-producer	Root-feeding form
CHE108	46.98, 9.55	2013	CHE	*Vitis* spp. interspecific hybrid direct-producer	Root-feeding form
CHE109	46.98, 9.55	2013	CHE	*Vitis* spp. interspecific hybrid direct-producer	Root-feeding form
CHE110	46.98, 9.55	2013	CHE	*Vitis* spp. interspecific hybrid direct-producer	Root-feeding form
CHE111	46.98, 9.55	2013	CHE	*Vitis* spp. interspecific hybrid direct-producer	Root-feeding form

**Sample code**	**Sampling location (lat, long)**	**Sampling year**	**Country**[1]	**Host plant**	**Phylloxera feeding form**
CHE112	46.97, 9.57	2013	CHE	*Vitis* spp. interspecific rootstock	Root-feeding form
CHE113	46.97, 9.57	2013	CHE	*Vitis* spp. interspecific rootstock	Root-feeding form
CHE114	46.97, 9.57	2013	CHE	*Vitis* spp. interspecific rootstock	Root-feeding form
CHE115	46.97, 9.57	2013	CHE	*Vitis* spp. interspecific rootstock	Root-feeding form
CHE116	46.97, 9.57	2013	CHE	*Vitis* spp. interspecific rootstock	Root-feeding form
CHE117	47.05, 9.43	2013	CHE	*Vitis* spp. interspecific hybrid direct-producer	Leaf-feeding form
CHE118	47.05, 9.43	2013	CHE	*Vitis* spp. interspecific hybrid direct-producer	Leaf-feeding form
CHE119	47.05, 9.43	2013	CHE	*Vitis* spp. interspecific hybrid direct-producer	Leaf-feeding form
CHE120	47.05, 9.43	2013	CHE	*Vitis* spp. interspecific hybrid direct-producer	Leaf-feeding form
CHE121	47.05, 9.43	2013	CHE	*Vitis* spp. interspecific hybrid direct-producer	Leaf-feeding form
CHE122	47.05, 9.43	2013	CHE	*Vitis* spp. interspecific rootstock	Root-feeding form
CHE123	47.05, 9.43	2013	CHE	*Vitis* spp. interspecific rootstock	Root-feeding form
CHE124	47.05, 9.43	2013	CHE	*Vitis* spp. interspecific rootstock	Root-feeding form
CHE125	47.05, 9.43	2013	CHE	*Vitis* spp. interspecific rootstock	Root-feeding form
CHE126	47.05, 9.43	2013	CHE	*Vitis* spp. interspecific rootstock	Root-feeding form
CHE127	47.12, 9.23	n.a.	CHE	*Vitis* spp. interspecific hybrid direct-producer	Leaf-feeding form
CHE128	47.12, 9.23	n.a.	CHE	*Vitis* spp. interspecific hybrid direct-producer	Leaf-feeding form
CHE129	47.12, 9.23	n.a.	CHE	*Vitis* spp. interspecific hybrid direct-producer	Leaf-feeding form
CHE130	47.12, 9.23	n.a.	CHE	*Vitis* spp. interspecific hybrid direct-producer	Leaf-feeding form
CHE131	47.12, 9.23	n.a.	CHE	*Vitis* spp. interspecific hybrid direct-producer	Leaf-feeding form
CHE132	47.12, 9.23	n.a.	CHE	*Vitis* spp. interspecific hybrid direct-producer	Leaf-feeding form
CHE133	47.12, 9.23	n.a.	CHE	*Vitis* spp. interspecific hybrid direct-producer	Leaf-feeding form
CHE134	47.12, 9.23	n.a.	CHE	*Vitis* spp. interspecific hybrid direct-producer	Leaf-feeding form
CHE135	47.12, 9.23	n.a.	CHE	*Vitis* spp. interspecific hybrid direct-producer	Leaf-feeding form
CHE136	47.83, 8.97	2013	CHE	*Vitis vinifera* L. cultivar	Leaf-feeding form
CHE137	47.83, 8.97	2013	CHE	*Vitis vinifera* L. cultivar	Leaf-feeding form
CHE138	47.83, 8.97	2013	CHE	*Vitis vinifera* L. cultivar	Leaf-feeding form

Sample code	Sampling location (lat, long)	Sampling year	Country[1]	Host plant	Phylloxera feeding form
CHE139	47.83, 8.97	2013	CHE	*Vitis vinifera* L. cultivar	Leaf-feeding form
CHE140	47.83, 8.97	2013	CHE	*Vitis vinifera* L. cultivar	Leaf-feeding form
CHE141	47.83, 8.97	2013	CHE	*Vitis vinifera* L. cultivar	Leaf-feeding form
CHE142	47.83, 8.97	2013	CHE	*Vitis vinifera* L. cultivar	Leaf-feeding form
CHE143	47.83, 8.97	2013	CHE	*Vitis vinifera* L. cultivar	Leaf-feeding form
CHE144	n.a.	2013	CHE	n.a.	n.a.
CHE145	n.a.	2013	CHE	n.a.	n.a.
CHE146	n.a.	2013	CHE	n.a.	n.a.
CHE147	n.a.	2013	CHE	n.a.	n.a.
CHE148	46.5, 6.67	n.a.	CHE	*Vitis* spp. interspecific hybrid direct-producer	Leaf-feeding form
CHE149	46.5, 6.67	n.a.	CHE	*Vitis* spp. interspecific hybrid direct-producer	Leaf-feeding form
CHE150	46.5, 6.67	n.a.	CHE	*Vitis* spp. interspecific hybrid direct-producer	Leaf-feeding form
CHE151	46.5, 6.67	n.a.	CHE	*Vitis* spp. interspecific hybrid direct-producer	Leaf-feeding form
CHE152	47.08, 7.15	2013	CHE	*Vitis* spp. interspecific rootstock	Root-feeding form
CHE153	47.08, 7.15	2013	CHE	*Vitis* spp. interspecific rootstock	Root-feeding form
CHE154	47.08, 7.15	2013	CHE	*Vitis* spp. interspecific rootstock	Root-feeding form
CHE155	47.08, 7.13	2013	CHE	*Vitis* spp. interspecific hybrid direct-producer	Leaf-feeding form
CHE156	47.08, 7.13	2013	CHE	*Vitis* spp. interspecific hybrid direct-producer	Leaf-feeding form
CHE157	47.08, 7.13	2013	CHE	*Vitis* spp. interspecific hybrid direct-producer	Leaf-feeding form
CHE158	47.08, 7.13	2013	CHE	*Vitis* spp. interspecific hybrid direct-producer	Leaf-feeding form
CHE159	47.08, 7.13	2013	CHE	*Vitis* spp. interspecific hybrid direct-producer	Leaf-feeding form
CHE160	47.07, 7.09	2013	CHE	*Vitis* spp. interspecific hybrid direct-producer	Leaf-feeding form
CHE161	47.07, 7.09	2013	CHE	*Vitis* spp. interspecific hybrid direct-producer	Leaf-feeding form
CHE162	47.07, 7.09	2013	CHE	*Vitis* spp. interspecific hybrid direct-producer	Leaf-feeding form
CHE163	47.07, 7.09	2013	CHE	*Vitis* spp. interspecific hybrid direct-producer	Leaf-feeding form
CHE164	47.07, 7.09	2013	CHE	*Vitis* spp. interspecific hybrid direct-producer	Leaf-feeding form
CHE165	47.08, 7.15	2013	CHE	*Vitis* spp. interspecific hybrid direct-producer	Leaf-feeding form

Sample code	Sampling location (lat, long)	Sampling year	Country[1]	Host plant	Phylloxera feeding form
CHE166	47.08, 7.15	2013	CHE	*Vitis* spp. interspecific hybrid direct-producer	Leaf-feeding form
CHE167	46.41, 6.23	n.a.	CHE	*Vitis* spp. interspecific rootstock	Root-feeding form
CHE168	46.41, 6.23	n.a.	CHE	*Vitis* spp. interspecific rootstock	Root-feeding form
CHE169	46.41, 6.23	n.a.	CHE	*Vitis* spp. interspecific rootstock	Root-feeding form
CHE170	46.41, 6.23	n.a.	CHE	*Vitis* spp. interspecific rootstock	Root-feeding form
CHE171	46.41, 6.23	n.a.	CHE	*Vitis* spp. interspecific rootstock	Root-feeding form
CHE172	46.41, 6.23	n.a.	CHE	*Vitis* spp. interspecific rootstock	Root-feeding form
CHE173	46.41, 6.23	n.a.	CHE	*Vitis* spp. interspecific rootstock	Root-feeding form
CHE174	47.53, 8.58	2013	CHE	*Vitis* spp. interspecific hybrid direct-producer	Leaf-feeding form
CHE175	47.53, 8.58	2013	CHE	*Vitis* spp. interspecific hybrid direct-producer	Leaf-feeding form
CHE176	47.53, 8.58	2013	CHE	*Vitis* spp. interspecific hybrid direct-producer	Leaf-feeding form
CHE177	47.53, 8.58	2013	CHE	*Vitis* spp. interspecific hybrid direct-producer	Leaf-feeding form
CHE178	47.53, 8.58	2013	CHE	*Vitis* spp. interspecific hybrid direct-producer	Leaf-feeding form
CHE179	47.53, 8.58	2013	CHE	*Vitis* spp. interspecific hybrid direct-producer	Root-feeding form
CHE180	47.53, 8.58	2013	CHE	*Vitis* spp. interspecific hybrid direct-producer	Root-feeding form
CHE181	47.53, 8.58	2013	CHE	*Vitis* spp. interspecific hybrid direct-producer	Root-feeding form
CHE182	47.53, 8.58	2013	CHE	*Vitis* spp. interspecific hybrid direct-producer	Root-feeding form
CHE183	47.53, 8.58	2013	CHE	*Vitis* spp. interspecific hybrid direct-producer	Root-feeding form
CHE184	47.53, 8.58	2013	CHE	*Vitis* spp. interspecific hybrid direct-producer	Root-feeding form
CHE185	47.53, 8.58	2013	CHE	*Vitis* spp. interspecific hybrid direct-producer	Root-feeding form
CHE186	47.63, 8.67	2013	CHE	*Vitis* spp. interspecific hybrid direct-producer	Leaf-feeding form
CHE187	47.63, 8.67	2013	CHE	*Vitis* spp. interspecific hybrid direct-producer	Leaf-feeding form
CHE188	47.63, 8.67	2013	CHE	*Vitis* spp. interspecific hybrid direct-producer	Leaf-feeding form
CHE189	47.63, 8.67	2013	CHE	*Vitis* spp. interspecific hybrid direct-producer	Leaf-feeding form
CHE190	47.63, 8.67	2013	CHE	*Vitis* spp. interspecific hybrid direct-producer	Leaf-feeding form
CHE191	47.63, 8.67	2013	CHE	*Vitis* spp. interspecific rootstock	Root-feeding form
CHE192	47.63, 8.67	2013	CHE	*Vitis* spp. interspecific rootstock	Root-feeding form

Sample code	Sampling location (lat, long)	Sampling year	Country[1]	Host plant	Phylloxera feeding form
CHE193	47.63, 8.67	2013	CHE	*Vitis* spp. interspecific rootstock	Root-feeding form
CHE194	47.63, 8.67	2013	CHE	*Vitis* spp. interspecific rootstock	Root-feeding form
CHE195	47.63, 8.67	2013	CHE	*Vitis* spp. interspecific rootstock	Root-feeding form
CHE196	47.63, 8.67	2013	CHE	*Vitis* spp. interspecific rootstock	Root-feeding form
CHE197	47.63, 8.67	2013	CHE	*Vitis* spp. interspecific rootstock	Root-feeding form
CHE198	47.63, 8.67	2013	CHE	*Vitis* spp. interspecific rootstock	Root-feeding form
CHE199	47.63, 8.67	2013	CHE	*Vitis* spp. interspecific rootstock	Root-feeding form
CHE200	47.63, 8.67	2013	CHE	*Vitis* spp. interspecific rootstock	Root-feeding form
CHE201	47.63, 8.67	2013	CHE	*Vitis* spp. interspecific hybrid direct-producer	Leaf-feeding form
CHE202	47.63, 8.67	2013	CHE	*Vitis* spp. interspecific hybrid direct-producer	Leaf-feeding form
CHE203	47.63, 8.67	2013	CHE	*Vitis* spp. interspecific hybrid direct-producer	Leaf-feeding form
CHE204	47.63, 8.67	2013	CHE	*Vitis* spp. interspecific hybrid direct-producer	Leaf-feeding form
CHE205	47.63, 8.67	2013	CHE	*Vitis* spp. interspecific hybrid direct-producer	Leaf-feeding form
CHE206	47.63, 8.67	2013	CHE	*Vitis* spp. interspecific hybrid direct-producer	Root-feeding form
CHE207	47.63, 8.67	2013	CHE	*Vitis* spp. interspecific hybrid direct-producer	Root-feeding form
CHE208	47.63, 8.67	2013	CHE	*Vitis* spp. interspecific hybrid direct-producer	Root-feeding form
CHE209	47.63, 8.67	2013	CHE	*Vitis* spp. interspecific hybrid direct-producer	Root-feeding form
CHE210	47.63, 8.67	2013	CHE	*Vitis* spp. interspecific hybrid direct-producer	Root-feeding form
CHE211	47.63, 8.67	2013	CHE	*Vitis* spp. interspecific hybrid direct-producer	Root-feeding form
CHE212	47.63, 8.67	2013	CHE	*Vitis* spp. interspecific hybrid direct-producer	Root-feeding form
CHE213	47.63, 8.67	2013	CHE	*Vitis* spp. interspecific hybrid direct-producer	Root-feeding form
CHE214	47.63, 8.67	2013	CHE	*Vitis* spp. interspecific hybrid direct-producer	Root-feeding form
CHE215	47.63, 8.67	2013	CHE	*Vitis* spp. interspecific hybrid direct-producer	Root-feeding form
CHE216	47.58, 8.8	2013	CHE	*Vitis* spp. interspecific hybrid direct-producer	Leaf-feeding form
CHE217	47.58, 8.8	2013	CHE	*Vitis* spp. interspecific hybrid direct-producer	Leaf-feeding form
CHE218	47.58, 8.8	2013	CHE	*Vitis* spp. interspecific hybrid direct-producer	Leaf-feeding form
CHE219	47.58, 8.8	2013	CHE	*Vitis* spp. interspecific hybrid direct-producer	Leaf-feeding form

Sample code	Sampling location (lat, long)	Sampling year	Country[1]	Host plant	Phylloxera feeding form
CHE220	47.58, 8.8	2013	CHE	*Vitis* spp. interspecific hybrid direct-producer	Leaf-feeding form
CHE221	47.58, 8.8	2013	CHE	*Vitis* spp. interspecific hybrid direct-producer	Leaf-feeding form
CHE222	47.58, 8.8	2013	CHE	*Vitis* spp. interspecific rootstock	Root-feeding form
CHE223	47.58, 8.8	2013	CHE	*Vitis* spp. interspecific rootstock	Root-feeding form
CHE224	47.58, 8.8	2013	CHE	*Vitis* spp. interspecific rootstock	Root-feeding form
CHE225	47.58, 8.8	2013	CHE	*Vitis* spp. interspecific rootstock	Root-feeding form
CHE226	47.58, 8.8	2013	CHE	*Vitis* spp. interspecific rootstock	Root-feeding form
CHE227	47.58, 8.8	2013	CHE	*Vitis* spp. interspecific rootstock	Root-feeding form
CHE228	47.58, 8.8	2013	CHE	*Vitis* spp. interspecific rootstock	Root-feeding form
CHE229	47.58, 8.8	2013	CHE	*Vitis* spp. interspecific rootstock	Root-feeding form
CHE230	47.58, 8.8	2013	CHE	*Vitis* spp. interspecific rootstock	Root-feeding form
CHE231	47.58, 8.8	2013	CHE	*Vitis* spp. interspecific rootstock	Root-feeding form
CHE232	47.7, 8.45	2013	CHE	*Vitis* spp. interspecific hybrid direct-producer	Leaf-feeding form
CHE233	47.7, 8.45	2013	CHE	*Vitis* spp. interspecific hybrid direct-producer	Leaf-feeding form
CHE234	47.7, 8.45	2013	CHE	*Vitis* spp. interspecific hybrid direct-producer	Leaf-feeding form
CHE235	47.7, 8.45	2013	CHE	*Vitis* spp. interspecific hybrid direct-producer	Leaf-feeding form
CHE236	47.7, 8.45	2013	CHE	*Vitis* spp. interspecific hybrid direct-producer	Leaf-feeding form
CHE237	47.7, 8.45	2013	CHE	*Vitis* spp. interspecific rootstock	Root-feeding form
CHE238	47.7, 8.45	2013	CHE	*Vitis* spp. interspecific rootstock	Root-feeding form
CHE239	47.7, 8.45	2013	CHE	*Vitis* spp. interspecific rootstock	Root-feeding form
CHE240	47.7, 8.45	2013	CHE	*Vitis* spp. interspecific rootstock	Root-feeding form
CHE241	47.7, 8.45	2013	CHE	*Vitis* spp. interspecific rootstock	Root-feeding form
CHE242	47.52, 8.62	2013	CHE	*Vitis* spp. interspecific hybrid direct-producer	Leaf-feeding form
CHE243	47.52, 8.62	2013	CHE	*Vitis* spp. interspecific hybrid direct-producer	Leaf-feeding form
CHE244	47.52, 8.62	2013	CHE	*Vitis* spp. interspecific hybrid direct-producer	Leaf-feeding form
CHE245	47.52, 8.62	2013	CHE	*Vitis* spp. interspecific hybrid direct-producer	Leaf-feeding form
CHE246	47.52, 8.62	2013	CHE	*Vitis* spp. interspecific hybrid direct-producer	Leaf-feeding form

Sample code	Sampling location (lat, long)	Sampling year	Country[1]	Host plant	Phylloxera feeding form
CHE247	47.52, 8.62	2013	CHE	*Vitis* spp. interspecific rootstock	Root-feeding form
CHE248	47.52, 8.62	2013	CHE	*Vitis* spp. interspecific rootstock	Root-feeding form
CHE249	47.52, 8.62	2013	CHE	*Vitis* spp. interspecific rootstock	Root-feeding form
CHE250	47.52, 8.62	2013	CHE	*Vitis* spp. interspecific rootstock	Root-feeding form
CHE251	47.52, 8.62	2013	CHE	*Vitis* spp. interspecific rootstock	Root-feeding form
CHE252	47.47, 8.43	2013	CHE	*Vitis* spp. interspecific hybrid direct-producer	Leaf-feeding form
CHE253	47.47, 8.43	2013	CHE	*Vitis* spp. interspecific hybrid direct-producer	Leaf-feeding form
CHE254	47.47, 8.43	2013	CHE	*Vitis* spp. interspecific hybrid direct-producer	Leaf-feeding form
CHE255	47.47, 8.43	2013	CHE	*Vitis* spp. interspecific hybrid direct-producer	Leaf-feeding form
CHE256	47.47, 8.43	2013	CHE	*Vitis* spp. interspecific hybrid direct-producer	Leaf-feeding form
CHE257	47.47, 8.43	2013	CHE	*Vitis* spp. interspecific hybrid direct-producer	Root-feeding form
CHE258	47.47, 8.43	2013	CHE	*Vitis* spp. interspecific hybrid direct-producer	Root-feeding form
CHE259	47.47, 8.43	2013	CHE	*Vitis* spp. interspecific hybrid direct-producer	Root-feeding form
CHE260	47.47, 8.43	2013	CHE	*Vitis* spp. interspecific hybrid direct-producer	Root-feeding form
CHE261	47.47, 8.43	2013	CHE	*Vitis* spp. interspecific hybrid direct-producer	Root-feeding form
CHE262	47.47, 8.43	2013	CHE	*Vitis* spp. interspecific hybrid direct-producer	Leaf-feeding form
CHE263	47.47, 8.43	2013	CHE	*Vitis* spp. interspecific hybrid direct-producer	Leaf-feeding form
CHE264	47.47, 8.43	2013	CHE	*Vitis* spp. interspecific hybrid direct-producer	Leaf-feeding form
CHE265	47.47, 8.43	2013	CHE	*Vitis* spp. interspecific hybrid direct-producer	Leaf-feeding form
CHE266	47.47, 8.43	2013	CHE	*Vitis* spp. interspecific hybrid direct-producer	Leaf-feeding form
CHE267	47.47, 8.43	2013	CHE	*Vitis* spp. interspecific rootstock	Root-feeding form
CHE268	47.47, 8.43	2013	CHE	*Vitis* spp. interspecific rootstock	Root-feeding form
CHE269	47.47, 8.43	2013	CHE	*Vitis* spp. interspecific rootstock	Root-feeding form
CHE270	47.47, 8.43	2013	CHE	*Vitis* spp. interspecific rootstock	Root-feeding form
CHE271	47.6, 8.52	2013	CHE	*Vitis* spp. interspecific hybrid direct-producer	Leaf-feeding form
CHE272	47.6, 8.52	2013	CHE	*Vitis* spp. interspecific hybrid direct-producer	Leaf-feeding form
CHE273	47.6, 8.52	2013	CHE	*Vitis* spp. interspecific hybrid direct-producer	Leaf-feeding form

Sample code	Sampling location (lat, long)	Sampling year	Country[1]	Host plant	Phylloxera feeding form
CHE274	47.6, 8.52	2013	CHE	*Vitis* spp. interspecific hybrid direct-producer	Leaf-feeding form
CHE275	47.6, 8.52	2013	CHE	*Vitis* spp. interspecific hybrid direct-producer	Leaf-feeding form
CHE276	47.6, 8.52	2013	CHE	*Vitis* spp. interspecific hybrid direct-producer	Root-feeding form
CHE277	47.6, 8.52	2013	CHE	*Vitis* spp. interspecific hybrid direct-producer	Leaf-feeding form
CHE278	47.6, 8.52	2013	CHE	*Vitis* spp. interspecific hybrid direct-producer	Leaf-feeding form
CHE279	47.6, 8.52	2013	CHE	*Vitis* spp. interspecific hybrid direct-producer	Leaf-feeding form
CHE280	47.6, 8.52	2013	CHE	*Vitis* spp. interspecific hybrid direct-producer	Leaf-feeding form
CHE281	47.68, 8.45	2013	CHE	*Vitis* spp. interspecific rootstock	Root-feeding form
CHE282	47.68, 8.45	2013	CHE	*Vitis* spp. interspecific rootstock	Root-feeding form
CHE283	47.68, 8.45	2013	CHE	*Vitis* spp. interspecific rootstock	Root-feeding form
URY001	n.a.	2018	URY	*Vitis vinifera* L. cultivar	Leaf-feeding form
URY002	n.a.	2018	URY	*Vitis vinifera* L. cultivar	Leaf-feeding form
URY003	n.a.	2018	URY	*Vitis vinifera* L. cultivar	Leaf-feeding form
URY004	n.a.	2018	URY	*Vitis vinifera* L. cultivar	Leaf-feeding form
URY005	n.a.	2018	URY	*Vitis vinifera* L. cultivar	Leaf-feeding form
URY006	n.a.	2018	URY	*Vitis vinifera* L. cultivar	Leaf-feeding form
URY007	n.a.	2018	URY	*Vitis vinifera* L. cultivar	Leaf-feeding form
URY008	n.a.	2018	URY	*Vitis vinifera* L. cultivar	Leaf-feeding form
URY009	n.a.	2018	URY	*Vitis vinifera* L. cultivar	Leaf-feeding form
URY010	n.a.	2018	URY	*Vitis vinifera* L. cultivar	Leaf-feeding form
URY011	n.a.	2018	URY	*Vitis vinifera* L. cultivar	Leaf-feeding form
URY012	n.a.	2018	URY	*Vitis vinifera* L. cultivar	Leaf-feeding form
URY013	n.a.	2018	URY	*Vitis vinifera* L. cultivar	Leaf-feeding form
URY014	n.a.	2018	URY	*Vitis vinifera* L. cultivar	Leaf-feeding form
URY015	n.a.	2018	URY	*Vitis vinifera* L. cultivar	Leaf-feeding form
URY016	n.a.	2018	URY	*Vitis vinifera* L. cultivar	Leaf-feeding form
URY017	n.a.	2018	URY	*Vitis vinifera* L. cultivar	Leaf-feeding form

Sample code	Sampling location (lat, long)	Sampling year	Country[1]	Host plant	Phylloxera feeding form
URY018	n.a.	2018	URY	*Vitis vinifera* L. cultivar	Leaf-feeding form
URY019	n.a.	2018	URY	*Vitis vinifera* L. cultivar	Leaf-feeding form
URY020	n.a.	2018	URY	*Vitis vinifera* L. cultivar	Leaf-feeding form
URY021	n.a.	2018	URY	*Vitis vinifera* L. cultivar	Leaf-feeding form
URY022	n.a.	2018	URY	*Vitis vinifera* L. cultivar	Leaf-feeding form
URY023	n.a.	2018	URY	*Vitis vinifera* L. cultivar	Leaf-feeding form
URY024	n.a.	2018	URY	*Vitis vinifera* L. cultivar	Leaf-feeding form
URY025	n.a.	2018	URY	*Vitis vinifera* L. cultivar	Leaf-feeding form
URY026	n.a.	2018	URY	*Vitis vinifera* L. cultivar	Leaf-feeding form
URY027	n.a.	2018	URY	*Vitis vinifera* L. cultivar	Leaf-feeding form
URY028	n.a.	2018	URY	*Vitis vinifera* L. cultivar	Leaf-feeding form
URY029	n.a.	2018	URY	*Vitis vinifera* L. cultivar	Leaf-feeding form
URY030	n.a.	2018	URY	*Vitis vinifera* L. cultivar	Leaf-feeding form
URY031	n.a.	2018	URY	*Vitis vinifera* L. cultivar	Leaf-feeding form
URY032	n.a.	2018	URY	*Vitis vinifera* L. cultivar	Leaf-feeding form
URY033	n.a.	2018	URY	*Vitis vinifera* L. cultivar	Leaf-feeding form
URY034	n.a.	2018	URY	*Vitis vinifera* L. cultivar	Leaf-feeding form
URY035	n.a.	2018	URY	*Vitis vinifera* L. cultivar	Leaf-feeding form
URY036	n.a.	2018	URY	*Vitis vinifera* L. cultivar	Leaf-feeding form
URY037	n.a.	2018	URY	*Vitis vinifera* L. cultivar	Leaf-feeding form
URY038	n.a.	2018	URY	*Vitis vinifera* L. cultivar	Leaf-feeding form
URY039	n.a.	2018	URY	*Vitis vinifera* L. cultivar	Leaf-feeding form
URY040	n.a.	2018	URY	*Vitis vinifera* L. cultivar	Leaf-feeding form
URY041	n.a.	2018	URY	*Vitis vinifera* L. cultivar	Leaf-feeding form
URY042	n.a.	2018	URY	*Vitis vinifera* L. cultivar	Leaf-feeding form
URY043	n.a.	2018	URY	*Vitis vinifera* L. cultivar	Leaf-feeding form
URY044	n.a.	2018	URY	*Vitis vinifera* L. cultivar	Leaf-feeding form

Sample code	Sampling location (lat, long)	Sampling year	Country[1]	Host plant	Phylloxera feeding form
SRB001	45.21, 19.75	2017	SRB	*Vitis* spp. interspecific resistant grape variety	Leaf-feeding form
SRB002	45.21, 19.75	2017	SRB	*Vitis* spp. interspecific resistant grape variety	Leaf-feeding form
SRB003	45.21, 19.75	2017	SRB	*Vitis* spp. interspecific resistant grape variety	Leaf-feeding form
SRB004	45.21, 19.75	2018	SRB	*Vitis* spp. interspecific resistant grape variety	Root-feeding form
SRB005	45.21, 19.75	2018	SRB	*Vitis* spp. interspecific resistant grape variety	Leaf-feeding form
SRB006	45.21, 19.75	2018	SRB	*Vitis* spp. interspecific resistant grape variety	Leaf-feeding form
SRB007	45.21, 19.75	2018	SRB	*Vitis* spp. interspecific resistant grape variety	Leaf-feeding form
SRB008	45.21, 19.75	2018	SRB	*Vitis* spp. interspecific resistant grape variety	Leaf-feeding form
SRB009	45.21, 19.75	2018	SRB	*Vitis* spp. interspecific resistant grape variety	Leaf-feeding form
SRB010	45.21, 19.75	2018	SRB	*Vitis* spp. interspecific resistant grape variety	Leaf-feeding form
SRB011	45.21, 19.75	2018	SRB	*Vitis* spp. interspecific resistant grape variety	Leaf-feeding form
SRB012	45.21, 19.75	2017	SRB	*Vitis* spp. interspecific rootstock	Root-feeding form
SRB013	45.21, 19.75	2017	SRB	*Vitis* spp. interspecific rootstock	Root-feeding form
SRB014	45.21, 19.75	2017	SRB	*Vitis* spp. interspecific rootstock	Leaf-feeding form
SRB015	45.21, 19.75	2017	SRB	*Vitis* spp. interspecific rootstock	Leaf-feeding form
SRB016	45.21, 19.75	2017	SRB	*Vitis* spp. interspecific rootstock	Root-feeding form
SRB017	45.21, 19.75	2017	SRB	*Vitis* spp. interspecific rootstock	Root-feeding form
SRB018	45.21, 19.75	2017	SRB	*Vitis* spp. interspecific rootstock	Leaf-feeding form
SRB019	45.21, 19.75	2017	SRB	*Vitis* spp. interspecific rootstock	Leaf-feeding form
SRB020	45.21, 19.75	2017	SRB	*Vitis* spp. interspecific rootstock	Leaf-feeding form
SRB021	45.21, 19.75	2017	SRB	*Vitis* spp. interspecific rootstock	Leaf-feeding form
SRB022	45.21, 19.75	2017	SRB	*Vitis* spp. interspecific rootstock	Leaf-feeding form
SRB023	45.21, 19.75	2018	SRB	*Vitis* spp. interspecific rootstock	Leaf-feeding form
SRB024	45.21, 19.75	2018	SRB	*Vitis* spp. interspecific rootstock	Leaf-feeding form
SRB025	45.21, 19.75	2018	SRB	*Vitis* spp. interspecific rootstock	Leaf-feeding form
SRB026	45.21, 19.75	2018	SRB	*Vitis* spp. interspecific rootstock	Leaf-feeding form
SRB027	45.21, 19.75	2018	SRB	*Vitis* spp. interspecific rootstock	Leaf-feeding form

**Sample code**	**Sampling location (lat, long)**	**Sampling year**	**Country[1]**	**Host plant**	**Phylloxera feeding form**
SRB028	45.21, 19.75	2018	SRB	*Vitis* spp. interspecific rootstock	Leaf-feeding form
SRB029	45.21, 19.75	2018	SRB	*Vitis* spp. interspecific rootstock	Leaf-feeding form
SRB030	45.21, 19.75	2018	SRB	*Vitis* spp. interspecific rootstock	Leaf-feeding form
SRB031	45.21, 19.75	2018	SRB	*Vitis vinifera* L. cultivar	Leaf-feeding form
SRB032	45.21, 19.75	2018	SRB	*Vitis vinifera* L. cultivar	Leaf-feeding form
SRB033	45.21, 19.75	2018	SRB	*Vitis vinifera* L. cultivar	Leaf-feeding form
SRB034	45.21, 19.75	2018	SRB	*Vitis vinifera* L. cultivar	Leaf-feeding form
SRB035	45.21, 19.75	2018	SRB	*Vitis vinifera* L. cultivar	Leaf-feeding form
SRB036	45.21, 19.75	2018	SRB	*Vitis* spp. interspecific resistant grape variety	Leaf-feeding form
SRB037	45.21, 19.75	2018	SRB	*Vitis* spp. interspecific resistant grape variety	Leaf-feeding form
SRB038	45.21, 19.75	2018	SRB	*Vitis* spp. interspecific resistant grape variety	Root-feeding form
SRB039	45.21, 19.75	2018	SRB	*Vitis vinifera* L. cultivar	Root-feeding form
ITA001	43.2, 12.07	2018	ITA	*Vitis* spp. interspecific rootstock	Leaf-feeding form
ITA002	43.2, 12.07	2018	ITA	*Vitis* spp. interspecific rootstock	Leaf-feeding form
ITA003	43.2, 12.07	2018	ITA	*Vitis* spp. interspecific rootstock	Leaf-feeding form
ITA004	43.2, 12.07	2018	ITA	*Vitis* spp. interspecific rootstock	Leaf-feeding form
ITA005	43.2, 12.07	2018	ITA	*Vitis* spp. interspecific rootstock	Leaf-feeding form
ITA006	43.2, 12.07	2018	ITA	*Vitis* spp. interspecific rootstock	Leaf-feeding form
ITA007	43.72, 11.2	2018	ITA	*Vitis* spp. interspecific rootstock	Leaf-feeding form
ITA008	43.72, 11.2	2018	ITA	*Vitis* spp. interspecific rootstock	Leaf-feeding form
ITA009	43.72, 11.2	2018	ITA	*Vitis* spp. interspecific rootstock	Leaf-feeding form
ITA010	43.72, 11.2	2018	ITA	*Vitis* spp. interspecific rootstock	Leaf-feeding form
ITA011	43.72, 11.2	2018	ITA	*Vitis* spp. interspecific rootstock	Leaf-feeding form
ITA012	43.01, 11.53	2018	ITA	*Vitis vinifera* L. cultivar	Leaf-feeding form
ITA013	43.01, 11.53	2018	ITA	*Vitis vinifera* L. cultivar	Leaf-feeding form
ITA014	43.01, 11.53	2018	ITA	*Vitis* spp. interspecific rootstock	Leaf-feeding form
ITA015	43.01, 11.53	2018	ITA	*Vitis* spp. interspecific rootstock	Leaf-feeding form

Sample code	Sampling location (lat, long)	Sampling year	Country[1]	Host plant	Phylloxera feeding form
ITA016	43.01, 11.53	2018	ITA	*Vitis* spp. interspecific rootstock	Leaf-feeding form
ITA017	43.07, 11.5	2018	ITA	*Vitis vinifera* L. cultivar	Leaf-feeding form
ITA018	43.07, 11.5	2018	ITA	*Vitis vinifera* L. cultivar	Leaf-feeding form
ITA019	43.07, 11.5	2018	ITA	*Vitis vinifera* L. cultivar	Leaf-feeding form
ITA020	43.07, 11.5	2018	ITA	*Vitis vinifera* L. cultivar	Leaf-feeding form
ITA021	43.07, 11.5	2018	ITA	*Vitis vinifera* L. cultivar	Leaf-feeding form
ITA022	43.72, 11.2	2018	ITA	*Vitis* spp. interspecific rootstock	Leaf-feeding form
ITA023	43.72, 11.2	2018	ITA	*Vitis* spp. interspecific rootstock	Leaf-feeding form
ITA024	43.72, 11.2	2018	ITA	*Vitis* spp. interspecific rootstock	Leaf-feeding form
ITA025	43.72, 11.2	2018	ITA	*Vitis* spp. interspecific rootstock	Leaf-feeding form
ITA026	43.72, 11.2	2018	ITA	*Vitis* spp. interspecific rootstock	Leaf-feeding form
ITA027	43.01, 11.53	2018	ITA	*Vitis* spp. interspecific rootstock	Leaf-feeding form
ITA028	43.01, 11.53	2018	ITA	*Vitis* spp. interspecific rootstock	Leaf-feeding form
ITA029	43.01, 11.53	2018	ITA	*Vitis* spp. interspecific rootstock	Leaf-feeding form
ITA030	43.01, 11.53	2018	ITA	*Vitis* spp. interspecific rootstock	Leaf-feeding form
ITA031	43.01, 11.53	2018	ITA	*Vitis* spp. interspecific rootstock	Leaf-feeding form
ITA032	43.72, 11.2	2018	ITA	*Vitis* spp. interspecific rootstock	Leaf-feeding form
ITA033	43.72, 11.2	2018	ITA	*Vitis* spp. interspecific rootstock	Leaf-feeding form
ITA034	43.72, 11.2	2018	ITA	*Vitis* spp. interspecific rootstock	Leaf-feeding form
ITA035	43.72, 11.2	2018	ITA	*Vitis* spp. interspecific rootstock	Leaf-feeding form
ITA036	43.72, 11.2	2018	ITA	*Vitis* spp. interspecific rootstock	Leaf-feeding form
ITA037	43.72, 11.2	2018	ITA	*Vitis* spp. interspecific rootstock	Leaf-feeding form
ITA038	43.72, 11.2	2018	ITA	*Vitis* spp. interspecific rootstock	Leaf-feeding form
ITA039	43.72, 11.2	2018	ITA	*Vitis* spp. interspecific rootstock	Leaf-feeding form
ITA040	43.72, 11.2	2018	ITA	*Vitis* spp. interspecific rootstock	Leaf-feeding form
ITA041	43.72, 11.2	2018	ITA	*Vitis* spp. interspecific rootstock	Leaf-feeding form
ITA042	43.2, 12.07	2018	ITA	*Vitis* spp. interspecific rootstock	Leaf-feeding form

Sample code	Sampling location (lat, long)	Sampling year	Country[1]	Host plant	Phylloxera feeding form
ITA043	43.2, 12.07	2018	ITA	*Vitis* spp. interspecific rootstock	Leaf-feeding form
ITA044	43.2, 12.07	2018	ITA	*Vitis* spp. interspecific rootstock	Leaf-feeding form
ITA045	43.2, 12.07	2018	ITA	*Vitis* spp. interspecific rootstock	Leaf-feeding form
ITA046	43.2, 12.07	2018	ITA	*Vitis* spp. interspecific rootstock	Leaf-feeding form
ITA047	43.2, 12.07	2018	ITA	*Vitis* spp. interspecific rootstock	Leaf-feeding form
ITA048	45.89, 11.04	2018	ITA	*Vitis* spp. interspecific rootstock	Leaf-feeding form
ITA049	45.89, 11.04	2018	ITA	*Vitis* spp. interspecific rootstock	Leaf-feeding form
ITA050	45.89, 11.04	2018	ITA	*Vitis* spp. interspecific rootstock	Leaf-feeding form
ITA051	45.89, 11.04	2018	ITA	*Vitis* spp. interspecific rootstock	Leaf-feeding form
ITA052	45.89, 11.04	2018	ITA	*Vitis* spp. interspecific rootstock	Leaf-feeding form
ITA053	45.44, 10.99	2017	ITA	*Vitis vinifera* L. cultivar	Leaf-feeding form
ITA054	45.44, 10.99	2017	ITA	*Vitis vinifera* L. cultivar	Leaf-feeding form
ITA055	45.44, 10.99	2017	ITA	*Vitis vinifera* L. cultivar	Leaf-feeding form
ITA056	45.44, 10.99	2017	ITA	*Vitis vinifera* L. cultivar	Leaf-feeding form
ITA057	45.44, 10.99	2017	ITA	*Vitis vinifera* L. cultivar	Leaf-feeding form
ITA058	45.44, 10.99	2017	ITA	*Vitis vinifera* L. cultivar	Leaf-feeding form
ITA059	45.44, 10.99	2017	ITA	*Vitis vinifera* L. cultivar	Leaf-feeding form
ITA060	45.44, 10.99	2017	ITA	*Vitis vinifera* L. cultivar	Leaf-feeding form
ITA061	n.a.	2017	ITA	*Vitis vinifera* L. cultivar	Leaf-feeding form
ITA062	n.a.	2017	ITA	*Vitis vinifera* L. cultivar	Leaf-feeding form
ITA063	45.47, 10.61	2017	ITA	*Vitis vinifera* L. cultivar	Leaf-feeding form
ITA064	45.47, 10.61	2017	ITA	*Vitis vinifera* L. cultivar	Leaf-feeding form
ITA065	45.44, 10.99	2017	ITA	*Vitis vinifera* L. cultivar	Leaf-feeding form
ITA066	45.44, 10.99	2017	ITA	*Vitis vinifera* L. cultivar	Leaf-feeding form
ITA067	45.44, 10.99	2017	ITA	*Vitis vinifera* L. cultivar	Leaf-feeding form
ITA068	45.44, 10.99	2017	ITA	*Vitis vinifera* L. cultivar	Leaf-feeding form
ITA069	45.44, 10.99	2017	ITA	*Vitis vinifera* L. cultivar	Leaf-feeding form

Sample code	Sampling location (lat, long)	Sampling year	Country[1]	Host plant	Phylloxera feeding form
ITA070	45.44, 10.99	2017	ITA	*Vitis vinifera* L. cultivar	Leaf-feeding form
ITA071	45.42, 11.25	2017	ITA	*Vitis vinifera* L. cultivar	Leaf-feeding form
ITA072	45.42, 11.25	2017	ITA	*Vitis vinifera* L. cultivar	Leaf-feeding form
ITA073	45.42, 11.25	2017	ITA	*Vitis vinifera* L. cultivar	Leaf-feeding form
ITA074	45.42, 11.25	2017	ITA	*Vitis vinifera* L. cultivar	Leaf-feeding form
ITA075	45.42, 11.25	2017	ITA	*Vitis vinifera* L. cultivar	Leaf-feeding form
ITA076	45.42, 11.25	2017	ITA	*Vitis vinifera* L. cultivar	Leaf-feeding form
ITA077	45.42, 11.25	2017	ITA	*Vitis vinifera* L. cultivar	Leaf-feeding form
ITA078	45.42, 11.25	2017	ITA	*Vitis vinifera* L. cultivar	Leaf-feeding form
ITA079	45.42, 11.25	2017	ITA	*Vitis vinifera* L. cultivar	Leaf-feeding form
ZAF001	-33.55, 18.96	2019	ZAF	*Vitis* spp. interspecific rootstock	Leaf-feeding form
ZAF002	-33.55, 18.96	2019	ZAF	*Vitis* spp. interspecific rootstock	Leaf-feeding form
ZAF003	-33.55, 18.96	2019	ZAF	*Vitis* spp. interspecific rootstock	Leaf-feeding form
ZAF004	-33.55, 18.96	2019	ZAF	*Vitis* spp. interspecific rootstock	Leaf-feeding form
ZAF005	-33.55, 18.96	2019	ZAF	*Vitis* spp. interspecific rootstock	Leaf-feeding form
ZAF006	-33.55, 18.96	2019	ZAF	*Vitis* spp. interspecific rootstock	Leaf-feeding form
ZAF007	-33.55, 18.96	2019	ZAF	*Vitis* spp. interspecific rootstock	Leaf-feeding form
ZAF008	-33.55, 18.96	2019	ZAF	*Vitis* spp. interspecific rootstock	Leaf-feeding form
HUN001	47.69, 19.75	2016	HUN	*Vitis* spp. interspecific rootstock	Leaf-feeding form
HUN002	n.a.	2016	HUN	*Vitis* spp. interspecific rootstock	Leaf-feeding form
HUN003	n.a.	2016	HUN	*Vitis* spp. interspecific rootstock	Leaf-feeding form
HUN004	47.69, 19.75	2016	HUN	*Vitis* spp. interspecific rootstock	Leaf-feeding form
HUN005	47.69, 19.75	2016	HUN	*Vitis* spp. interspecific rootstock	Leaf-feeding form
HUN006	47.69, 19.75	2016	HUN	*Vitis* spp. interspecific rootstock	Leaf-feeding form
HUN007	47.01, 18.26	2016	HUN	*Vitis* spp. interspecific rootstock	Root-feeding form
HUN008	47.01, 18.26	2016	HUN	*Vitis* spp. interspecific rootstock	Leaf-feeding form
HUN009	47.01, 18.26	2016	HUN	*Vitis* spp. interspecific rootstock	Leaf-feeding form

Sample code	Sampling location (lat, long)	Sampling year	Country[1]	Host plant	Phylloxera feeding form
HUN010	47.01, 18.26	2016	HUN	*Vitis* spp. interspecific rootstock	Leaf-feeding form
HUN011	47, 17.99	2016	HUN	*Vitis* spp. interspecific rootstock	Root-feeding form
HUN012	47, 17.99	2016	HUN	*Vitis* spp. interspecific rootstock	Root-feeding form
HUN013	47, 17.99	2016	HUN	*Vitis* spp. interspecific rootstock	Root-feeding form
HUN014	47, 17.99	2016	HUN	*Vitis* spp. interspecific rootstock	Leaf-feeding form
HUN015	47, 17.99	2016	HUN	*Vitis* spp. interspecific rootstock	Leaf-feeding form
HUN016	47, 17.99	2016	HUN	*Vitis* spp. interspecific rootstock	Leaf-feeding form
HUN017	46.89, 17.61	2016	HUN	*Vitis* spp. interspecific rootstock	Root-feeding form
HUN018	46.89, 17.61	2016	HUN	*Vitis* spp. interspecific rootstock	Root-feeding form
HUN019	46.89, 17.61	2016	HUN	*Vitis* spp. interspecific rootstock	Root-feeding form
HUN020	46.89, 17.61	2016	HUN	*Vitis* spp. interspecific rootstock	Root-feeding form
HUN021	46.89, 17.61	2016	HUN	*Vitis* spp. interspecific rootstock	Leaf-feeding form
HUN022	46.89, 17.61	2016	HUN	*Vitis* spp. interspecific rootstock	Leaf-feeding form
HUN023	46.89, 17.61	2016	HUN	*Vitis* spp. interspecific rootstock	Leaf-feeding form
HUN024	46.89, 17.61	2016	HUN	*Vitis* spp. interspecific rootstock	Leaf-feeding form
HUN025	47.68, 16.62	2016	HUN	*Vitis* spp. interspecific rootstock	Root-feeding form
HUN026	47.68, 16.62	2016	HUN	*Vitis* spp. interspecific rootstock	Root-feeding form
HUN027	47.68, 16.62	2016	HUN	*Vitis* spp. interspecific rootstock	Root-feeding form
HUN028	47.68, 16.62	2016	HUN	*Vitis* spp. interspecific rootstock	Leaf-feeding form
HUN029	47.68, 16.62	2016	HUN	*Vitis* spp. interspecific rootstock	Leaf-feeding form
HUN030	47.45, 18.73	2016	HUN	*Vitis* spp. interspecific rootstock	Leaf-feeding form
HUN031	47.45, 18.73	2016	HUN	*Vitis* spp. interspecific rootstock	Leaf-feeding form
HUN032	47.45, 18.73	2016	HUN	*Vitis* spp. interspecific rootstock	Leaf-feeding form
HUN033	47.49, 18.63	2016	HUN	*Vitis* spp. interspecific rootstock	Root-feeding form
HUN034	47.49, 18.63	2016	HUN	*Vitis* spp. interspecific rootstock	Root-feeding form
HUN035	47.49, 18.63	2016	HUN	*Vitis* spp. interspecific rootstock	Leaf-feeding form
HUN036	47.49, 18.63	2016	HUN	*Vitis* spp. interspecific rootstock	Leaf-feeding form

Sample code	Sampling location (lat, long)	Sampling year	Country[1]	Host plant	Phylloxera feeding form
HUN037	47.49, 18.63	2016	HUN	*Vitis* spp. interspecific rootstock	Leaf-feeding form
HUN038	47.49, 18.63	2016	HUN	*Vitis* spp. interspecific rootstock	Leaf-feeding form
HUN039	46.09, 18.29	2016	HUN	*Vitis* spp. interspecific rootstock	Leaf-feeding form
HUN040	46.09, 18.29	2016	HUN	*Vitis* spp. interspecific rootstock	Leaf-feeding form
HUN041	46.36, 18.69	2016	HUN	*Vitis* spp. interspecific rootstock	Root-feeding form
HUN042	46.36, 18.69	2016	HUN	*Vitis* spp. interspecific rootstock	Root-feeding form
HUN043	46.36, 18.69	2016	HUN	*Vitis* spp. interspecific rootstock	Root-feeding form
HUN044	46.36, 18.69	2016	HUN	*Vitis* spp. interspecific rootstock	Root-feeding form
HUN045	46.36, 18.69	2016	HUN	*Vitis* spp. interspecific rootstock	Root-feeding form
HUN046	46.36, 18.69	2016	HUN	*Vitis* spp. interspecific rootstock	Leaf-feeding form
HUN047	46.36, 18.69	2016	HUN	*Vitis* spp. interspecific rootstock	Leaf-feeding form
HUN048	46.2, 18.97	2016	HUN	*Vitis* spp. interspecific rootstock	Root-feeding form
HUN049	46.2, 18.97	2016	HUN	*Vitis* spp. interspecific rootstock	Root-feeding form
HUN050	46.2, 18.97	2016	HUN	*Vitis* spp. interspecific rootstock	Leaf-feeding form
HUN051	47.91, 20.35	2016	HUN	*Vitis* spp. interspecific rootstock	Leaf-feeding form
HUN052	48.12, 21.41	2016	HUN	*Vitis* spp. interspecific rootstock	Leaf-feeding form
HUN053	48.12, 21.41	2016	HUN	*Vitis* spp. interspecific rootstock	Leaf-feeding form
HUN054	48.12, 21.41	2016	HUN	*Vitis* spp. interspecific rootstock	Leaf-feeding form
HUN055	47.55, 21.59	2016	HUN	*Vitis* spp. interspecific rootstock	Leaf-feeding form
HUN056	47.55, 21.59	2016	HUN	*Vitis* spp. interspecific rootstock	Leaf-feeding form
HUN057	46.94, 19.65	2016	HUN	*Vitis* spp. interspecific rootstock	Leaf-feeding form
HUN058	46.94, 19.65	2016	HUN	*Vitis* spp. interspecific rootstock	Leaf-feeding form
HUN059	47.58, 19.37	2016	HUN	*Vitis* spp. interspecific rootstock	Root-feeding form
HUN060	47.58, 19.37	2016	HUN	*Vitis* spp. interspecific rootstock	Root-feeding form
HUN061	47.58, 19.37	2016	HUN	*Vitis* spp. interspecific rootstock	Root-feeding form
HUN062	47.58, 19.37	2016	HUN	*Vitis* spp. interspecific rootstock	Root-feeding form
HUN063	47.58, 19.37	2016	HUN	*Vitis* spp. interspecific rootstock	Root-feeding form

Sample code	Sampling location (lat, long)	Sampling year	Country[1]	Host plant	Phylloxera feeding form
HUN064	47.58, 19.37	2016	HUN	*Vitis* spp. interspecific rootstock	Leaf-feeding form
HUN065	47.49, 18.91	2016	HUN	*Vitis* spp. interspecific rootstock	Root-feeding form
HUN066	47.49, 18.91	2016	HUN	*Vitis* spp. interspecific rootstock	Root-feeding form
HUN067	47.49, 18.91	2016	HUN	*Vitis* spp. interspecific rootstock	Root-feeding form
HUN068	47.49, 18.91	2016	HUN	*Vitis* spp. interspecific rootstock	Root-feeding form
HUN069	47.49, 18.91	2016	HUN	*Vitis* spp. interspecific rootstock	Root-feeding form
HUN070	47.49, 18.91	2016	HUN	*Vitis* spp. interspecific rootstock	Leaf-feeding form
HUN071	47.49, 18.91	2016	HUN	*Vitis* spp. interspecific rootstock	Leaf-feeding form
HUN072	47.49, 18.91	2016	HUN	*Vitis* spp. interspecific rootstock	Leaf-feeding form
HUN073	47.49, 18.91	2016	HUN	*Vitis* spp. interspecific rootstock	Leaf-feeding form
HUN074	47.56, 17.76	2016	HUN	*Vitis* spp. interspecific rootstock	Leaf-feeding form
HUN075	47.56, 17.76	2016	HUN	*Vitis* spp. interspecific rootstock	Leaf-feeding form
HUN076	47.56, 17.76	2016	HUN	*Vitis* spp. interspecific rootstock	Leaf-feeding form
HUN077	47.56, 17.76	2016	HUN	*Vitis* spp. interspecific rootstock	Leaf-feeding form
HUN078	47.84, 20.24	2016	HUN	*Vitis* spp. interspecific rootstock	Root-feeding form
HUN079	47.84, 20.24	2016	HUN	*Vitis* spp. interspecific rootstock	Root-feeding form
HUN080	47.84, 20.24	2016	HUN	*Vitis* spp. interspecific rootstock	Root-feeding form
HUN081	47.84, 20.24	2016	HUN	*Vitis* spp. interspecific rootstock	Leaf-feeding form
HUN082	47.69, 19.75	2016	HUN	*Vitis* spp. interspecific rootstock	Root-feeding form
HUN083	47.01, 18.26	2016	HUN	*Vitis* spp. interspecific rootstock	Root-feeding form
HUN084	47.91, 20.35	2016	HUN	*Vitis* spp. interspecific rootstock	Root-feeding form
HUN085	47.49, 18.63	2016	HUN	*Vitis* spp. interspecific rootstock	Root-feeding form
HUN086	46.09, 18.29	2016	HUN	*Vitis* spp. interspecific rootstock	Root-feeding form
HUN087	46.09, 18.29	2016	HUN	*Vitis* spp. interspecific rootstock	Root-feeding form
HUN088	46.09, 18.29	2016	HUN	*Vitis* spp. interspecific rootstock	Root-feeding form
HUN089	47.8, 20.35	2016	HUN	*Vitis* spp. interspecific rootstock	Root-feeding form
HUN090	47.8, 20.35	2016	HUN	*Vitis* spp. interspecific rootstock	Root-feeding form

Sample code	Sampling location (lat, long)	Sampling year	Country[1]	Host plant	Phylloxera feeding form
HUN091	47.8, 20.35	2016	HUN	*Vitis* spp. interspecific rootstock	Root-feeding form
HUN092	47.91, 20.35	2016	HUN	*Vitis* spp. interspecific rootstock	Root-feeding form
HUN093	47.55, 21.59	2016	HUN	*Vitis* spp. interspecific rootstock	Root-feeding form
HUN094	47.55, 21.59	2016	HUN	*Vitis* spp. interspecific rootstock	Root-feeding form
HUN095	47.55, 21.59	2016	HUN	*Vitis* spp. interspecific rootstock	Root-feeding form
HUN096	46.94, 19.65	2016	HUN	*Vitis* spp. interspecific rootstock	Root-feeding form
HUN097	46.94, 19.65	2016	HUN	*Vitis* spp. interspecific rootstock	Root-feeding form
HUN098	46.94, 19.65	2016	HUN	*Vitis* spp. interspecific rootstock	Root-feeding form
HUN099	47.56, 17.76	2016	HUN	*Vitis* spp. interspecific rootstock	Root-feeding form
HUN100	47.56, 17.76	2016	HUN	*Vitis* spp. interspecific rootstock	Root-feeding form
HUN101	47.56, 17.76	2016	HUN	*Vitis* spp. interspecific rootstock	Root-feeding form
HUN102	47.84, 20.24	2016	HUN	*Vitis* spp. interspecific rootstock	Leaf-feeding form
HRV001	45.81, 15.98	2015	HRV	n.a.	n.a.
HRV002	45.81, 15.98	2015	HRV	n.a.	n.a.
HRV003	45.81, 15.98	2015	HRV	n.a.	n.a.
HRV004	45.81, 15.98	2015	HRV	n.a.	n.a.
HRV005	45.81, 15.98	2015	HRV	n.a.	n.a.
HRV006	45.81, 15.98	2015	HRV	n.a.	n.a.
HRV007	45.81, 15.98	2015	HRV	n.a.	n.a.
HRV008	45.81, 15.98	2015	HRV	n.a.	n.a.
HRV009	45.81, 15.98	2015	HRV	n.a.	n.a.
HRV010	45.81, 15.98	2015	HRV	*Vitis vinifera* L. cultivar	Leaf-feeding form
HRV011	45.81, 15.98	2015	HRV	*Vitis vinifera* L. cultivar	Leaf-feeding form
HRV012	45.81, 15.98	2015	HRV	*Vitis vinifera* L. cultivar	Leaf-feeding form
HRV013	45.81, 15.98	2015	HRV	*Vitis vinifera* L. cultivar	Leaf-feeding form
HRV014	45.73, 15.72	2015	HRV	*Vitis* spp. interspecific rootstock	Leaf-feeding form
HRV015	45.73, 15.72	2015	HRV	*Vitis* spp. interspecific rootstock	Leaf-feeding form

Sample code	Sampling location (lat, long)	Sampling year	Country[1]	Host plant	Phylloxera feeding form
HRV016	45.73, 15.72	2015	HRV	*Vitis* spp. interspecific rootstock	Leaf-feeding form
HRV017	45.73, 15.72	2015	HRV	*Vitis* spp. interspecific rootstock	Leaf-feeding form
HRV018	45.73, 15.72	2015	HRV	*Vitis* spp. interspecific rootstock	Leaf-feeding form
HRV019	45.73, 15.72	2015	HRV	*Vitis* spp. interspecific rootstock	Leaf-feeding form
HRV020	45.73, 15.72	2015	HRV	*Vitis* spp. interspecific rootstock	Leaf-feeding form
HRV021	45.72, 15.72	2015	HRV	*Vitis* spp. interspecific rootstock	Leaf-feeding form
HRV022	45.72, 15.72	2015	HRV	*Vitis* spp. interspecific rootstock	Leaf-feeding form
HRV023	45.72, 15.72	2015	HRV	*Vitis* spp. interspecific rootstock	Leaf-feeding form
HRV024	45.72, 15.7	2015	HRV	*Vitis* spp. interspecific rootstock	Leaf-feeding form
HRV025	45.72, 15.7	2015	HRV	*Vitis* spp. interspecific rootstock	Leaf-feeding form
HRV026	45.72, 15.7	2015	HRV	*Vitis* spp. interspecific rootstock	Leaf-feeding form
HRV027	45.72, 15.7	2015	HRV	*Vitis* spp. interspecific rootstock	Leaf-feeding form
HRV028	45.72, 15.7	2015	HRV	*Vitis* spp. interspecific rootstock	Leaf-feeding form
HRV029	45.72, 15.7	2015	HRV	*Vitis* spp. interspecific rootstock	Leaf-feeding form
HRV030	45.72, 15.7	2015	HRV	*Vitis* spp. interspecific rootstock	Leaf-feeding form
HRV031	45.72, 15.7	2015	HRV	*Vitis* spp. interspecific rootstock	Leaf-feeding form
HRV032	45.72, 15.7	2015	HRV	*Vitis* spp. interspecific rootstock	Leaf-feeding form
HRV033	45.72, 15.7	2015	HRV	*Vitis* spp. interspecific rootstock	Leaf-feeding form
HRV034	45.72, 15.7	2015	HRV	*Vitis* spp. interspecific rootstock	Leaf-feeding form
HRV035	45.72, 15.7	2015	HRV	*Vitis* spp. interspecific rootstock	Leaf-feeding form
HRV036	45.72, 15.7	2015	HRV	*Vitis* spp. interspecific rootstock	Leaf-feeding form
HRV037	45.71, 15.72	2015	HRV	*Vitis* spp. interspecific rootstock	Leaf-feeding form
HRV038	45.71, 15.72	2015	HRV	*Vitis* spp. interspecific rootstock	Leaf-feeding form
HRV039	45.71, 15.72	2015	HRV	*Vitis* spp. interspecific rootstock	Leaf-feeding form
HRV040	45.71, 15.72	2015	HRV	*Vitis* spp. interspecific rootstock	Leaf-feeding form
HRV041	45.71, 15.72	2015	HRV	*Vitis* spp. interspecific rootstock	Leaf-feeding form
HRV042	45.71, 15.72	2015	HRV	*Vitis* spp. interspecific rootstock	Leaf-feeding form

Sample code	Sampling location (lat, long)	Sampling year	Country[1]	Host plant	Phylloxera feeding form
HRV043	45.71, 15.72	2015	HRV	*Vitis* spp. interspecific rootstock	Leaf-feeding form
HRV044	45.71, 15.72	2015	HRV	*Vitis* spp. interspecific rootstock	Leaf-feeding form
HRV045	45.74, 15.69	2015	HRV	*Vitis* spp. interspecific rootstock	Leaf-feeding form
HRV046	45.74, 15.69	2015	HRV	*Vitis* spp. interspecific rootstock	Leaf-feeding form
HRV047	45.89, 17.19	2015	HRV	*Vitis* spp. interspecific rootstock	Leaf-feeding form
HRV048	45.89, 17.19	2015	HRV	*Vitis* spp. interspecific rootstock	Leaf-feeding form
HRV049	45.89, 17.19	2015	HRV	*Vitis* spp. interspecific rootstock	Leaf-feeding form
HRV050	45.89, 17.19	2015	HRV	*Vitis* spp. interspecific rootstock	Leaf-feeding form
HRV051	45.89, 17.19	2015	HRV	*Vitis* spp. interspecific rootstock	Leaf-feeding form
HRV052	45.22, 13.58	2015	HRV	*Vitis vinifera* L. cultivar	Leaf-feeding form
HRV053	45.22, 13.65	2015	HRV	*Vitis* spp. interspecific rootstock	Leaf-feeding form
HRV054	45.22, 13.65	2015	HRV	*Vitis* spp. interspecific rootstock	Leaf-feeding form
HRV055	45.21, 13.69	2015	HRV	*Vitis* spp. interspecific rootstock	Leaf-feeding form
HRV056	45.21, 13.69	2015	HRV	*Vitis* spp. interspecific rootstock	Leaf-feeding form
HRV057	45.21, 13.69	2015	HRV	*Vitis* spp. interspecific rootstock	Leaf-feeding form
HRV058	45.21, 13.69	2015	HRV	*Vitis* spp. interspecific rootstock	Leaf-feeding form
HRV059	45.21, 13.69	2015	HRV	*Vitis* spp. interspecific rootstock	Leaf-feeding form
HRV060	45.21, 13.69	2015	HRV	*Vitis* spp. interspecific rootstock	Leaf-feeding form
HRV061	45.21, 13.69	2015	HRV	*Vitis* spp. interspecific rootstock	Leaf-feeding form
HRV062	45.21, 13.69	2015	HRV	*Vitis* spp. interspecific rootstock	Leaf-feeding form
HRV063	45.21, 13.69	2015	HRV	*Vitis* spp. interspecific rootstock	Leaf-feeding form
HRV064	45.21, 13.69	2015	HRV	*Vitis* spp. interspecific rootstock	Leaf-feeding form
HRV065	45.21, 13.69	2015	HRV	*Vitis* spp. interspecific rootstock	Leaf-feeding form
HRV066	45.21, 13.69	2015	HRV	*Vitis* spp. interspecific rootstock	Leaf-feeding form
HRV067	45.21, 13.69	2015	HRV	*Vitis* spp. interspecific rootstock	Leaf-feeding form
HRV068	45.21, 13.69	2015	HRV	*Vitis* spp. interspecific rootstock	Leaf-feeding form
HRV069	45.21, 13.69	2015	HRV	*Vitis* spp. interspecific rootstock	Leaf-feeding form

Sample code	Sampling location (lat, long)	Sampling year	Country[1]	Host plant	Phylloxera feeding form
HRV070	45.09, 14.64	2015	HRV	*Vitis* spp. interspecific rootstock	Leaf-feeding form
HRV071	45.07, 14.66	2015	HRV	*Vitis* spp. interspecific rootstock	Leaf-feeding form
HRV072	45.07, 14.66	2015	HRV	*Vitis* spp. interspecific rootstock	Leaf-feeding form
HRV073	45.07, 14.66	2015	HRV	*Vitis* spp. interspecific rootstock	Leaf-feeding form
HRV074	45.07, 14.66	2015	HRV	*Vitis* spp. interspecific rootstock	Leaf-feeding form
HRV075	45.07, 14.66	2015	HRV	*Vitis* spp. interspecific rootstock	Leaf-feeding form
HRV076	45.07, 14.66	2015	HRV	*Vitis* spp. interspecific rootstock	Leaf-feeding form
HRV077	45.07, 14.66	2015	HRV	*Vitis* spp. interspecific rootstock	Leaf-feeding form
HRV078	45.59, 15.92	2015	HRV	*Vitis vinifera* L. cultivar	Leaf-feeding form
HRV079	45.59, 15.92	2015	HRV	*Vitis vinifera* L. cultivar	Leaf-feeding form
HRV080	45.59, 15.92	2015	HRV	*Vitis vinifera* L. cultivar	Leaf-feeding form
HRV081	45.59, 15.92	2015	HRV	*Vitis vinifera* L. cultivar	Leaf-feeding form
HRV082	45.59, 15.92	2015	HRV	*Vitis vinifera* L. cultivar	Leaf-feeding form
HRV083	45.59, 15.92	2015	HRV	*Vitis vinifera* L. cultivar	Leaf-feeding form
HRV084	45.59, 15.92	2015	HRV	*Vitis vinifera* L. cultivar	Leaf-feeding form
HRV085	45.59, 15.92	2015	HRV	*Vitis vinifera* L. cultivar	Leaf-feeding form
HRV086	45.59, 15.92	2015	HRV	*Vitis vinifera* L. cultivar	Leaf-feeding form
HRV087	45.59, 15.92	2015	HRV	*Vitis vinifera* L. cultivar	Leaf-feeding form
HRV088	45.59, 15.92	2015	HRV	*Vitis vinifera* L. cultivar	Leaf-feeding form
HRV089	45.82, 15.59	2015	HRV	*Vitis vinifera* L. cultivar	Leaf-feeding form
HRV090	45.82, 15.59	2015	HRV	*Vitis vinifera* L. cultivar	Leaf-feeding form
HRV091	45.82, 15.59	2015	HRV	*Vitis vinifera* L. cultivar	Leaf-feeding form
HRV092	45.82, 15.59	2015	HRV	*Vitis vinifera* L. cultivar	Leaf-feeding form
HRV093	45.82, 15.59	2015	HRV	*Vitis vinifera* L. cultivar	Leaf-feeding form
HRV094	45.82, 15.59	2015	HRV	*Vitis vinifera* L. cultivar	Leaf-feeding form
HRV095	45.82, 15.59	2015	HRV	*Vitis vinifera* L. cultivar	Leaf-feeding form
HRV096	43.08, 17.62	2015	HRV	*Vitis vinifera* L. cultivar	Leaf-feeding form

**Sample code**	**Sampling location (lat, long)**	**Sampling year**	**Country[1]**	**Host plant**	**Phylloxera feeding form**
HRV097	43.08, 17.62	2015	HRV	*Vitis vinifera* L. cultivar	Leaf-feeding form
HRV098	43.03, 17.59	2015	HRV	*Vitis vinifera* L. cultivar	Leaf-feeding form
HRV099	43.03, 17.59	2015	HRV	*Vitis vinifera* L. cultivar	Leaf-feeding form
HRV100	43.03, 17.59	2015	HRV	*Vitis vinifera* L. cultivar	Leaf-feeding form
HRV101	43.03, 17.59	2015	HRV	*Vitis vinifera* L. cultivar	Leaf-feeding form
HRV102	43.03, 17.59	2015	HRV	*Vitis vinifera* L. cultivar	Leaf-feeding form
HRV103	43.03, 17.59	2015	HRV	*Vitis vinifera* L. cultivar	Leaf-feeding form
HRV104	43.03, 17.59	2015	HRV	*Vitis vinifera* L. cultivar	Leaf-feeding form
HRV105	43.02, 17.57	2015	HRV	*Vitis vinifera* L. cultivar	Leaf-feeding form
HRV106	43.02, 17.57	2015	HRV	*Vitis vinifera* L. cultivar	Leaf-feeding form
HRV107	43.02, 17.57	2015	HRV	*Vitis vinifera* L. cultivar	Leaf-feeding form
ROU001	47.16, 27.59	2016	ROU	*Vitis* spp. interspecific rootstock	Leaf-feeding form
ROU002	47.16, 27.59	2016	ROU	*Vitis* spp. interspecific rootstock	Leaf-feeding form
ROU003	47.16, 27.59	2016	ROU	*Vitis* spp. interspecific rootstock	Leaf-feeding form

[1]: AUT: Austria; CHE: Switzerland; DEU: Germany; HUN: Hungary; HRV: Croatia; ITA: Italy; ROU: Romania; SRB: Serbia; URY: Uruguay; ZAF: South Africa
